# Hypomorphic Protein Expression of DNA Polymerase Beta in Polβ^L301R-V303R/L301R-V303R^ Knock-In Transgenic Mice Does Not Impact Global DNA Methylation Levels in the Midbrain

**DOI:** 10.3390/biom16030412

**Published:** 2026-03-11

**Authors:** Bryce Jacobs, Dan Ivanov, Ivana Barraza, Christopher Faulk, Carmen J. Booth, Raquel Mattos-Canedo, Lucas Tian, Kaitlyn DePietro, Alper Uzun, Wynand P. Roos, Laurie H. Sanders, Robert W. Sobol

**Affiliations:** 1 Department of Pathology and Laboratory Medicine, Warren Alpert Medical School & Legorreta Cancer Center, Brown University, Providence, RI 02912, USA; bryce_jacobs@alumni.brown.edu (B.J.); daniel_ivanov@brown.edu (D.I.); irina_mattos-canedo@brown.edu (R.M.-C.); lucas_tian@brown.edu (L.T.); kaitlyn_depietro@brown.edu (K.D.); alper_uzun@brown.edu (A.U.); wynand_roos@brown.edu (W.P.R.); 2 Departments of Neurology and Pathology, Duke University, Durham, NC 27708, USA; ivana.barraza@duke.edu (I.B.); laurie.sanders@duke.edu (L.H.S.); 3 Duke Center for Neurodegeneration and Neurotherapeutics, Duke University, Durham, NC 27710, USA; 4 Department of Animal Sciences, University of Minnesota, Minneapolis, MN 55455, USA; cfaulk@umn.edu; 5 Department of Comparative Medicine, Yale University School of Medicine, New Haven, CT 06520, USA; carmen.booth@yale.edu

**Keywords:** DNA polymerase beta, demethylation, base excision repair, Xrcc1

## Abstract

DNA polymerase beta (Polβ) is a 39 kDa, single polypeptide enzyme that possesses both gap tailoring and nucleotidyl transferase activity and is the key polymerase involved in base excision repair (BER) and the final steps of active gene demethylation. We demonstrated that residues in the mouse Polβ protein, L301 and V303, are critical for Polβ’s interaction with the BER scaffolding protein X-ray repair cross-complementing 1 (XRCC1), and mutation of these residues impairs Polβ’s ability to bind to XRCC1, negatively impacting BER complex assembly. We developed Polβ^L301R-V303R/L301R-V303R^ knock-in mice to explore how defects with this essential protein complex impact genome stability in the mouse. We found these mice to be viable and fertile yet exhibited a modest reduction in body weight. Here, we examined the protein and mRNA levels in tissues from wild-type (WT), heterozygous (HET), and homozygous (HOM) Polβ^L301R-V303R/L301R-V303R^ mice and the derived fibroblast cell lines. We show that HOM mice have significantly diminished Polβ protein levels, as compared to WT mice, in several tissues, yet Polβ mRNA levels were not significantly different, suggesting the decreased levels of Polβ protein could not be attributed to lower gene expression. Upon examination of Polβ stability in mouse ear fibroblasts derived from WT and HOM mice, results are consistent with human cell studies that the Polβ^L301R-V303R^ protein is unstable and undergoes proteasome-mediated degradation. Finally, we evaluated WT, and HOM, liver and brain genomic DNA samples for 5-methylcytosine/5-hydroxymethylcytosine (5mC/5hmC) levels by nanopore sequencing to investigate the impact of suppressed Polβ protein levels on active gene demethylation. As expected, we found tissue-specific trends in methylation, when comparing the brain and liver. However, we were unable to discern substantial differences in methylation levels between WT and HOM mice, suggesting that in the absence of external stressors, low Polβ levels do not impact methylation patterns.

## 1. Introduction

Base excision repair (BER) is a critical DNA repair pathway that is important for the maintenance of a healthy genome. Importantly, defects in BER may have implications for a variety of clinical settings, including the response to chemotherapy [1,2,3,4,5,6], the etiology of cancer [5,7,8], neurodegeneration [9,10,11,12], and aging [13,14,15,16,17]. BER, which consists of both short-patch and long-patch pathways, is responsible for repairing an estimated 20,000 base lesions per cell per day in the nuclear and mitochondrial genomes [13,18,19,20,21,22,23,24]. BER consists of three generalized steps: Lesion Recognition/Strand Scission; DNA Gap Tailoring; and DNA Synthesis/Ligation [13,25,26,27]. All BER sub-pathways involve the repair of either base lesions or a single-strand break (SSB) and begin with the recognition of the lesion by a particular molecular detector, which includes mono-functional DNA glycosylases [24,28], bi-functional DNA glycosylases [25,29], AP endonuclease 1 (APE1) [30,31], or poly(ADP-ribose)polymerase 1 (PARP1) [32,33], depending on the lesion type [13]. In addition, there is a TDP1-dependent SSB repair mechanism that involves the repair of trapped Top1-DNA complexes [34,35]. After the strand is hydrolyzed, scaffolding and signaling proteins, mainly X-ray repair cross-complementing 1 (XRCC1) and PARP1, facilitate the formation of a BER protein complex [36,37,38] to complete repair [39].

As an essential component of the BER protein complex, DNA polymerase beta (Polβ), a 39 kDa single polypeptide, conducts the gap tailoring and gap filling processes of BER to repair damaged DNA bases [40,41,42,43] and facilitates gene demethylation [44,45,46,47]. Its 8 kDa N-terminal domain possesses the gap tailoring ability via its 5′-deoxyribose phosphate (5′dRP) lyase activity, whereas the 31 kDa C-terminal domain catalyzes gap filling via its nucleotidyl transferase activity and consists of thumb, palm, and finger domains [48,49,50]. In the canonical model of BER, Polβ gap tailoring is preceded by APE1 strand scission [13,51,52]. While Polβ is primarily implicated in short-patch BER, it also plays a role in long-patch BER [13,53,54,55,56,57].

The formation of protein complexes and protein–protein interactions is crucial for progression through the BER pathway [13,58,59,60,61,62]. Protein complexes that assemble throughout the BER process are variable depending on the class of DNA damage, and they play a critical role in the transfer of substrates and products from reaction to reaction to minimize the accumulation of toxic BER pathway intermediates [58,63,64,65].

One particularly significant protein–protein interaction in the BER pathway is between Polβ and XRCC1. The interaction occurs between the C-terminal domain of Polβ and the N-terminal domain of XRCC1 [66]. Binding of XRCC1 to Polβ is critical for proper BER functioning and is enhanced by XRCC1 oxidation [67]. Localization of Polβ to sites of laser-induced DNA damage depends on its interaction with XRCC1 [68,69]. In particular, three amino acid residues encoded by Polβ—L301, V303, and V306—are especially integral to the Polβ-XRCC1 interface [66,67,70,71]. Mutation of these three residues in mouse embryonic fibroblasts (MEFs) inhibited binding to XRCC1, as shown by the diminished peak recruitment intensity of Polβ to sites of laser-induced DNA damage [72]. Importantly, there was no significant difference in peak recruitment intensity of Polβ, hence no difference in binding to XRCC1, between cells expressing the double mutant Polβ(L301R/V303R) or cells expressing the triple mutant Polβ(L301R/V303R/V306R)—in both cases, recruitment to sites of DNA damage was abolished. This suggested that L301 and V303, in particular, are integral to the formation of the mouse Polβ/XRCC1 protein complex [72]. Structural analysis of this Polβ/XRCC1 complex suggests that the hydrophobicity of L301 and V303 is necessary for binding, and that loss of hydrophobicity results in a direct clash of proline 2 (P2) of oxidized XRCC1 with L303R [70,72].

To further investigate the implications of the Polβ-XRCC1 interface on murine physiology, fertility, and viability, we developed a homozygous L301R/V303R CRISPR/Cas9-modified knock-in (KI) mouse model [72]. Even though perinatal lethality is observed in Polβ-knockout (Polβ-KO) mice, Polβ^L301R-V303R/L301R-V303R^ KI mice were viable and fertile yet exhibited a 15% decrease in body weight as compared to their WT counterparts [72,73]. Further, ear fibroblasts derived from the Polβ-KI mice showed an 85% decrease in Polβ protein expression via immunoblot quantification analysis as compared to WT fibroblasts [72]. However, Polβ-KI fibroblasts did not demonstrate significant hypersensitivity to genotoxic stress from chronic exposure to either H_2_O_2_ or MMS [72]. These findings are consistent with experiments conducted with the Polβ(L301R/V303R) mutant protein expressed in human glioblastoma LN428 cells, which did not exhibit hypersensitivity to genotoxic stress despite heavily diminished Polβ levels [74]. As a result, it is proposed that the resulting low levels of Polβ are sufficient for the response to DNA damage in fibroblasts. Further, Polβ’s interaction with the oxidized form of XRCC1 is likely responsible for regulating Polβ stability and subsequent proteasomal degradation [67,72,74,75].

The development of viable mouse models that have impacted Polβ levels is crucial [76,77,78], especially given the implications of BER, and specifically Polβ, in the pathogenesis of several age-related diseases [9], including neurodegeneration [9,10,79,80,81]. Genome-wide mapping in human neuronal cells showed that post-mitotic neuronal enhancers and sites of DNA demethylation are hotspots of DNA single-strand breaks (SSBs), which are repaired by XRCC1-dependent BER [79]. To initiate demethylation, the ten eleven–translocation (TET) family of dioxygenases oxidize 5mC to several intermediates of DNA demethylation, including 5-hydroxymethylcytosine (5hmC), 5-formylcytosine (5fC), and 5-carboxylcytosine (5caC) [82,83]. Both 5fC and 5caC are substrates for the DNA glycosylase TDG [84], implicating BER in the active demethylation mechanism [85,86,87]. Once the glycosylase removes the 5fC or 5caC lesion [88], and the AP-site is processed by APE1, Polβ removes the 5′-deoxyribose-5-phosphate group and inserts an unmethylated cytosine [89]. To complete the demethylation process, the nick is sealed by LigI/LigIII. Such active DNA demethylation allows reprogramming in the mouse germ line [90], facilitating removal of the epigenetic mark 5mC, as well as the oxidized derivatives [91]. Interestingly, this BER-mediated active demethylation mechanism has been shown to involve covalent PARylation [92], as well as SUMOylation [93] and is negatively impacted by PARylation [94]. BER also plays a critical role in regulating the neural epigenome by controlling cytosine demethylation, especially given the programmed DNA damage pathways that exist in neurons [95]. It also plays a critical role in repairing neuronal DNA oxidative damage, one of the main mechanisms of DNA damage reported in age-related neurodegeneration [10,96].

Specifically, Polβ is likely the principal DNA polymerase in post-mitotic neurons [10,44,97,98,99,100]. Previously, a mouse model was created by crossing a common Alzheimer’s Disease (AD) model (3xTg) [101] with a model heterozygous for Polβ (Polβ^+/−^) [43,73,76], yielding the 3xTg/Polβ^+/−^ mouse model [102]. This mouse model exhibited aggravated AD pathology characterized by neuronal dysfunction, increased cell death, memory impairment, and decreased synaptic plasticity, as well as a transcriptional profile nearly identical to that of human AD patients [102]. Further, mice with a disrupted Polβ gene exhibited significant apoptotic cell death at birth, in the central and peripheral nervous systems, and especially in post-mitotic neurons [78], dependent on p53 [103]. Therefore, it has been suggested that Polβ is involved in regulating neural development and that a deficiency in Polβ induces more severe neurodegeneration in an AD model.

Given the potential role of Polβ in abrogating neurodegeneration, the development and characterization of novel Polβ mouse models is critical [73]. While Polβ-KO mouse models are not viable past birth and die perinatally [43,73,76], the use of the Polβ^L301R-V303R/L301R-V303R^ KI mouse model is compelling because it offers an opportunity to investigate the impacts of diminished Polβ levels and altered BER in vivo. Although ear fibroblasts derived from these hypomorphic KI mice showed an ~85% decrease in Polβ levels [72], the Polβ protein and mRNA levels throughout different tissues in vivo are unknown. As a result, a thorough characterization of the L301R/V303R KI mouse is necessary.

In this study, we characterized wild-type (WT, Polβ^WT/WT^), heterozygous (HET, Polβ^WT/L301R-V303R^), and homozygous (HOM, Polβ^L301R-V303R/L301R-V303R^) KI mice by examining Polβ mRNA and protein expression levels in a variety of tissues, investigating potential mechanisms underlying any differences in Polβ protein levels observed, and using genome skimming analysis to probe for differences in methylation patterns between organs and mouse genotypes.

## 2. Materials and Methods

### 2.1. Reagents and Chemicals

All chemicals, reagents, antibodies, and other resources used in this study are listed in Table S1.

### 2.2. Cells and Cell Culture

Immortalized mouse ear fibroblast cells derived from Polβ^L301R-V303R/L301R-V303R^ and WT littermate mice were generated as previously described [64,104]. Fibroblasts were cultured in RPMI 1640 supplemented with 10% heat-inactivated fetal bovine serum (FBS), 1% antibiotic–antimycotic, and 1% penicillin/streptomycin. Cells were grown in tissue culture incubators at 37 °C and 5% CO_2_.

### 2.3. Polβ Stability Assay and Cell Protein Extract Preparation

To investigate the stability of Polβ expressed by immortalized ear fibroblast cells derived from Polβ^L301R-V303R/L301R-V303R^ or littermate WT mice, 5 × 10^5^ cells were seeded in 10 cm dishes. After 24 h, Polβ^L301R-V303R/L301R-V303R^ cells or littermate WT cells were treated for 6 h with either DMSO (control), 35.5 µM cycloheximide (cyclo) only, 25 µM of the proteasome inhibitor MG132 only, or 35.5 µM cyclo combined with 25 µM MG132. Cycloheximide (Sigma Aldrich, St. Louis, MO, USA) was received as a powder and was prepared as a 12.5 mM stock solution in DMSO, and MG132 (Selleck Chemicals, Houston, TX, USA) was received and used as a 10 mM stock solution in DMSO. After the 6 h treatment, drugs were removed, and cells were washed once with DPBS (Corning, Corning, NY, USA). Whole cell lysates were prepared with 2X clear Laemmli buffer (2% SDS, 20% glycerol, 715 mM 2-mercaptoethanol, 62.5 mM Tris-HCl pH 6.8). Total protein concentration was determined with the absorption of each sample at 280nm (A280) using the NanoDrop 2000 Spectrophotometer (Thermo Scientific, Waltham, WA, USA). Polβ levels were determined using immunoblot, with histone H3 levels used as a loading control as described below.

### 2.4. Polβ^L301R-V303R/L301R-V303R^ Mouse Generation and Genotyping

The Polβ^WT/L301R-V303R^ mouse was created by the University of Alabama Birmingham (UAB) Transgenic and Genetically Engineered Models (TGEM) Core as previously described [72]. Polβ^WT/L301R-V303R^ mice were bred to C57BL/6 mice (Jackson Labs, Bar Harbor, ME, USA), and Polβ^WT/L301R-V303R^ pups from different litters were interbred to establish litters of WT, heterozygous (HET, Polβ^WT/L301R-V303R^), and homozygous (HOM, Polβ^L301R-V303R/L301R-V303R^) offspring. All animal breeding was conducted in accordance with institutional guidelines at Brown University (IACUC Protocol # 5014-23).

To identify WT, HET, and HOM offspring after weaning, DNA isolated from pup tail biopsies using the DNeasy Blood & Tissue Kit (Qiagen, Germantown, MD, USA) was PCR amplified and then processed using XcmI restriction digestion. The sequences of the primers used for PCR amplification of the Polβ allele were 5′-GCAGCCTCATCCTCACCAATAAG-3′ (Forward) and 5′-GAAATAACTTCTGCCTTACTCACCATC-3′ (Reverse).

Approximately 100 ng of gDNA from the tail (1 µL) was mixed with 25 µL 2X OneTaq Master Mix with Standard Buffer (NEB, Ipswich, MA, USA), 22 µL qRT–PCR grade water (Invitrogen, Carlsbad, CA, USA), and 1 µL of 10µM each primer (IDT, Coralville, IA, USA). The PCR was run on the PTC Tempo Thermal Cycler (Bio-Rad Laboratories, Hercules, CA, USA) using the program described previously [72]. Samples were denatured at 94 °C for 30 s, then subjected to 33 cycles of denaturation (94 °C, 30 s), annealing (60 °C, 1 min), and extension (68 °C, 1 min and 15 s), followed by a final extension step (68 °C, 5 min). A total of 12 µL of each PCR amplicon was then digested with 0.8 µL XcmI Restriction Endonuclease (NEB), 3 µL NEBuffer Restriction Digest Buffer (NEB), and 15 µL qRT–PCR grade water (Invitrogen) by incubation at 37 °C for 50 min followed by a 5 min digestion at 85 °C. The samples were then separated by electrophoresis on a 2% agarose gel [2g SeaKem LE Agarose (Lonza, Lexington, MA, USA), 100 mL 1X Tris-Borate-EDTA (TBE) Buffer (Thermo Scientific), supplemented with ethidium bromide (0.5 µg/mL)] and electrophoresed in 1X TBE buffer (Thermo Scientific). The WT genotype exhibited 3 bands at 507bp, 441bp, and 205bp, the HET genotype exhibited 4 bands at 946bp, 507bp, 441bp, and 205bp, and the HOM genotype exhibited 2 bands at 946bp and 205bp.

### 2.5. Mouse Brain and Liver Isolation

Mouse Cohort #1 comprised twenty-four (24) mice between 6 and 9 months of age (8 WT, 8 HET, 8 HOM). Each was sacrificed via asphyxiation with CO_2_ followed by cervical dislocation. Following euthanization, livers and brains were immediately isolated. A small sample of each liver and brain was stabilized in 1 mL Allprotect Tissue Reagent (Qiagen), while the rest was fixed in 10% neutral buffered formalin (Lab Chem, Zelienople, PA, USA) for 24 h and then transferred into 70% ethanol. All brain samples were taken from the left hemisphere, with no additional specificity.

### 2.6. Whole Mouse Phenotyping: Histopathologic and Complete Blood Count

Mouse Cohort #2 comprised four WT (2M, 2F) and 8 HOM (4M, 4F) live mice, 10–15 weeks of age, bred from HET parents. Each was submitted to the Comparative Pathology Research (CPR) Core (Yale School of Medicine, Department of Comparative Medicine, New Haven, CT, USA) for comprehensive phenotyping. Mice were examined for gross macroscopic congenital defects, then rendered unconscious by CO_2_ asphyxiation, weighed, and euthanized by exsanguination by terminal cardiac puncture. The blood was placed in standard lavender top blood tubes with EDTA and submitted to Antech Diagnostic Laboratory (New York, NY, USA) for a complete blood count. The mice were necropsied blind to experimental genotype according to the standard protocol described on the CPR Core website (https://research.yale.edu/cores/whole-mouse-phenotyping, 18 January 2026), where the mice were examined for gross (macroscopic) defects. All tissues are immersion fixed in 10% Neutral Buffered Formalin (NBF), except for a small piece of liver, spleen, heart apex, kidney, and cerebellum, which were placed into Allprotect Tissue Reagent (Qiagen, Germantown, MD, USA) per the manufacturer’s recommendation. The head with the brain in situ (skin removed), one rear leg (skin removed), and the sternum were fixed in 10% NBF and subsequently decalcified using Decal Solution (Yale CPR Core, New Haven, CT, USA). The tissues were trimmed, placed in cassettes, processed, embedded, sectioned at 5 µm, and stained with hematoxylin and eosin (H&E; Yale CPR Core, New Haven, CT, USA), and cover-slipped by routine methods. The slides were examined blind to experimental genotypes using a BX53 microscope (Olympus, Center Valley, PA, USA) for pathologic and congenital microscopic changes.

### 2.7. Mouse Brain Microdissection, Fixation, and Cryopreservation

Mouse Cohort #3 comprised twelve (12) mice between 20 and 24 months of age (6 WT, 6 HOM) and were sacrificed via asphyxiation with CO_2_ followed by cervical dislocation. Following euthanization, brains were immediately isolated, washed once in 1X DPBS (Corning, Corning, NY, USA), and micro-dissected on ice as described [105].

To obtain cerebral hemispheres, brains were sliced in half on a sagittal plane. The left hemisphere was immediately placed in a freshly prepared 4% paraformaldehyde solution (Electron Microscopy Sciences, Hatfield, PA, USA) in 1X DPBS for 24 h at 4 °C. Brains were subsequently rinsed in 1X DPBS and transferred to a 30% sucrose solution (Sigma, St. Louis, MO, USA) in 1X DPBS at 4 °C until cryopreservation. Cryoprotected hemispheres were frozen on dry ice in disposable base molds (Fisher Scientific, Waltham, MA, USA) using Tissue-Tek Optimal Cutting Temperature (O.C.T.) compound (Sakura Finetek, Torrance, CA, USA) and stored at −80 °C.

The right hemisphere was micro-dissected on ice for specific brain regions, including the cerebellum, the ventral midbrain (rich in dopaminergic neurons [105,106,107,108]), the striatum, and the cortex. Upon isolation, micro-dissected brain regions were stabilized in 1 mL Allprotect Tissue Reagent (Qiagen) and stored at −80 °C until DNA, RNA, and protein isolation.

### 2.8. DNA, RNA, and Protein Isolation

Approximately 10 mg of each organ sample stabilized in Allprotect Tissue Reagent was disrupted and homogenized using the TissueLyser III system (Qiagen) in Buffer RLT (Qiagen) containing 150 mM 2-mercaptoethanol (Sigma). DNA, RNA, and protein were isolated from the tissue homogenates using the Allprep DNA/RNA/Protein Mini Kit (Qiagen). Isolations were conducted according to steps detailed in the Allprep DNA/RNA/Protein Handbook (Qiagen), except for the use of a 5% sodium dodecyl sulfate (SDS) solution to ensure optimal protein lysate resuspension. Isolated DNA was stored at −30 °C, while isolated RNA and protein samples were stored at −80 °C.

### 2.9. Immunoblots

Samples for immunoblot analysis were prepared with the appropriate volumes of protein lysates, 2X clear Laemmli buffer, and 4X Laemmli sample buffer (Bio-Rad). Protein lysate samples from mouse organs or cells (25–30 µg) were loaded onto precast NuPAGE 4–12% Bis-Tris gels (Invitrogen) and electrophoresed for 2 h at 100 V. Proteins separated by gel electrophoresis were transferred onto a 0.2 µm nitrocellulose membrane (Bio-Rad) for 18 min using the Trans-Blot Turbo system (Bio-Rad) in transfer buffer [20% 5X Transfer Buffer (Bio-Rad), 20% 200-proof ethanol, 60% nanopore water]. Membranes were blocked with TBST (TBS buffer with 0.05% Tween-20) + 5% blotting grade non-fat dry milk (Bio-Rad) for 1 h at room temperature, then exposed to the primary antibodies in TBST + 5% milk overnight at 4 °C. The primary antibodies and their dilutions are listed in Table S1. Membranes were then washed 3 × 10 min in TBST. Following washing, membranes were exposed to secondary antibody in TBST for 2 h, as listed in Table S1. After a second washing, membranes were either visualized with the Clarity Western ECL Peroxide and Luminol/Enhancer reagents (Bio-Rad) or, for less abundant proteins, the SuperSignal West Femto Luminol/Enhancer and Stable Peroxide reagents (Thermo Scientific). Histone H3 was used as a loading control. Membranes were imaged using the ChemiDoc MP imaging system (Bio-Rad). Band intensity was quantified using ImageJ software (Image J 1.48v).

### 2.10. Quantitative RT-PCR Analysis

Expression of Polβ, XRCC1, and β-actin mRNA was measured by quantitative RT-PCR (qRT-PCR) using the QuantStudio 7 system (Thermo Fisher Scientific). Following RNA isolation from brain and liver samples of Mouse Cohort #1 (<12-month-old mice), cDNA was synthesized using the SuperScript VILO cDNA Synthesis Kit (Invitrogen). 1000 ng of murine liver RNA or 100 ng of murine brain RNA was added to the reaction mixture and run on the PTC Tempo Thermal Cycler (Bio-Rad Laboratories) for 10 min at 25 °C, 60 min at 42 °C, and 5 min at 85 °C. Analysis of mRNA expression was performed with the ΔΔCT method using Applied Biosystems TaqMan Gene Expression Assays (Applied Biosystems, Foster City, CA, USA): MM00448234_M1 (mouse Polβ) and MM00494222_M1 (mouse Xrcc1). Samples were run in quadruplicate, and the results shown are the mean ± standard deviation (SD) of 8 biological replicates for WT, HET, and HOM livers and brains. Each sample was normalized to the expression of murine β-actin (MN02619580_G1).

### 2.11. Genome Skimming and Analysis

Genome skimming was performed essentially as described [109]. The samples were assembled as a barcoded library (with kit SQK-NBD114-9; Nanopore, Oxford, UK) and were skimmed using the Nanopore P2 Solo instrument (Oxford, UK). 5-methylcytosine (5mC) and 5-hydroxymethylcytosine (5hmC) levels were measured by averaging the modified CpG sites. First, BAM files containing 5mC and 5hmC reads were aligned to the mouse reference genome GRCm39 with dorado version 0.8.0, sorted, and indexed using Samtools (v. 1.22). Modkit version 0.4.1 was used to summarize modifications by genomic position into a bedmethyl file. Averages were calculated by dividing the count of methylated or hydroxymethylated cytosines in CpG context by the total number of cytosines in CpG context with an awk script.

### 2.12. Statistical Analysis

Means and standard deviation (SD) were calculated from the means of multiple independent experiments, including both technical and biological replicates. *t*-tests or one- or two-way analysis of variance (ANOVA) tests were used to detect significant differences between groups as seen fit, with treatment groups generally being compared to controls and/or as otherwise stated in the figure legends. *p*-values are indicated by asterisks (* *p* < 0.05, ** *p* < 0.01, *** *p* < 0.001, **** *p* < 0.0001). All statistical analyses were performed using GraphPad Prism (v. 10.6.1).

## 3. Results

### 3.1. Polβ Protein Levels Were Diminished in Various Polβ^L301R-V303R/L301R-V303R^ Mouse Tissues

**Figure 1:**
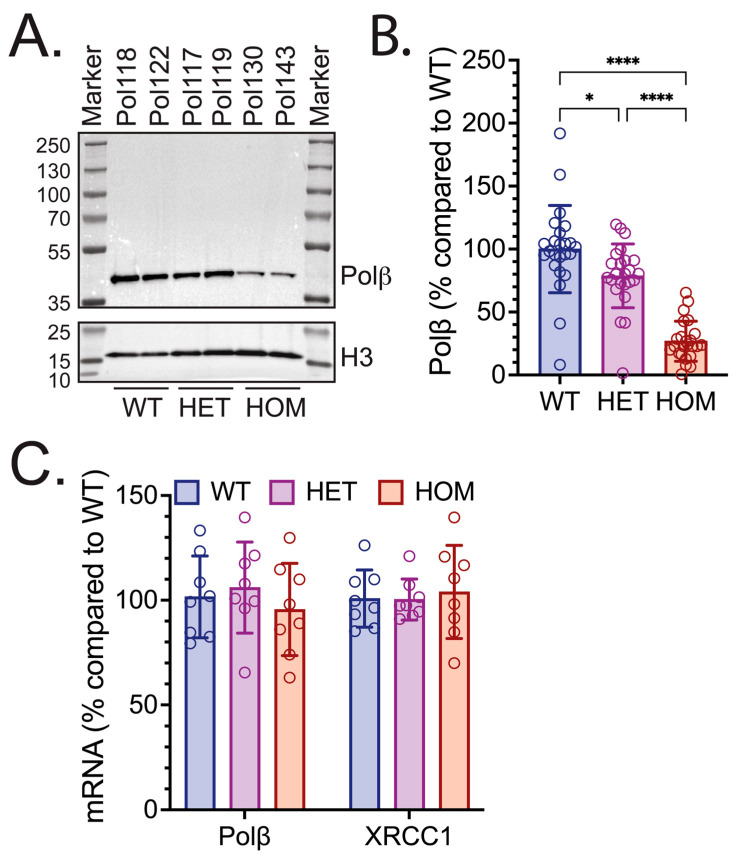
Polβ protein and mRNA levels in livers of WT, HET, and HOM Polβ^L301R-V303R/L301R-V303R^ KI mice. (**A**) Immunoblot of liver protein lysates from WT, HET, and HOM mice (Mouse Cohort #1), probing for Polβ. Sample names above each lane indicate the mouse identification number, with the genotype listed below. A representative immunoblot image is shown, which includes protein lysates from the livers of two mice of each genotype. Histone H3 was used as a loading control. (**B**) Protein level of Polβ in the livers of WT, HET, and HOM mice (Mouse Cohort #1). Protein level is shown as a % compared to WT. Quantifications are from immunoblot analysis, as in panel (**A**) and Supplementary Figure S1. Each bar represents 24 values (8 mice and 3 technical replicates per mouse), and each data point represents an independent immunoblot band. Values presented are mean ± SD. Significant differences are indicated by asterisks (* *p* < 0.05, **** *p* < 0.0001). (**C**) mRNA expression of Polβ and Xrcc1 isolated from livers of WT, HET, and HOM mice (Mouse Cohort #1). Results represent % changes in gene expression relative to WT, calculated using the ΔΔCT method with β-actin as an endogenous control. Each bar represents the mean ± SD of eight mice, and each data point represents the average of the samples run in quadruplicate.

It was previously reported that ear fibroblast cells derived from HOM L301/V303R KI mice exhibited 15% Polβ protein levels as compared to WT ear fibroblast cells [72]. As a result, we investigated whether these diminished Polβ protein levels were observed in vivo in tissues isolated from Polβ^L301R-V303R/L301R-V303R^ mice. Liver samples were isolated from WT, HET, and HOM mice (Mouse Cohort #1), disrupted and homogenized, and processed to yield liver protein lysates. Immunoblot analysis of mouse liver protein lysates showed that Polβ protein levels were visibly reduced in HOM mice (Figure 1A, two rightmost samples). Densitometry quantification of all liver immunoblots (Figure S1) with ImageJ revealed that Polβ protein levels were 26.8% in the livers of HOM mice, and 72.2% in the livers of HET mice, as compared to WT littermates (Figure 1B). The differences in means of Polβ levels between WT and HET mice and between WT and HOM mice were significant. This data provides both qualitative and quantitative evidence that the livers of Polβ^L301R-V303R/L301R-V303R^ HOM mice have significantly lower levels of Polβ protein as compared to their WT counterparts. After we observed significantly diminished Polβ protein abundance, we set out to explore the molecular mechanism behind these decreased protein levels. Given that two mutations were introduced to the Polβ gene, we examined whether the Polβ gene was being expressed at a lower level in the HOM mice. We conducted qRT-PCR and measured the fold change in mRNA expression of the Polβ and XRCC1 genes. Interestingly, there was no significant difference in the Polβ or XRCC1 mRNA expression levels in the livers of WT, HET, and HOM mice (Figure 1C).

**Figure 2:**
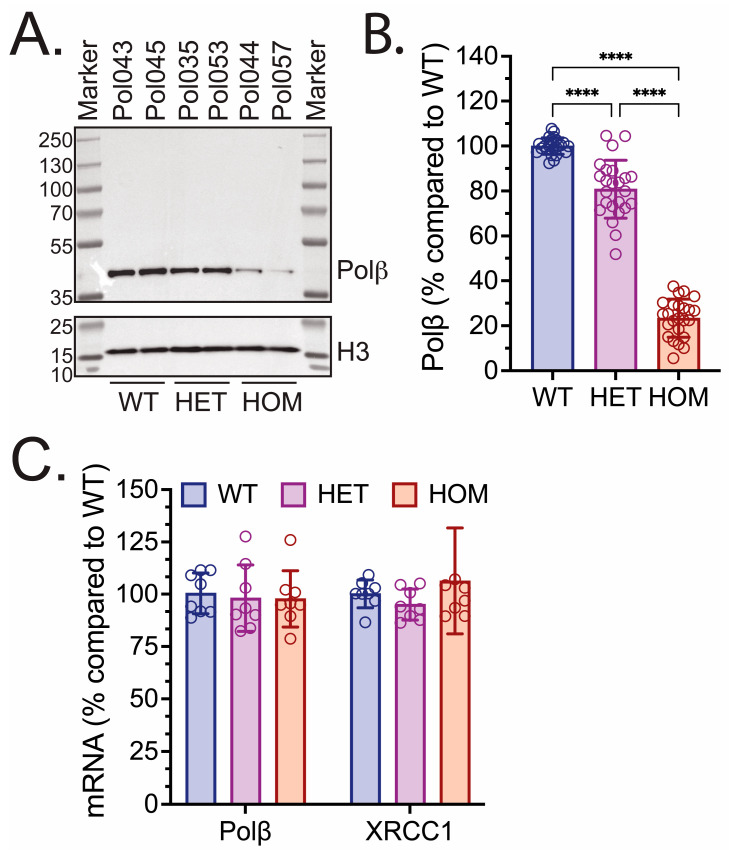
Polβ protein and mRNA levels in brains of WT, HET, and HOM Polβ^L301R-V303R/L301R-V303R^ KI mice. (**A**) Immunoblot of brain (left hemisphere) protein lysates from WT, HET, and HOM mice (Mouse Cohort #1), probing for Polβ. Sample names above each lane indicate the mouse identification number, with the genotype listed below. A representative immunoblot image is shown, which includes protein lysates from the brains of two mice of each genotype. Histone H3 was used as a loading control. (**B**) Protein level of Polβ in the brains of WT, HET, and HOM mice (Mouse Cohort #1). Protein level is shown as a % compared to WT. Quantifications are from immunoblot analysis, as in panel (**A**) and Supplementary Figure S2. Each bar represents 24 values (8 mice and 3 technical replicates per mouse), and each data point represents an independent immunoblot band. Values presented are mean ± SD. Significant differences are indicated by asterisks (**** *p* < 0.0001). (**C**) mRNA expression of Polβ and Xrcc1 isolated from brain tissue (left hemisphere) of WT, HET, and HOM mice (Mouse Cohort #1). Results represent % changes in gene expression relative to WT, calculated using the ΔΔCT method, with β-actin as an endogenous control. Each bar represents the mean ± SD of eight mice, and each data point represents the average of the samples run in quadruplicate.

We then probed for Polβ protein levels in brain samples isolated from WT, HET, and HOM mice (Mouse Cohort #1). Samples from the left hemisphere of the brain were isolated from each mouse, disrupted and homogenized, and processed to yield brain protein lysates. Immunoblot analysis of mouse brain protein lysates showed that Polβ protein levels were also reduced in the brains of Polβ^L301R-V303R/L301R-V303R^ mice (Figure 2A, two rightmost samples; Figure S2). Polβ protein levels were 23.4% in the HOM mouse brains, and 80.8% in HET mouse brains, as compared to WT littermates (Figure 2B). All differences between groups were significant. Similarly, there was no significant difference in Polβ or XRCC1 mRNA expression levels between WT, HET, and HOM mice in the brain (Figure 2C).

**Figure 3:**
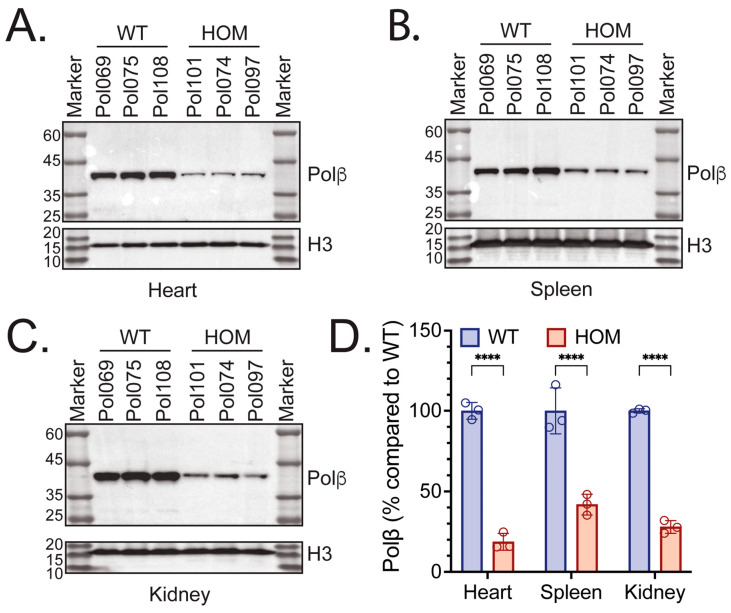
Polβ protein levels in hearts, spleens, and kidneys of WT, HET, and Polβ^L301R-V303R/L301R-V303R^ KI mice. Immunoblots of heart (**A**), spleen (**B**), and kidney (**C**) protein lysates from WT and Polβ^L301R-V303R/L301R-V303R^ mice (Mouse Cohort #2), probing for Polβ. Sample names above each lane consist of genotype and mouse identification number, with the tissue type indicated below. In each immunoblot, protein lysates of the appropriate tissue from 3 WT and 3 HOM mice were used (Mouse Cohort #2). Histone H3 was used as a loading control. (**D**) Protein level of Polβ in the hearts, spleens, and kidneys of WT and HOM mice (Mouse Cohort #2). Protein level is shown as a % compared to WT. Quantifications are from immunoblot analysis, as in panels (**A**–**C**). Each bar represents the mean ± SD of three mice. Significant differences are indicated by asterisks (**** *p* < 0.0001).

Following analysis of the Polβ protein levels in the livers and brains of WT, HET, and HOM mice, we aimed to provide a more exhaustive characterization of the Polβ protein levels by probing heart, spleen, and kidney samples from WT and HOM mice (Mouse Cohort #2). Each was then homogenized and processed to yield protein lysates. Immunoblot analysis of mouse heart, spleen, and kidney protein lysates showed that Polβ protein levels were visibly reduced in all three organs in HOM mice, reflecting our findings in the liver and brain (Figure 3A, Figure 3B and Figure 3C, respectively). Polβ protein levels were 18.7%, 41.8%, and 27.9% in the hearts, spleens, and kidneys of HOM mice, respectively, as compared to WT littermates, all of which were significantly different (Figure 3D).

There were no significant gross or microscopic pathologic changes observed in any of the mice in any tissue examined. When the WT and HOM groups were compared, again, there were no significant differences in pathologic changes by group or sex. Of the 12 mice, the blood from one female HOM mouse clotted and was unavailable for Complete Blood Count (CBC) analysis. Of the 11 mice with CBC analysis, the values fell within the standard deviation for C57BL/6 mice (Jax). There were no significant differences by sex or genotype between WT and HOM mice. However, consistent with our previous report, the HOM mice presented with slightly lower body weight for age and sex (Figure S3) [72].

### 3.2. Polβ Protein Levels Across Brain Sections

**Figure 4:**
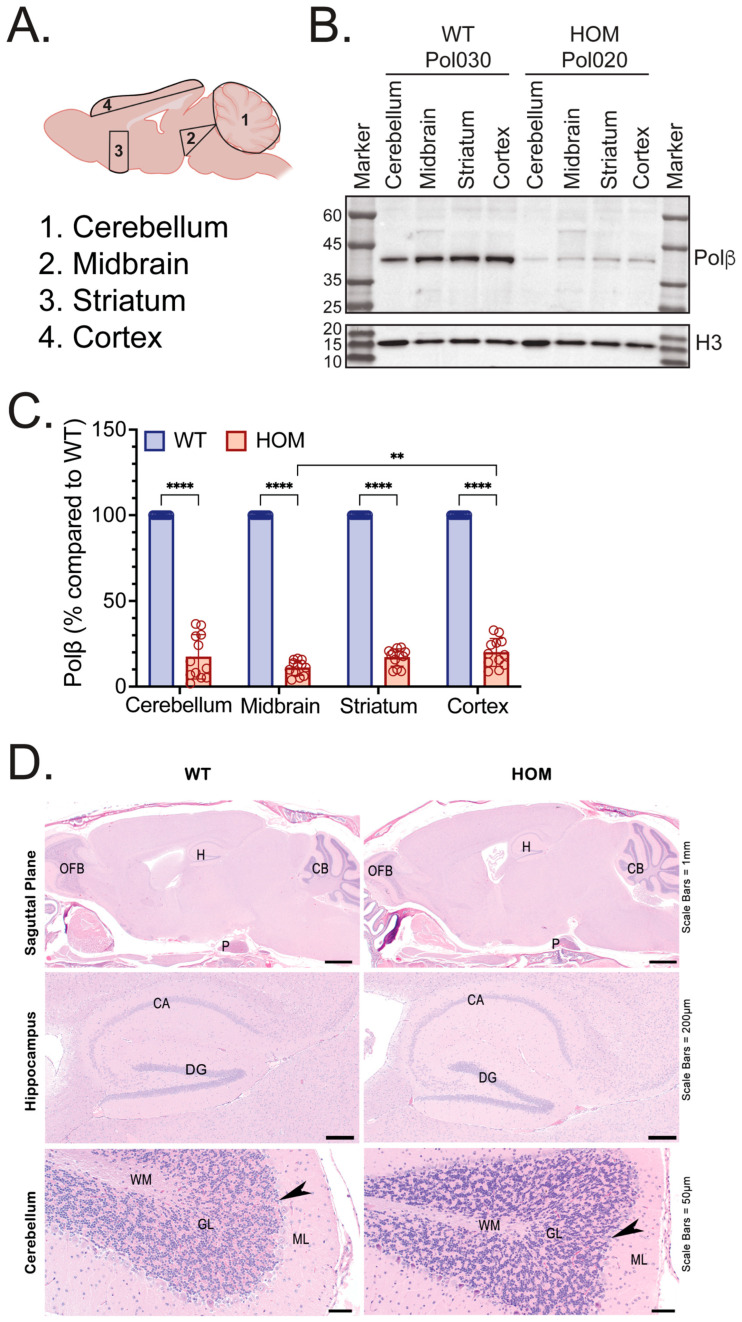
Polβ protein levels in brain regions of WT and HOM Polβ^L301R-V303R/L301R-V303R^ KI mice. (**A**) Graphic depicting the brain regions isolated from mice for protein and mRNA analysis. Created in BioRender. Sobol, R. (2026) https://BioRender.com/p1qoxrb. (**B**) Immunoblot of protein lysates from the cerebellum, midbrain, striatum, and cortex micro-dissected from WT and HOM mice (Mouse Cohort #3), probing for Polβ. Sample names above indicate the mouse identification number, genotype, and the brain section isolated. A representative immunoblot image is shown, which includes protein lysates from the brain regions of one mouse of each genotype. Histone H3 was used as a loading control. (**C**) Protein level of Polβ in the cerebellum, midbrain, striatum, and cortex of WT (blue) and HOM (red) mice (Mouse Cohort #3). Protein level is shown as a % compared to WT. Quantifications are from immunoblot analysis, as in panel (**B**) and Supplementary Figure S4. Each bar represents 12 values (4 mice and 3 technical replicates per mouse). Values presented are mean ± SD. Significant differences are indicated by asterisks (** *p* < 0.01, **** *p* < 0.0001). (**D**) Representative low and higher-power images of midline sagittal brain H&E sections from WT and HOM mice (Mouse Cohort #2). There is no microscopic difference in the olfactory bulb (OFB), hippocampus (H), pituitary (P), and cerebellum (CB) between the WT and HOM mice. CA = cornu ammonis, DG = dentate gyrus, WM = white matter, GL = granular layer, ML = molecular layer, and the layer of Purkinje cells (arrowheads). Scale bar for Saguttal Plane = 1 mm, for Hippocampus = 200 µm, and for Cerebellum = 50 µm.

To evaluate the extent of diminished Polβ protein expression in older Polβ^L301R-V303R/L301R-V303R^ mouse brains, four brain regions (cerebellum, midbrain, striatum, and cortex) were isolated via micro-dissection from eight (4 WT and 4 HOM) mice between 20 and 24 months of age (see Figure 4A for a map of micro-dissected brain regions) (Mouse Cohort #3). Tissue samples were disrupted, homogenized, and processed to yield protein lysates. Immunoblot analysis of mouse brain region samples demonstrated that Polβ protein levels were visibly diminished in the cerebellum, midbrain, striatum, and cortex of older HOM mice as compared to older WT mice (Figure 4B and Figure S4). This result is consistent with our findings in the brain, liver, heart, spleen, and kidney (Mouse Cohorts #1 and #2). Quantification of the immunoblots using ImageJ revealed that Polβ protein levels in the HOM cerebellum, midbrain, striatum, and cortex were 17.4%, 11.0%, 17.0%, and 20.0% of WT littermates, respectively (Figure 4C). All differences between Polβ protein levels in WT and HOM mice in the cerebellum, midbrain, striatum, and cortex were significant. Polβ protein levels were also significantly lower in the HOM midbrain as compared to the HOM cortex (Figure 4C). As above, we found no significant gross or microscopic pathologic changes observed in the brain from Mouse Cohort #2 (Figure 4D).

### 3.3. Polβ Stability in L301R/V303R HOM Mouse Ear Fibroblasts

Since Polβ gene expression was unaffected in Polβ^L301R-V303R/L301R-V303R^ HOM mice, we considered another potential explanation for the diminished Polβ protein abundance. Previous studies with human cells and immortalized mouse fibroblasts have reported that the Polβ/XRCC1 interaction is crucial for maintaining Polβ protein stability [72,74]. In this way, Polβ that is unable to interact with XRCC1 is marked for ubiquitylation and degraded [74], predominantly by TRIP12 [75] and is also degraded by ubiquitin-independent processes [110]. Since the L301R and V303R mutations have been shown to block Polβ’s ability to interact with XRCC1 [72], we then set out to examine whether the diminished Polβ protein abundance in Polβ^L301R-V303R/L301R-V303R^ HOM mice could be due to Polβ protein instability and subsequent degradation by the proteasome. WT and Polβ^L301R-V303R/L301R-V303R^ HOM mouse ear fibroblast cells were treated for 6 h with the protein synthesis inhibitor cycloheximide (cyclo) and/or the proteasome inhibitor MG132.

**Figure 5:**
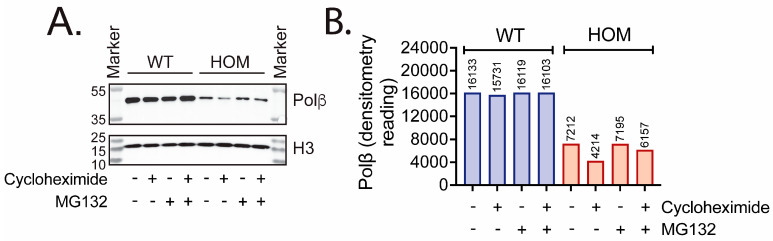
Polβ stability in Polβ^L301R-V303R/L301R-V303R^ mouse ear fibroblast cells treated with cycloheximide and MG132. (**A**) Immunoblot of whole cell lysates from WT and Polβ^L301R-V303R/L301R-V303R^ mouse-derived ear fibroblast cells treated with a protein synthesis inhibitor (cycloheximide), a proteasome inhibitor (MG132), or a combination of both. Histone H3 was used as a loading control. (**B**) Quantification of the immunoblot depicted in (**A**), showing relative Polβ protein levels. Values above the bars represent densitometric quantifications obtained via ImageJ, accounting for H3 loading differences.

As expected, lower Polβ levels (about 44.7% as compared to WT cells) were observed in untreated HOM cells (Figure 5A). Treatment of HOM cells with cycloheximide resulted in a visible decrease in Polβ levels, about 58.4% as compared to untreated HOM cells (Figure 5B). Treatment of WT cells with MG132 or MG132+cycloheximide did not have a noticeable effect on Polβ protein levels as compared to untreated WT cells. In HOM cells, treatment with MG132 only or MG132+cycloheximide combined did not recover Polβ to near-WT levels. However, quantification of the blot revealed that in HOM cells, treatment with MG132 and MG132+cycloheximide resulted in approximately a 70.7% and 46.1% increase in Polβ levels, respectively, as compared to HOM cells treated with cycloheximide only (Figure 5B). Further, HOM cells treated with MG132 and MG132+cycloheximide exhibited 99.8% and 85.4% Polβ protein levels as compared to untreated HOM cells.

### 3.4. Genome Skimming of WT, HET, and HOM Polβ KI Mice Reveals Tissue-Specific Methylation Levels

**Figure 6:**
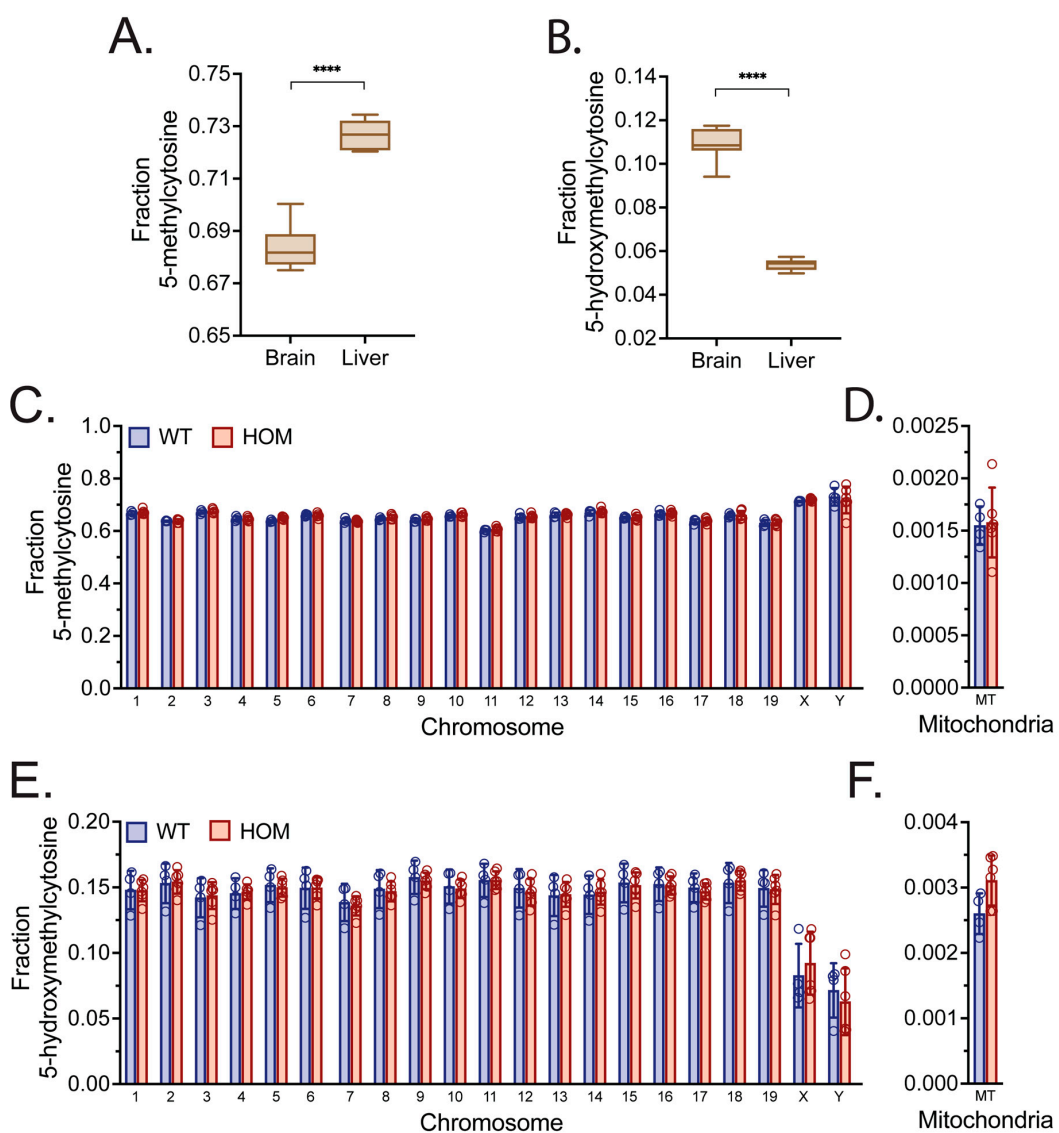
Global genomic and mitochondrial DNA methylation analysis isolated from the midbrain of WT and HOM, Polβ^L301R-V303R/L301R-V303R^ mice. The global genomic fraction of (**A**) 5mC and (**B**) 5hmC in the brain and liver isolated from Mouse Cohort #1 as measured by genome skimming with nanopore sequencing [109]. Statistical analysis was performed to detect significance between liver and brain samples (**A**,**B**). Values presented are mean ± SD. Significant differences are indicated by asterisks (**** *p* < 0.0001). The fraction of 5mC (**C**,**D**), or 5hmC (**E**,**F**), in each chromosome from nuclear genomic DNA (**C**,**E**), or from mitochondrial DNA (**D**,**F**), isolated from the midbrain of WT, and HOM mice (Mouse Cohort #3) as measured by genome skimming with nanopore sequencing [109]. Statistical analysis for panels (**C**–**F**) was performed to detect significance between genotypes, all indicating n.s.

Previous studies suggested that BER and Polβ may be critical to neuronal development and the maintenance of a healthy neuronal genome specifically by modulating active gene demethylation, and that a deficiency in Polβ may lead to abnormal methylation patterns in the brain and exacerbate neurodegeneration [46,91,102,111]. Therefore, we conducted genome skimming via nanopore sequencing of DNA samples isolated from the liver and brains of WT, and HOM mice (Mouse Cohort #1) to examine global methylation levels. The percentage (%) of 5-methylcytosine (5mC) levels was significantly higher in mouse livers (~72%) than brains (~68%) (Figure 6A). The opposite trend was observed for % 5hmC (5-hydroxymethylcytosine) levels, with significantly higher % 5hmC in brains (~10%) than in livers (5%) (Figure 6B), as expected [112]. We next conducted a more in-depth analysis of the DNA samples from the midbrains of older mice (Mouse Cohort #3), as this region was found to have the lowest level of Polβ expression of the tissues evaluated (Figure 4C). We quantified the level of 5mC and 5hmC in the DNA isolated from the midbrain samples, evaluating at each chromosome and the mitochondria (Figure 6C–F). However, no significant differences were detected in % 5mC or % 5hmC levels between the WT or HOM mice when evaluated at the resolution possible via genome skimming.

## 4. Discussion

We recently reported the generation of the Polβ^L301R-V303R/L301R-V303R^ KI mouse, which, unlike Polβ-KO mouse models, was found to be viable, fertile, and apparently healthy, yet exhibited a modest decrease in body weight [72,76,78]. As a result, the Polβ-XRCC1 interaction does not seem to be required for mouse viability or fertility [72]. Fibroblasts derived from the ears of the HOM KI mice exhibited 15% Polβ protein abundance as compared to WT cells, implicating the role of the Polβ-XRCC1 interface in regulating Polβ levels and/or stability, as we found in human cells where the stability of Polβ, when not bound to XRCC1, is regulated by TRIP12-mediated ubiquitylation [75], as well as by ubiquitin-independent processes [110]. However, a full characterization of the levels of the protein, RNA, and DNA of this mouse was necessary to elucidate the significance of the Polβ-XRCC1 interaction in vivo.

In this study, we have demonstrated that Polβ^L301R-V303R/L301R-V303R^ HOM KI mice have significantly diminished Polβ protein abundance across various tissues, including the brain, liver, heart, spleen, and kidney (Figure 1, Figure 2, Figure 3 and Figure 4). These findings are consistent with studies in human cells [74] and mouse fibroblasts [72] with identical mutations to residues essential to the Polβ-XRCC1 interface. Relative Polβ protein levels in HOM mice varied between tissue types: the heart and brain exhibited the most severe decreases in Polβ, while the spleen exhibited the least severe decrease. These results are consistent with past reports suggesting that Polβ has tissue-specific functions [76,113,114].

We also showed that Polβ mRNA expression levels were unaffected by the L301R/V303R mutations (Figure 1C and Figure 2C). This is in agreement with a past report in human glioblastoma Polβ(L301R/V303R/V306R) triple mutant (TM) cells, which demonstrated diminished Polβ abundance despite identical mRNA levels to WT cells [74] and that Polβ is targeted for ubiquitylation by TRIP12 [75]. This study, along with a study in colon cancer cells, point to the hypothesis that the Polβ-XRCC1 interaction is essential for mediating Polβ stability; Polβ that is not bound to XRCC1 is ubiquitylated at K206 and K244 and marked for proteasome-mediated degradation [74,75,110]. These findings suggest that the diminished Polβ levels in vivo reported in this study may be a product of proteasome-mediated degradation [75]. The hypothesis that the unstable Polβ mutant, in HOM mice, is degraded by the proteasome was consistent with the sensitivity of HOM Polβ in mouse ear fibroblasts to a protein synthesis inhibitor and subsequent recovery using a proteasome inhibitor (Figure 5).

Given the mounting evidence that the Polβ-XRCC1 interaction is not essential for the DNA damage response and instead mediates Polβ stability, our finding that treatment of Polβ^L301R-V303R/L301R-V303R^ mouse ear fibroblasts with a protein synthesis inhibitor and proteasome inhibitor result in only a minor recovery of Polβ protein levels is somewhat surprising. Nearly identical experiments in previous studies have utilized human cell lines [74,75,110], suggesting that the treatment times utilized in these studies (≤6 h) may not be directly translatable to mouse ear fibroblasts or as was shown in human cancer cells, some of the instability may be due to ubiquitin-independent processes [110].

The 5mC and 5hmC DNA methylation results obtained from WT, and HOM KI mice also indicate that more in-depth analysis is required in the future. The distinct levels of % 5mC and % 5hmC observed in the livers and brains of Cohort #1 mice is consistent with differences observed in the human genome, with lower proportions of 5mC levels and higher proportions of 5hmC in the brain (and vice versa in the liver) [115,116]. Further, the average depth of coverage between all samples from Cohort #1 was 0.07X, with a minimum of 0.01X and a maximum of 0.18X, which falls in the coverage range necessary to obtain accurate results using the genome skimming with nanopore sequencing strategy [109]. Polβ deficiency was associated with increased DSBs and aberrant gene demethylation in hippocampal pyramidal neurons [46]. However, we found no evidence for differences in global methylation levels or methylation patterns in the livers and brains of HOM mice (Cohort #1). We also found no evidence for differences in methylation levels or patterns in the midbrains of older HOM mice (Cohort #3). Future studies would however benefit from a more detailed analysis of DNA methylation patterns, ideally at single-base resolution.

Previously, it has been shown that BER defects can impact age-related neurodegeneration [10,88,117], such as was found for the 3xTg/Polβ^+/−^ mouse model [102] that presented with neuronal dysfunction. Further, given the role of Polβ in the response to various genotoxins [43,118,119], its role in regulating replication-stress mediated poly(ADP-ribose) signaling [120,121,122], and its role in mitochondrial DNA repair [123,124], it will be interesting to continue analysis of this Polβ hypomorph mouse model with regard to dietary stress such as folate deficiency [125,126,127], to environmental genotoxic stress including alkylating agents such N-Nitroso-dimethylamine (NDMA) [128], as well as to age-related oxidative stress seen in Parkinson’s disease [129,130,131,132].

## 5. Conclusions

Overall, this study provides substantial evidence that Polβ^L301R-V303R/L301R-V303R^ mice have significantly diminished Polβ protein levels in a range of tissues with unchanged mRNA levels, pointing to a decrease in Polβ protein stability. We hypothesize that the impaired ability of Polβ to interact with XRCC1 is causing protein destabilization, resulting in degradation of Polβ mediated, at least in part, by the proteasome in vivo. Despite the lack of a change in basal methylation levels in the Polβ^L301R-V303R/L301R-V303R^ mice, the diminished Polβ protein levels and potential Polβ instability suggest that this mouse model may be helpful for investigating the role of Polβ in aberrant active gene demethylation or genome maintenance in response to environmental genotoxins, alterations in diet, dietary supplements, or due to dietary stress, and/or when evaluated in both young and aged animals.

## Data Availability

The original contributions presented in this study are included in the article/Supplementary Material. Further inquiries can be directed to the corresponding author.

## Supplementary Materials

The following supporting information can be downloaded at: https://www.mdpi.com/article/10.3390/biom16030412/s1, Table S1: Reagent List; Figure S1: Immunoblots from liver samples isolated from Cohort #1; Figure S2: Immunoblots from brain samples isolated from Cohort #1; Figure S3: Body weight analysis from Cohort #2; Figure S4: Immunoblots from micro-dissected brain regions isolated from Cohort #3.
