# Supplementary material for: Hypomorphic Protein Expression of DNA Polymerase Beta in PolβL301R-V303R/L301R-V303R Knock-In Transgenic Mice Does Not Impact Global DNA Methylation Levels in the Midbrain
Source: Biomolecules. 2026 Mar 11;16(3):412. doi: 10.3390/biom16030412 (PMC13023549; doi:10.3390/biom16030412)

Supplemental Material for:

Hypomorphic protein expression of DNA polymerase beta in Polβ$^{L301R\text{-}V303R/L301R\text{-}V303R}$ knock-in transgenic mice does not impact global DNA methylation levels in the midbrain

Bryce Jacobs[1], Dan Ivanov[1], Ivana Brazza[2,3], Christopher Faulk[4], Carmen J. Booth[5], Raquel Mattos-Canedo[1], Lucas Tian[1], Kaitlyn DePietro[1], Alper Uzun[1], Wynand P. Roos[1], Laurie H. Sanders[2,3], and Robert W. Sobol[1]*

[1]Department of Pathology and Laboratory Medicine, Warren Alpert Medical School & Legorreta Cancer Center, Brown University, Providence, RI 02912
[2]Departments of Neurology and Pathology, Duke University, Durham, NC 27708

[3]Duke Center for Neurodegeneration and Neurotherapeutics, Duke University, Durham, NC, 27710, USA

[4]Department of Animal Sciences, University of Minnesota, Minneapolis, MN 55455

[5]Department of Comparative Medicine, Yale University School of Medicine, New Haven CT 06520

Running title: Suppressed expression of Polβ in mice

Keywords: DNA polymerase beta, demethylation, base excision repair, Xrcc1

***Address correspondence to**:
Robert W. Sobol, Ph.D.
Department of Pathology and Laboratory Medicine
Warrant Alpert Medical School & Legorreta Cancer Center
Brown University, Providence, RI 02912
rwsobol@brown.edu

Supplemental Data include Supplemental Table S1 and Figures S1-S4.

**Table S1: Reagent List**	**Source**	**Identifier**
**Antibodies**		
Rabbit anti-Polβ (Immunoblot 1:2000)	Abcam	Cat# ab175197
Rabbit anti-H3 (Immunoblot 1:2000)	Cell Signaling Technology	Cat# 4499S
Rabbit anti-α-actinin (Immunoblot 1:2000)	Cell Signaling Technology	Cat# 6487S
Goat Anti-Rabbit IgG (H + L)-HRP Conjugate (Immunoblot 1:5000)	Bio-Rad	Cat# 1706515
**Chemicals, peptides, and materials**		
DNeasy Blood & Tissue Kit	Qiagen	Cat# 69504
Allprotect Tissue Reagent	Qiagen	Cat# 76405
Allprep DNA/RNA/Protein Mini Kit	Qiagen	Cat# 80004
qRT-PCR H_2O	Invitrogen	Cat# AM9935
NuPAGE 4-12% Bis-Tris	Invitrogen	Cat# NP0321BOX
OneTaq 2X Master Mix with Standard Buffer	New England Biolabs	Cat# M0486S
XcmI Restriction Endonuclease	New England Biolabs	Cat# R0533S
NEBuffer Restriction Digest Buffer	New England Biolabs	Cat# B6002S
10% neutral buffered formalin	Lab Chem	Cat# LC146702
4X Laemmli sample buffer	Bio Rad	Cat# 1610747
0.2µm nitrocellulose membranes	Bio Rad	Cat# 1704270
Tris Buffered Saline	Bio Rad	Cat# 1706435
10% Tween 20 Solution	Bio Rad	Cat# 1610781
Blotting grade non-fat dry milk	Bio Rad	Cat# 1706404
PCR primers	Integrated DNA Technologies	Custom

2-Mercaptoethanol	Sigma	Cat# M6250
TBE Buffer (10X)	Thermo Scientific	Cat# B52
SeaKem LE Agarose	Lonza	Cat# 50004
5X Transfer Buffer	Bio Rad	Cat# 10026938
Clarity Western ECL Substrate	Bio Rad	Cat# 1705061
SuperSignal West Femto Luminol/Enhancer	Thermo Scientific	Cat# 1856189
SuperSignal West Femto Stable Peroxide	Thermo Scientific	Cat# 1856190
SuperScript VILO cDNA Synthesis Kit	Invitrogen	Cat# 11754050
Applied Biosystems TaqMan Gene Expression Assays Polβ	Fisher Scientific	Cat# POLB MM00448234_M1
Applied Biosystems TaqMan Gene Expression Assays XRCC1	Fisher Scientific	Cat# XRCC1 MM00494222_M1
Applied Biosystems TaqMan Gene Expression Assays β-Actin	Fisher Scientific	Cat# BETA ACTIN MN02619580_G1
Cycloheximide	Sigma Aldrich	Cat# C7698-1G
MG132	Selleck Chemicals	Cat# S2619
Penicillin/streptomycin	Thermo Fisher Scientific	Cat# 15140-122
RPMI 1640	Corning	Cat# 10-016-CV
Antibiotic/antimycotic	Thermo Fisher Scientific	Cat# 15240-062
Heat-inactivated fetal bovine serum	Bio-Techne	Cat# S11150H
Tissue-Tek Optimal Cutting Temperature compound	Sakura	Cat# 4583
Magnesium chloride hexahydrate	Sigma-Aldrich	Cat# M2393
Sucrose sugar	Sigma-Aldrich	Cat# S0389

16% paraformaldehyde	Electron Microscopy Sciences	Cat# 15710-250ML
**Software**		
ImageJ	Image J 1.48v	https://imagej.nih.gov/ij/ 1.6.0_65
Adobe Illustrator	Adobe Systems	Version 29.3
NIS-Elements	Nikon Instruments	Versions 4.51 and 5.11
Dorado	Version 0.8.0	https://github.com/nanoporetech/dorado/
Modkit	Version 0.4.1	https://github.com/nanoporetech/modkit

Figure S1: Immunoblots from liver samples isolated from Cohort #1.

Immunoblot of liver protein isolates from WT, HET, and HOM mice (Cohort #1), probing for Polβ. H3 was used as a loading control. Sample names above each lane indicate the mouse identification number, with the genotype listed after each number. These full blots represent all samples used to develop the quantitative analysis shown in **Figure 1B**.

Supplementary Figure S1
6 to 9-month Liver samples
Mouse Cohort #1
Immunoblots

Hypomorphic protein expression of DNA polymerase beta in Polβ$^{L301R\text{-}V303R/L301R\text{-}V303R}$ knock-in transgenic mice does not impact global DNA methylation levels in the midbrain

Bryce Jacobs[1], Dan Ivanov[1], Ivana Brazza[2,3], Chris Faulk[4], Carmen J. Booth[5], Raquel Mattos-Canedo[1], Lucas Tian[1], Kaitlyn DePietro[1], Alper Uzun[1], Wynand P. Roos[1], Laurie H. Sanders[2,3], and Robert W. Sobol[1]*

Loaded 30ug
Antibodies used:
1:1000 Anti-Polβ (Abcam, Cat# ab175197)
1:2000 Anti-H3 (Cell Signaling, Cat# 4499S)

WT: Pol050 x Pol051
HET: Pol049 x Pol052
HOM: Pol047 x Pol061
Run #1: 8/8/24

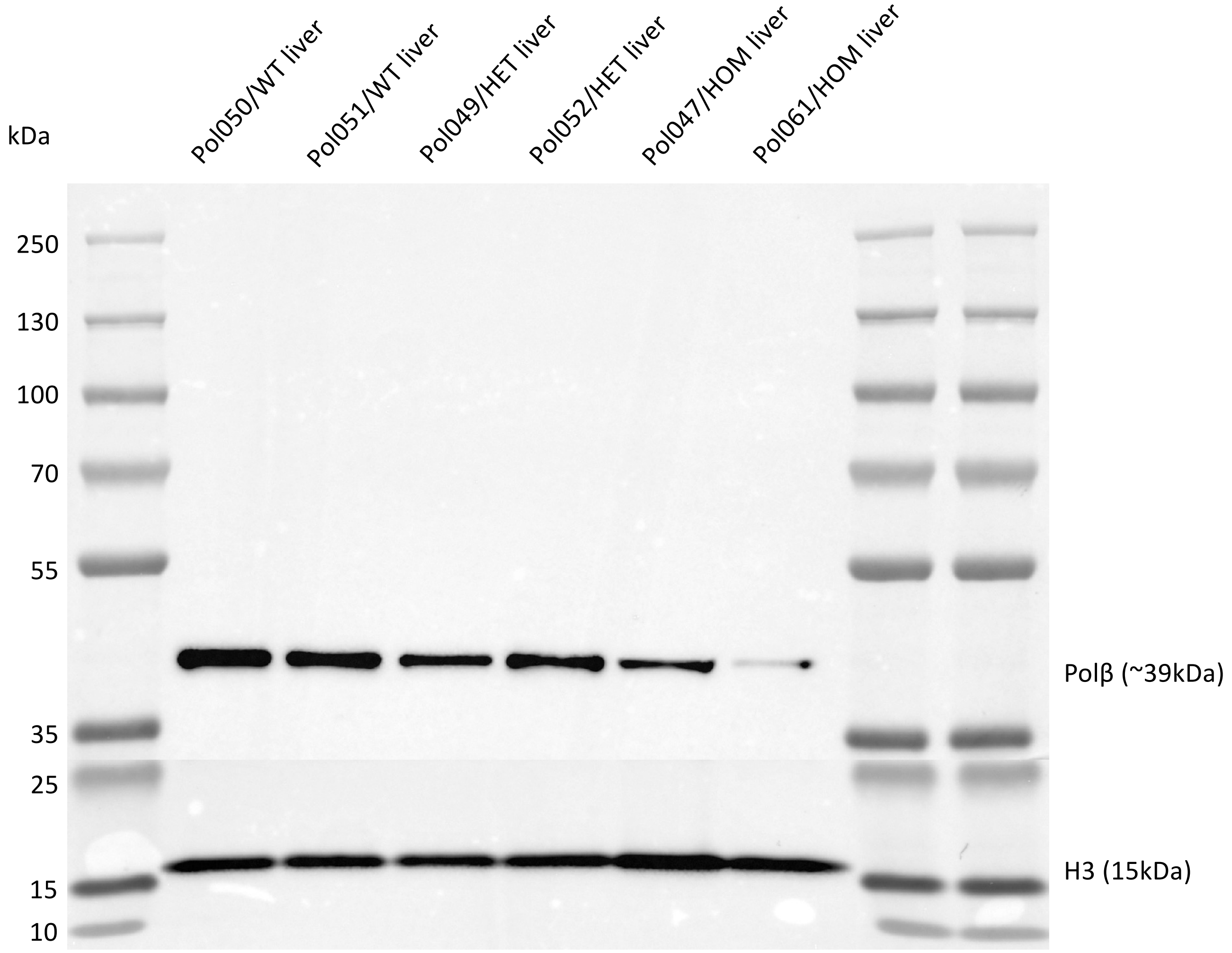

Loaded 30ug

Antibodies used:

1:1000 Anti-Polβ (Abcam, Cat# ab175197)

1:2000 Anti-H3 (Cell Signaling, Cat# 4499S)

WT: Pol050 x Pol051

HET: Pol049 x Pol052

HOM: Pol047 x Pol061

Run #2: 9/15/24

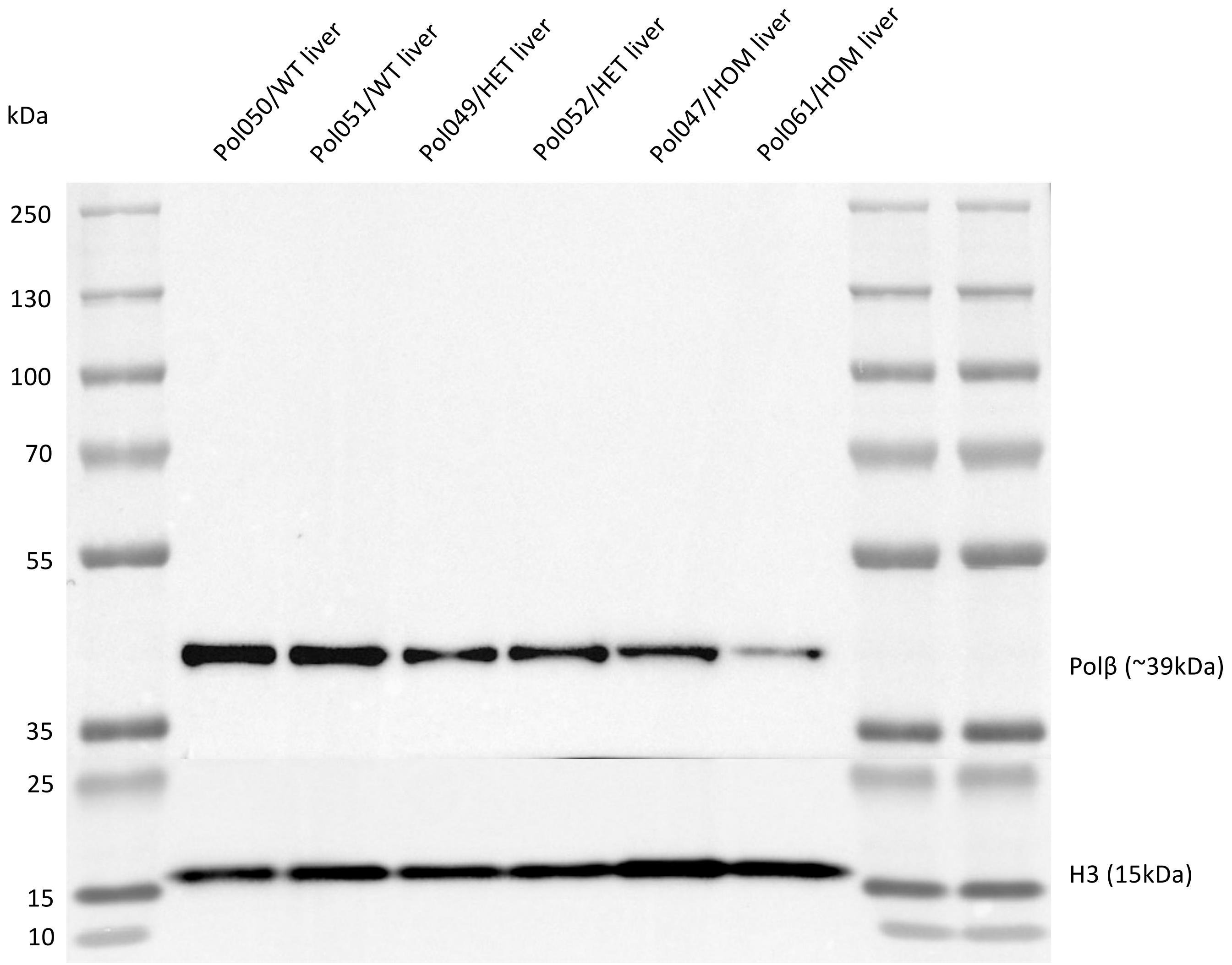

Loaded 30ug
Antibodies used:
1:1000 Anti-Polβ (Abcam, Cat# ab175197)
1:2000 Anti-H3 (Cell Signaling, Cat# 4499S)

WT: Pol050 x Pol051
HET: Pol049 x Pol052
HOM: Pol047 x Pol061
Run #3: 9/21/24

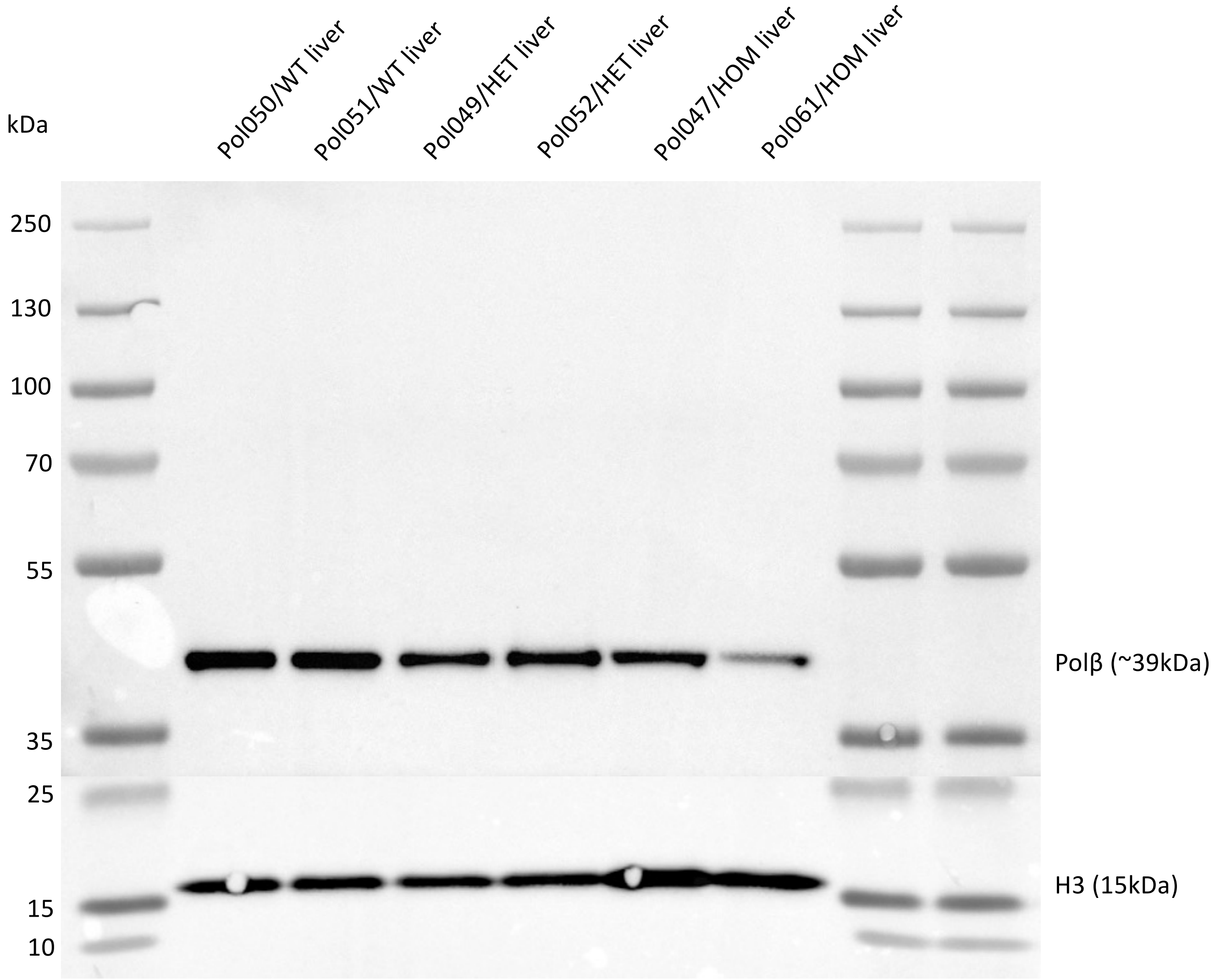

Loaded 30ug
Antibodies used:
1:1000 Anti-Polβ (Abcam, Cat# ab175197)
1:2000 Anti-H3 (Cell Signaling, Cat# 4499S)

WT: Pol043 x Pol045
HET: Pol035 x Pol053
HOM: Pol044 x Pol057
Run #1: 8/8/24

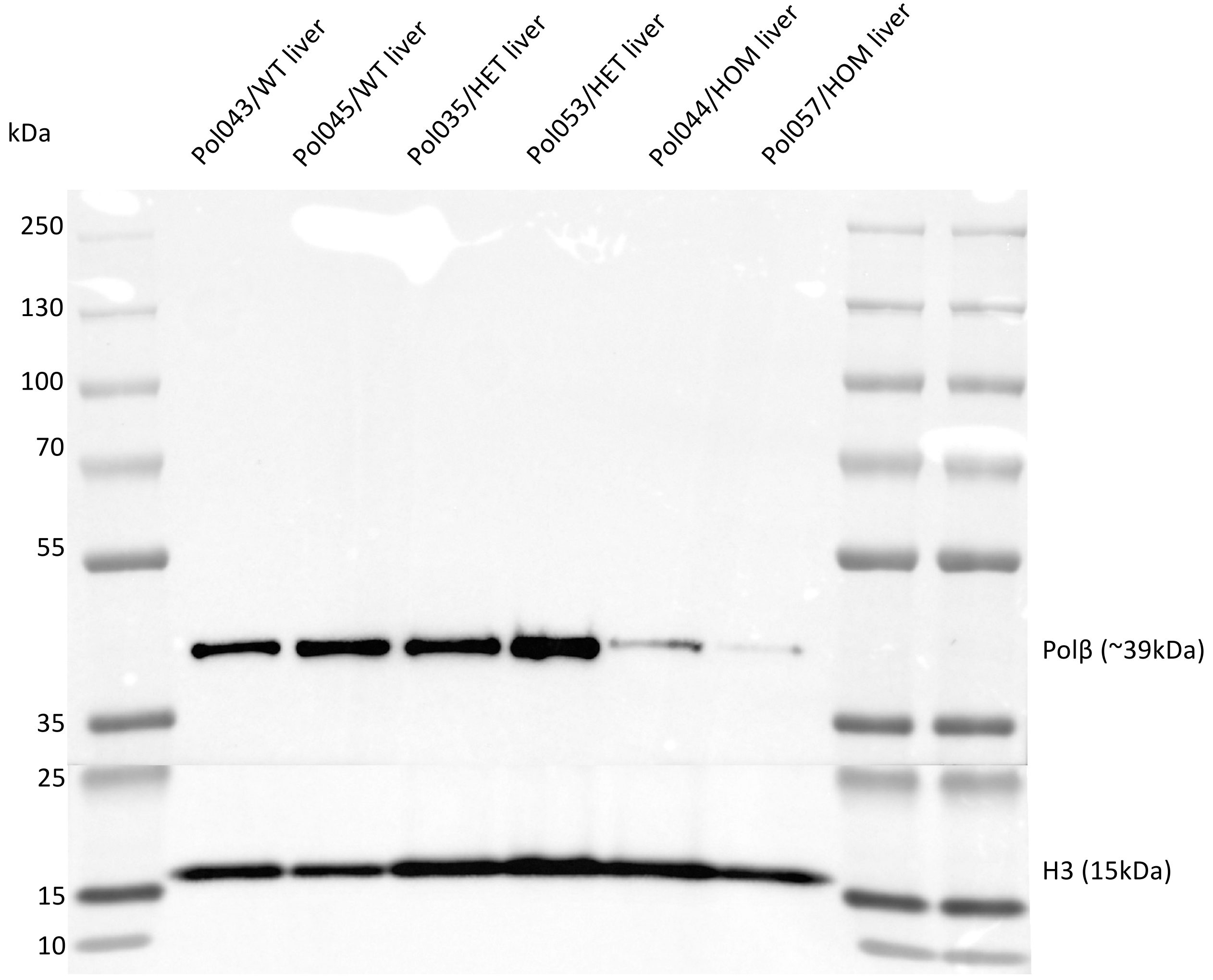

Loaded 30ug
Antibodies used:
1:1000 Anti-Polβ (Abcam, Cat# ab175197)
1:2000 Anti-H3 (Cell Signaling, Cat# 4499S)

WT: Pol043 x Pol045
HET: Pol035 x Pol053
HOM: Pol044 x Pol057
Run #2: 9/15/24

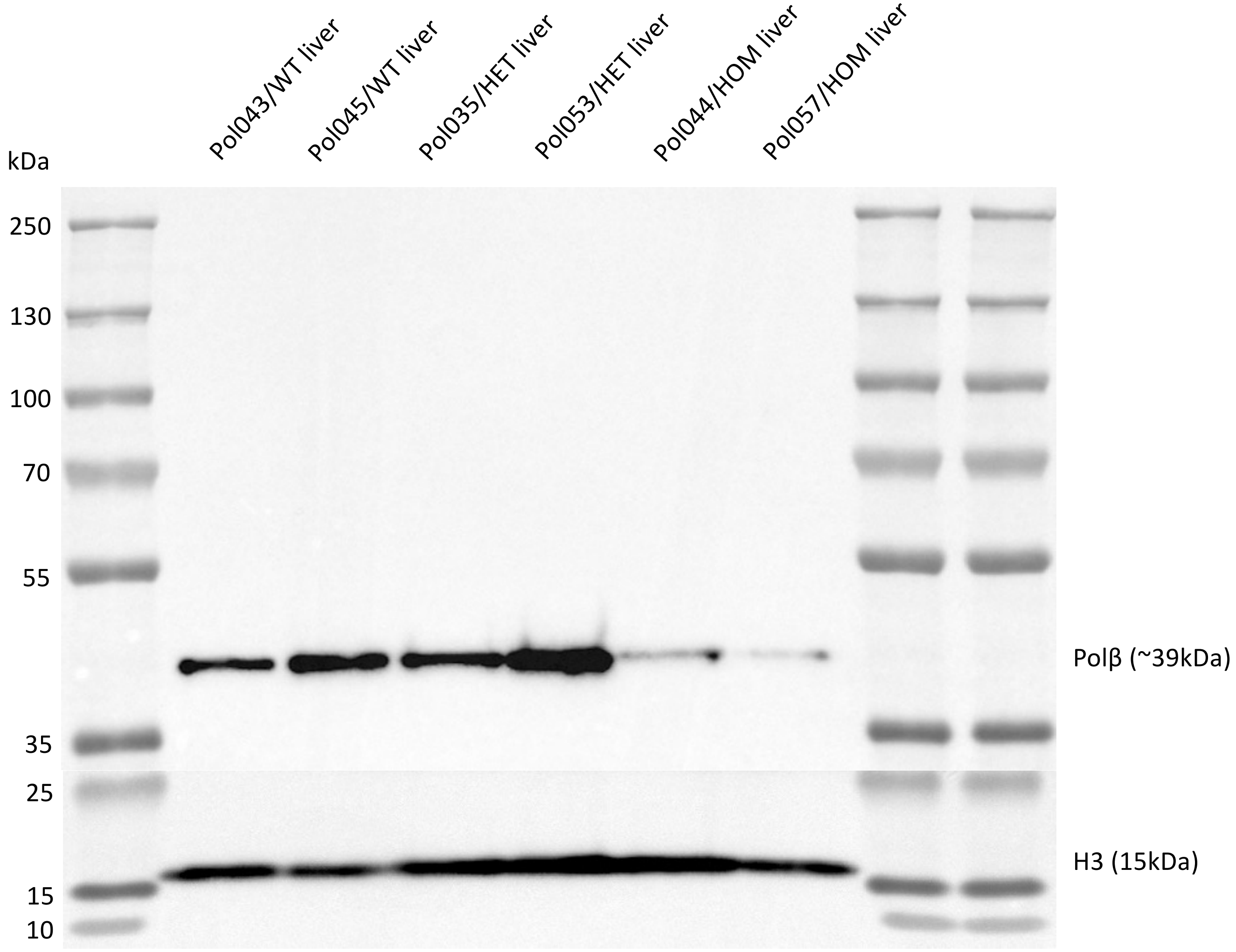

Loaded 30ug
Antibodies used:
1:1000 Anti-Polβ (Abcam, Cat# ab175197)
1:2000 Anti-H3 (Cell Signaling, Cat# 4499S)

WT: Pol043 x Pol045
HET: Pol035 x Pol053
HOM: Pol044 x Pol057
Run #3: 9/21/24

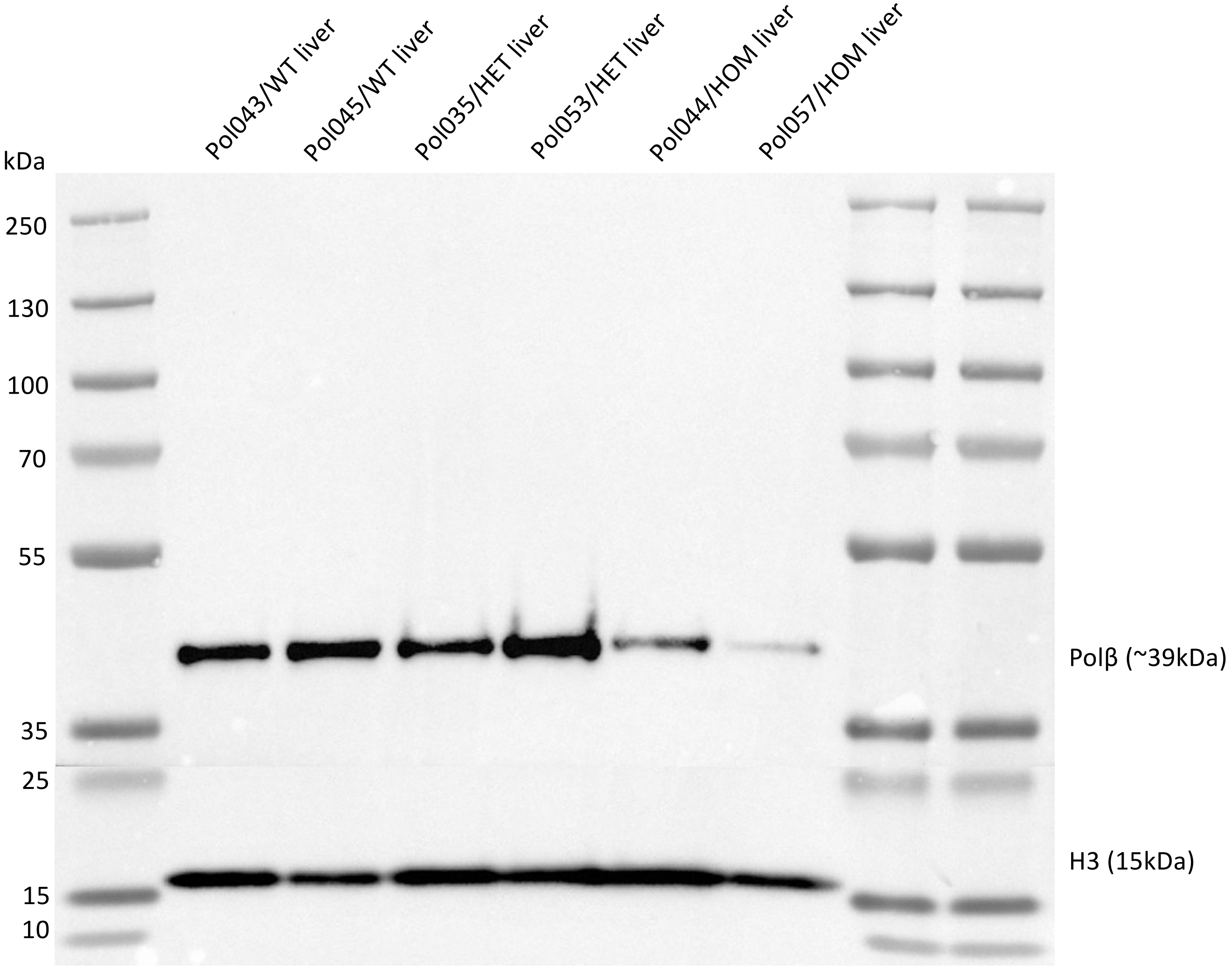

Loaded 30ug
Antibodies used:
1:1000 Anti-Polβ (Abcam, Cat# ab175197)
1:2000 Anti-H3 (Cell Signaling, Cat# 4499S)

WT: Pol105 x Pol116
HET: Pol113 x Pol115
HOM: Pol125 x Pol127
Run #1: 11/2/24

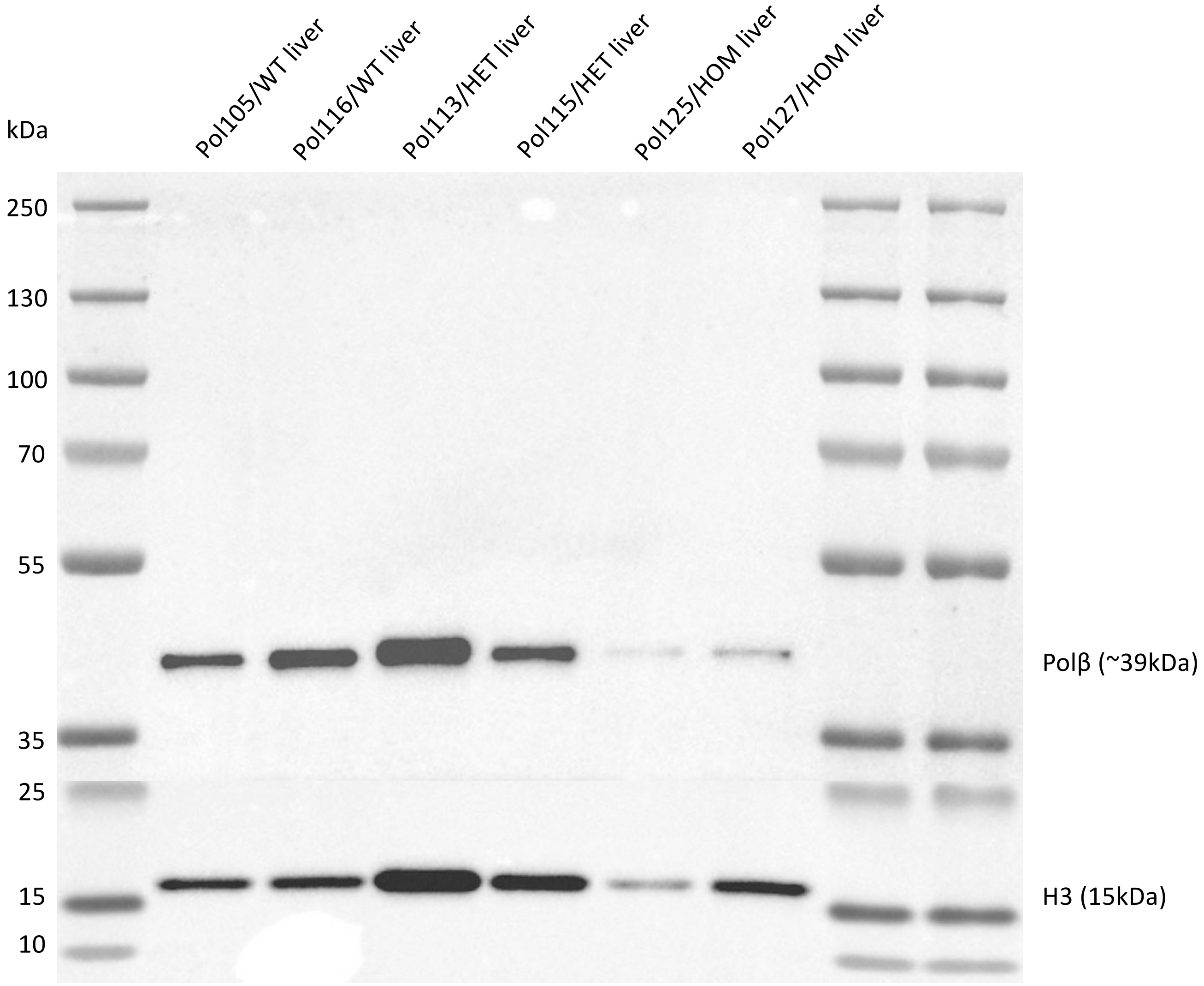

Loaded 30ug
Antibodies used:
1:1000 Anti-Polβ (Abcam, Cat# ab175197)
1:2000 Anti-H3 (Cell Signaling, Cat# 4499S)

WT: Pol105 x Pol116
HET: Pol113 x Pol115
HOM: Pol125 x Pol127
Run #2: 11/12/24

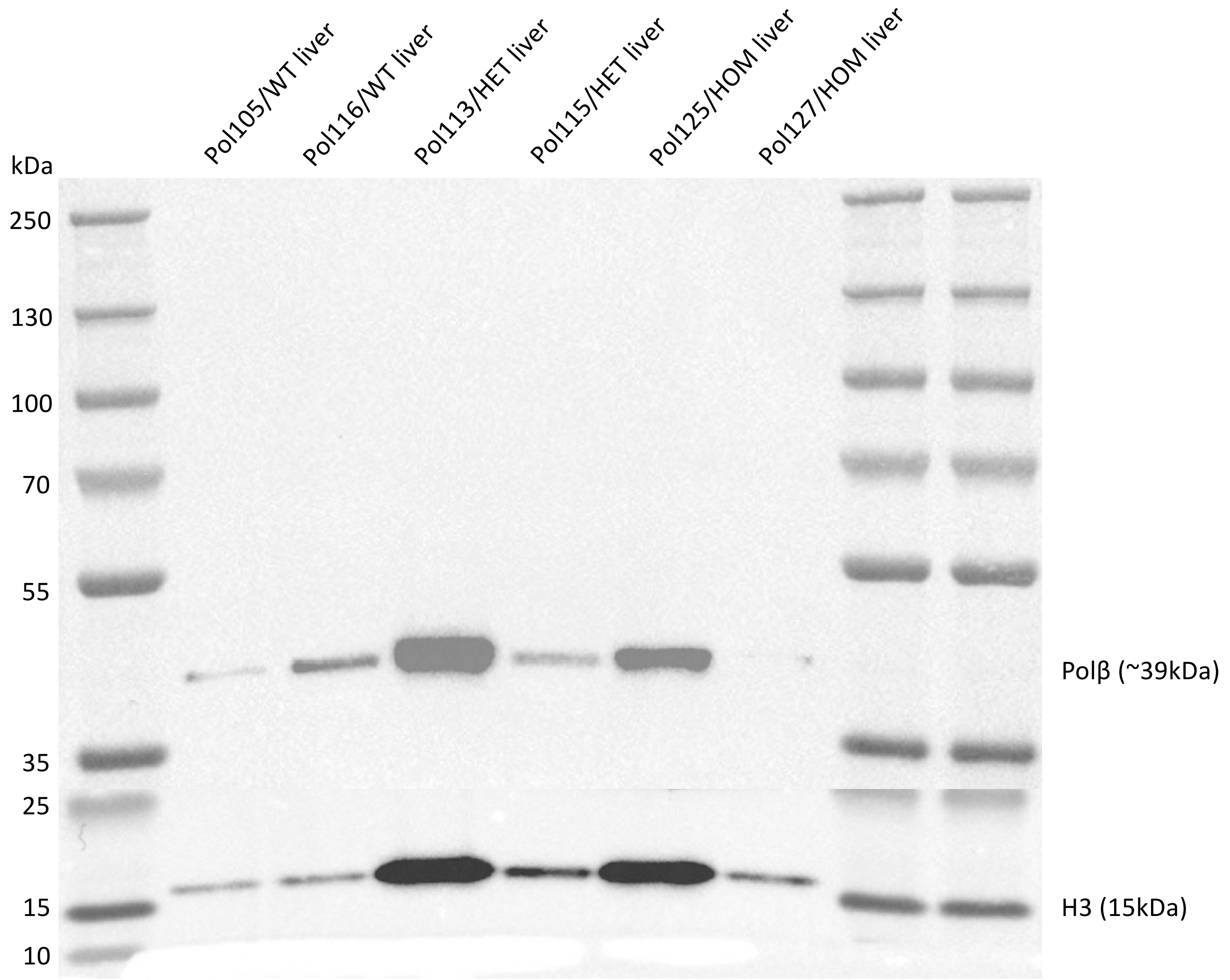

Loaded 30ug
Antibodies used:

1:1000 Anti-Polβ (Abcam, Cat# ab175197)
1:2000 Anti-H3 (Cell Signaling, Cat# 4499S)

WT: Pol105 x Pol116
HET: Pol113 x Pol115
HOM: Pol125 x Pol127
Run #3: 12/9/24

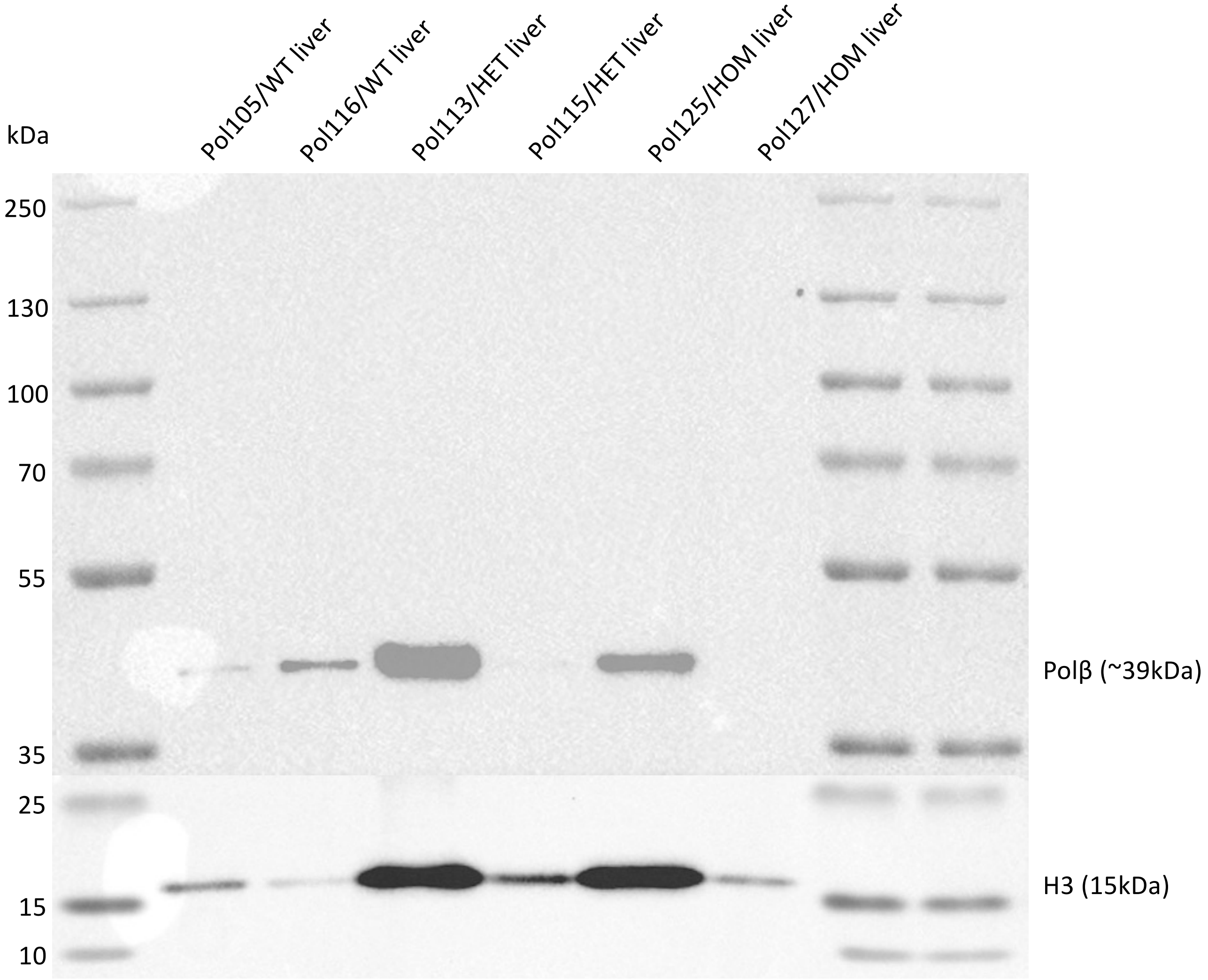

Loaded 30ug

Antibodies used:

1:1000 Anti-Polβ (Abcam, Cat# ab175197)

1:2000 Anti-H3 (Cell Signaling, Cat# 4499S)

WT: Pol118 x Pol122

HET: Pol117 x Pol119

HOM: Pol130 x Pol143

Run #1: 11/12/24

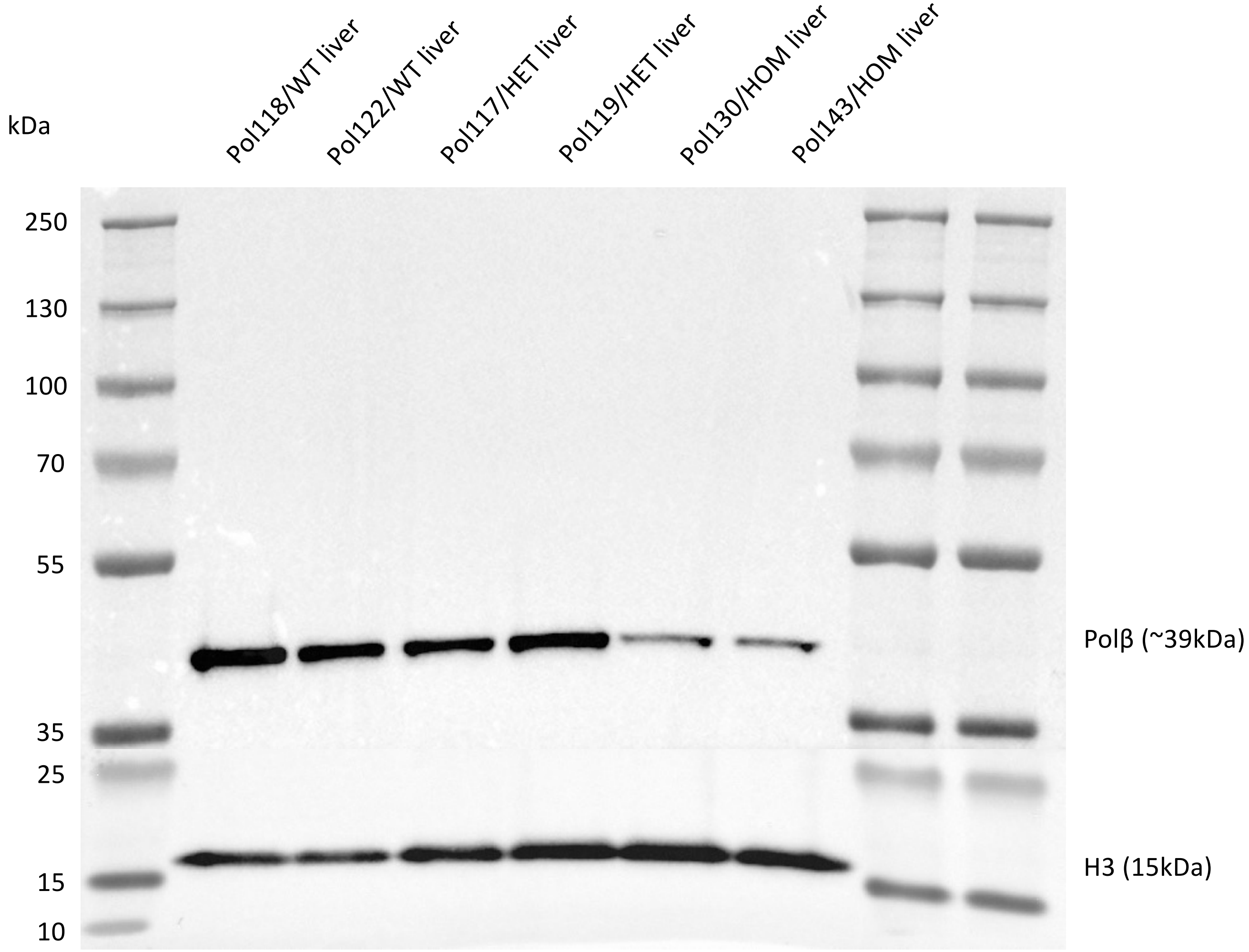

Loaded 30ug
Antibodies used:

1:1000 Anti-Polβ (Abcam, Cat# ab175197)
1:2000 Anti-H3 (Cell Signaling, Cat# 4499S)

WT: Pol118 x Pol122
HET: Pol117 x Pol119
HOM: Pol130 x Pol143
Run #2: 12/9/24

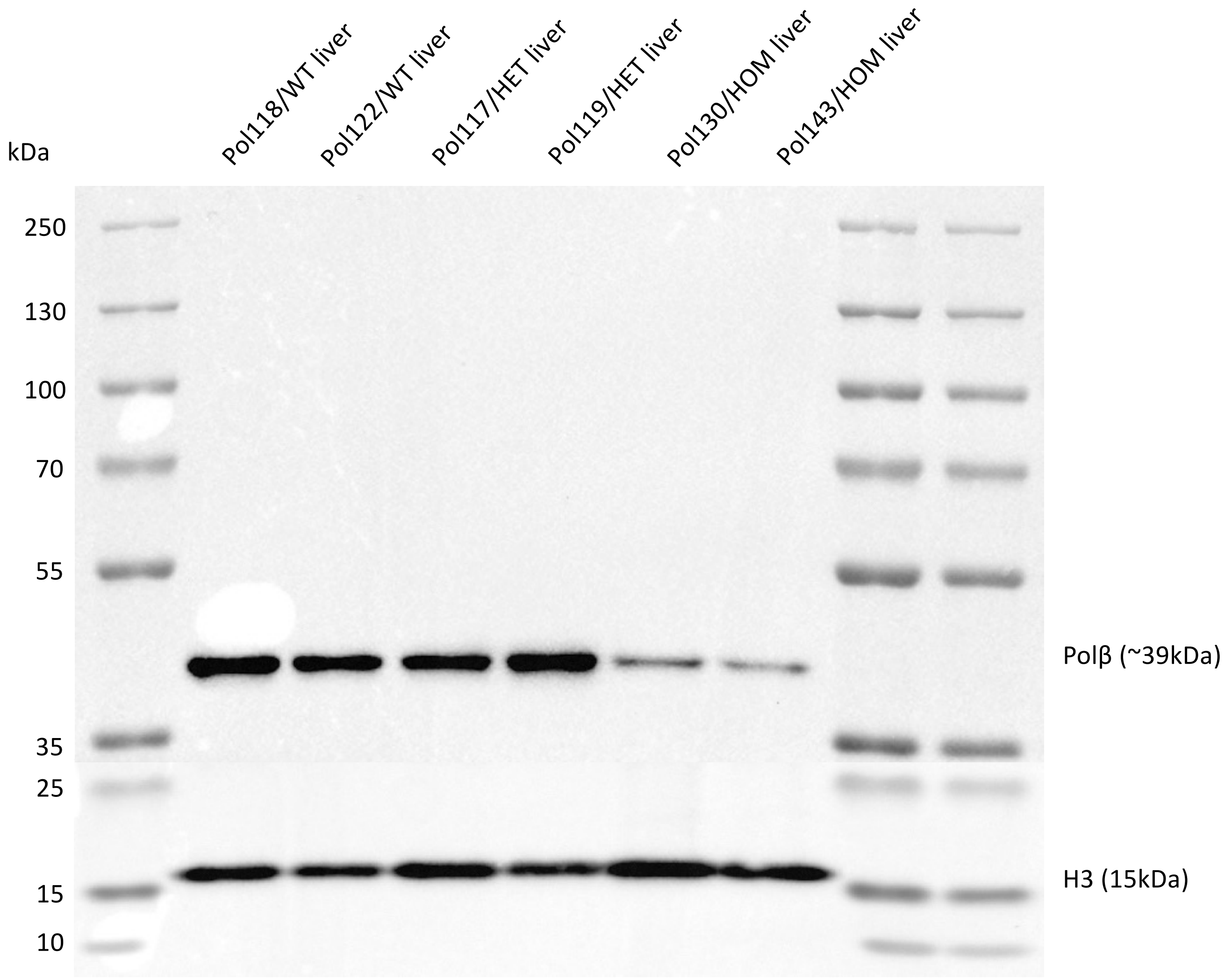

Loaded 30ug
Antibodies used:
1:1000 Anti-Polβ (Abcam, Cat# ab175197)
1:2000 Anti-H3 (Cell Signaling, Cat# 4499S)

WT: Pol118 x Pol122
HET: Pol117 x Pol119
HOM: Pol130 x Pol143
Run #3: 12/9/24

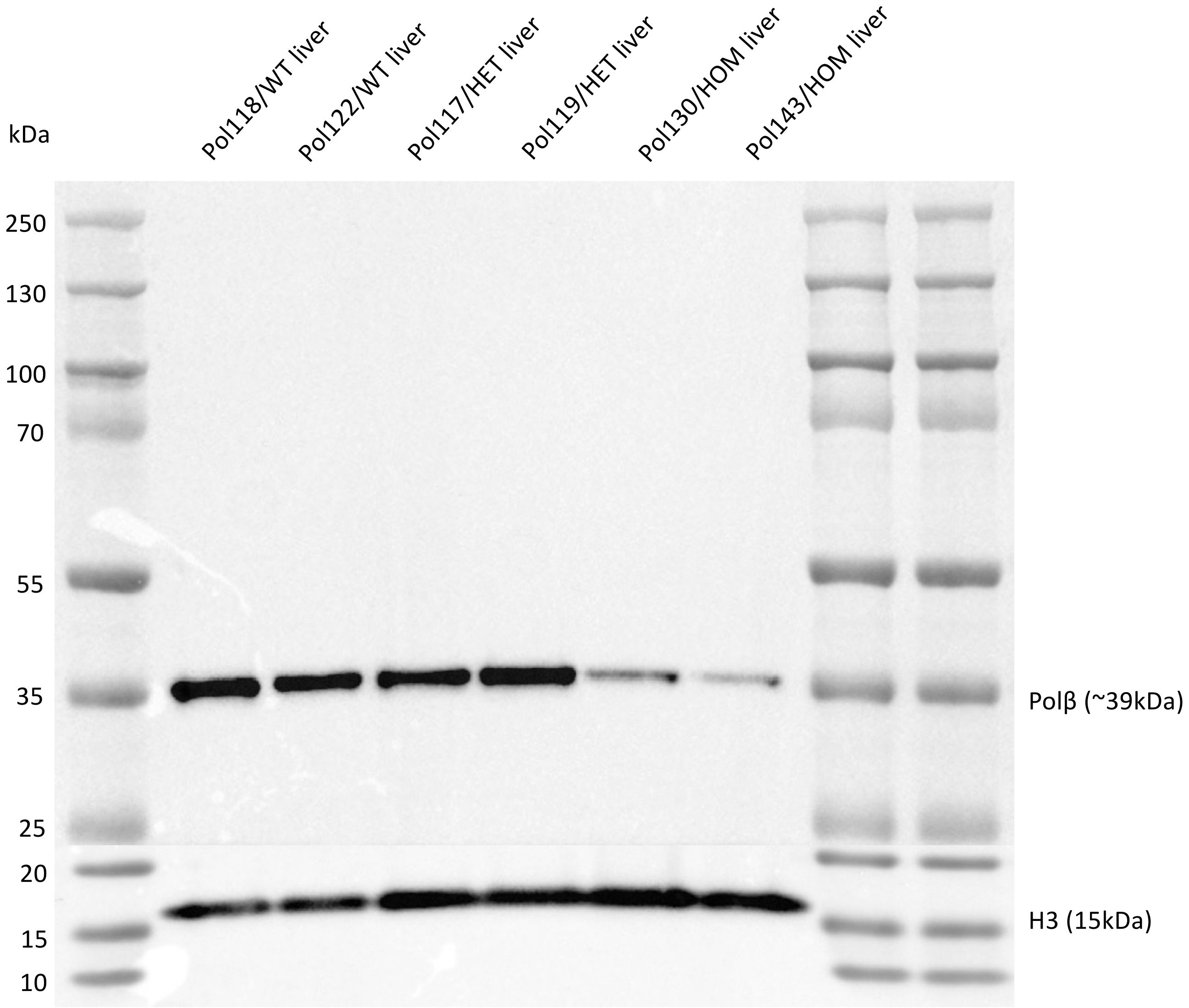

Figure S2: Immunoblots from brain samples isolated from Cohort #1.

Immunoblot of brain protein isolates from WT, HET, and HOM mice (Cohort #1), probing for Polβ. H3 was used as a loading control. Sample names above each lane indicate the mouse identification number, with the genotype listed after each number. These full blots represent all samples used to develop the quantitative analysis shown in **Figure 2B**.

Supplementary Figure S2
6 to 9-month Brain samples
Mouse Cohort #1
Immunoblots

Hypomorphic protein expression of DNA polymerase beta in Polβ$^{L301R\text{-}V303R/L301R\text{-}V303R}$ knock-in transgenic mice does not impact global DNA methylation levels in the midbrain

Bryce Jacobs[1], Dan Ivanov[1], Ivana Brazza[2,3], Chris Faulk[4], Carmen J. Booth[5], Raquel Mattos-Canedo[1], Lucas Tian[1], Kaitlyn DePietro[1], Alper Uzun[1], Wynand P. Roos[1], Laurie H. Sanders[2,3], and Robert W. Sobol[1]*****

Loaded 30ug
Antibodies used:
1:1000 Anti-Polβ (Abcam, Cat# ab175197)
1:2000 Anti-H3 (Cell Signaling, Cat# 4499S)

WT: Pol050 x Pol051
HET: Pol049 x Pol052
HOM: Pol047 x Pol061
Run #1: 8/8/24

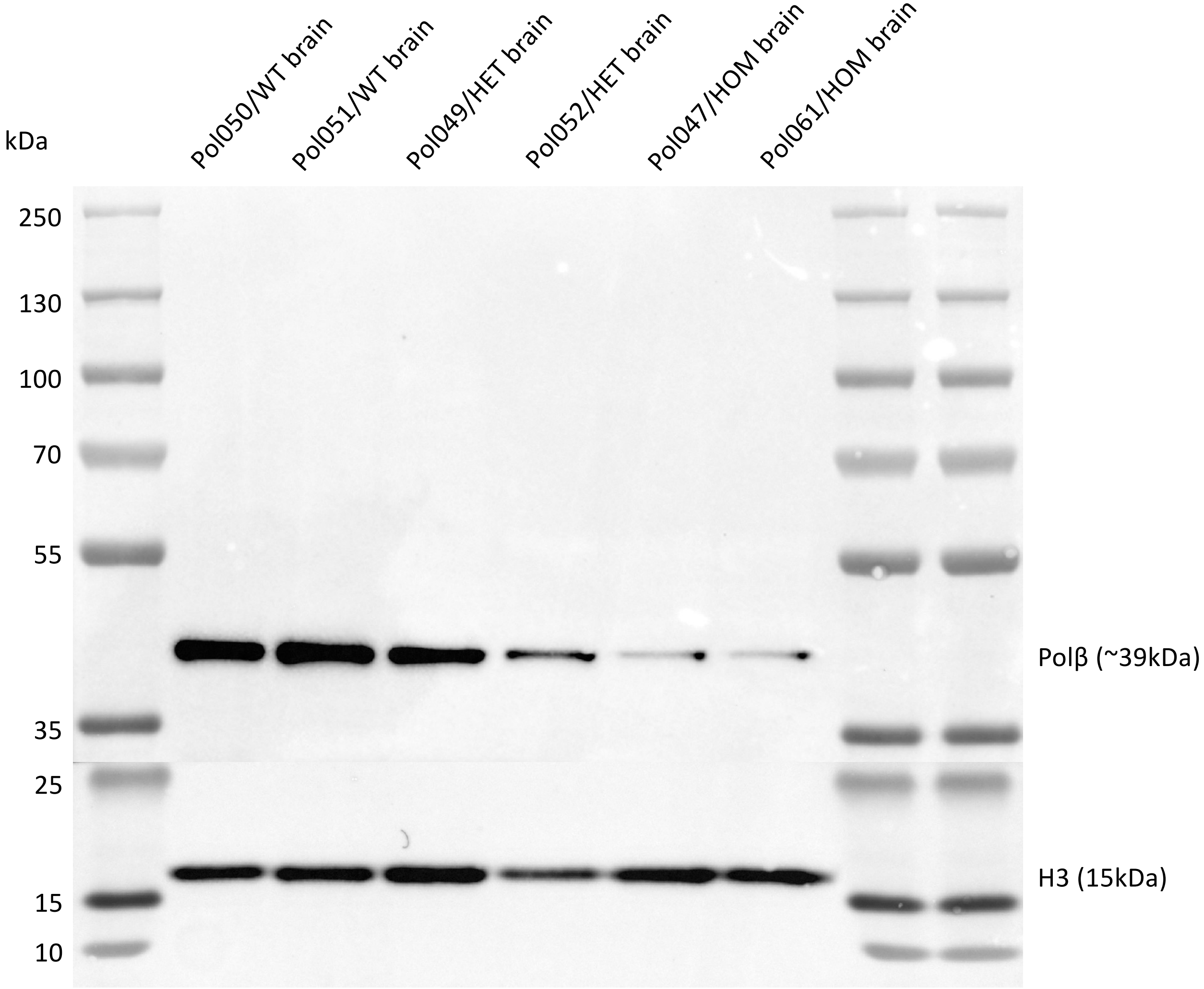

Loaded 30ug
Antibodies used:
1:1000 Anti-Polβ (Abcam, Cat# ab175197)
1:2000 Anti-H3 (Cell Signaling, Cat# 4499S)

WT: Pol050 x Pol051
HET: Pol049 x Pol052
HOM: Pol047 x Pol061
Run #2: 9/15/24

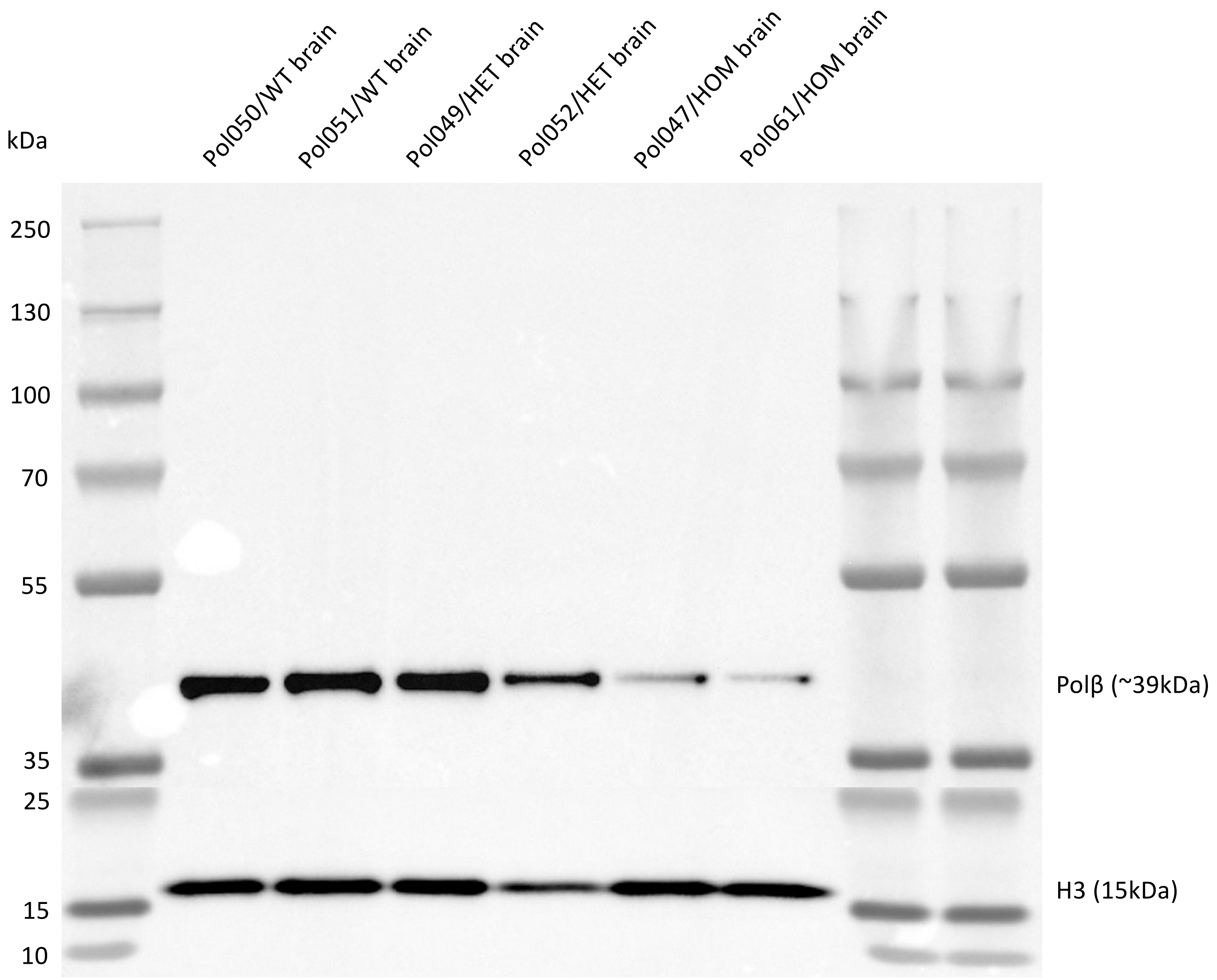

Loaded 30ug

Antibodies used:

1:1000 Anti-Polβ (Abcam, Cat# ab175197)

1:2000 Anti-H3 (Cell Signaling, Cat# 4499S)

WT: Pol050 x Pol051

HET: Pol049 x Pol052

HOM: Pol047 x Pol061

Run #3: 9/21/24

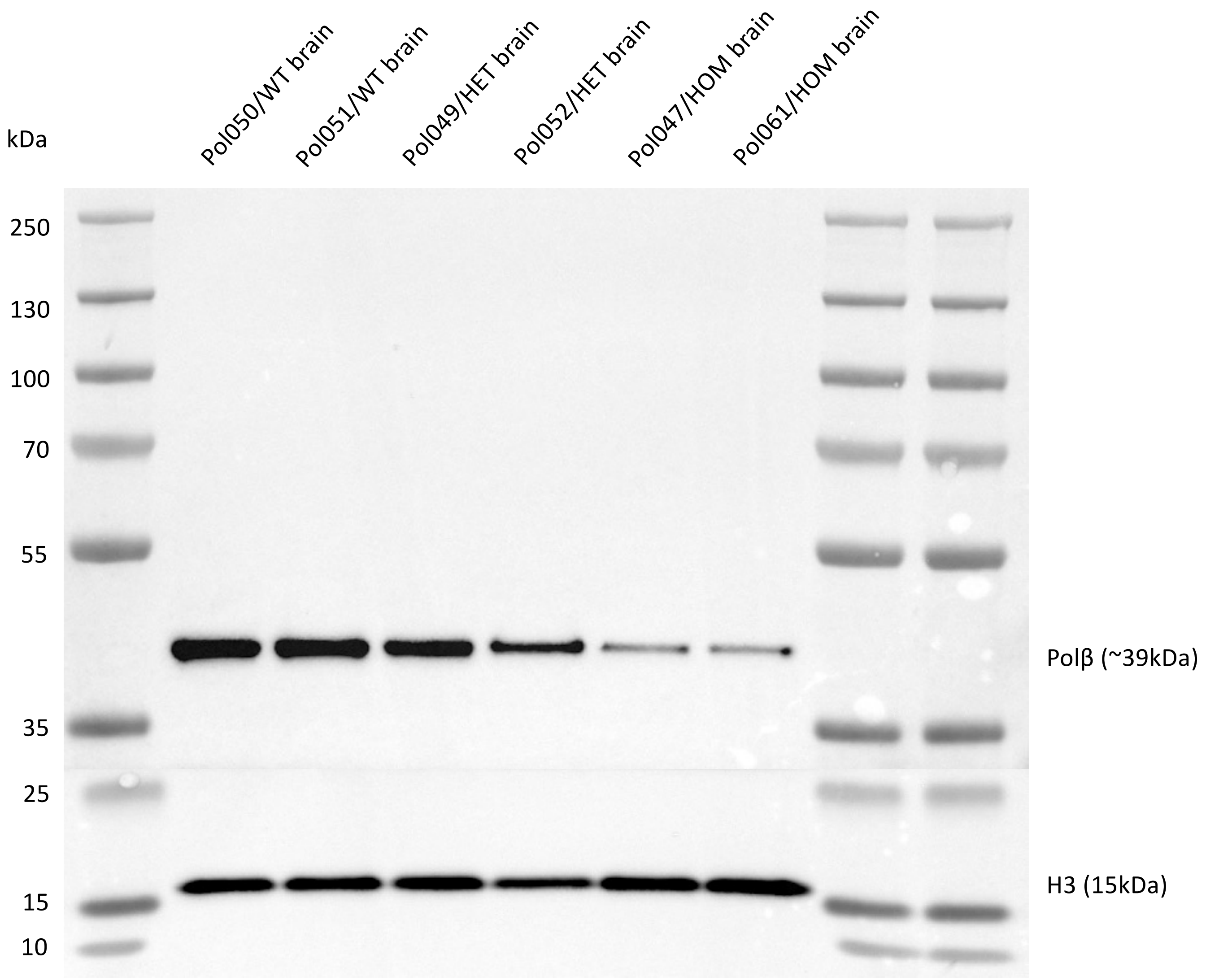

Loaded 30ug
Antibodies used:
1:1000 Anti-Polβ (Abcam, Cat# ab175197)
1:2000 Anti-H3 (Cell Signaling, Cat# 4499S)

WT: Pol043 x Pol045
HET: Pol035 x Pol053
HOM: Pol044 x Pol057
Run #1: 8/8/24

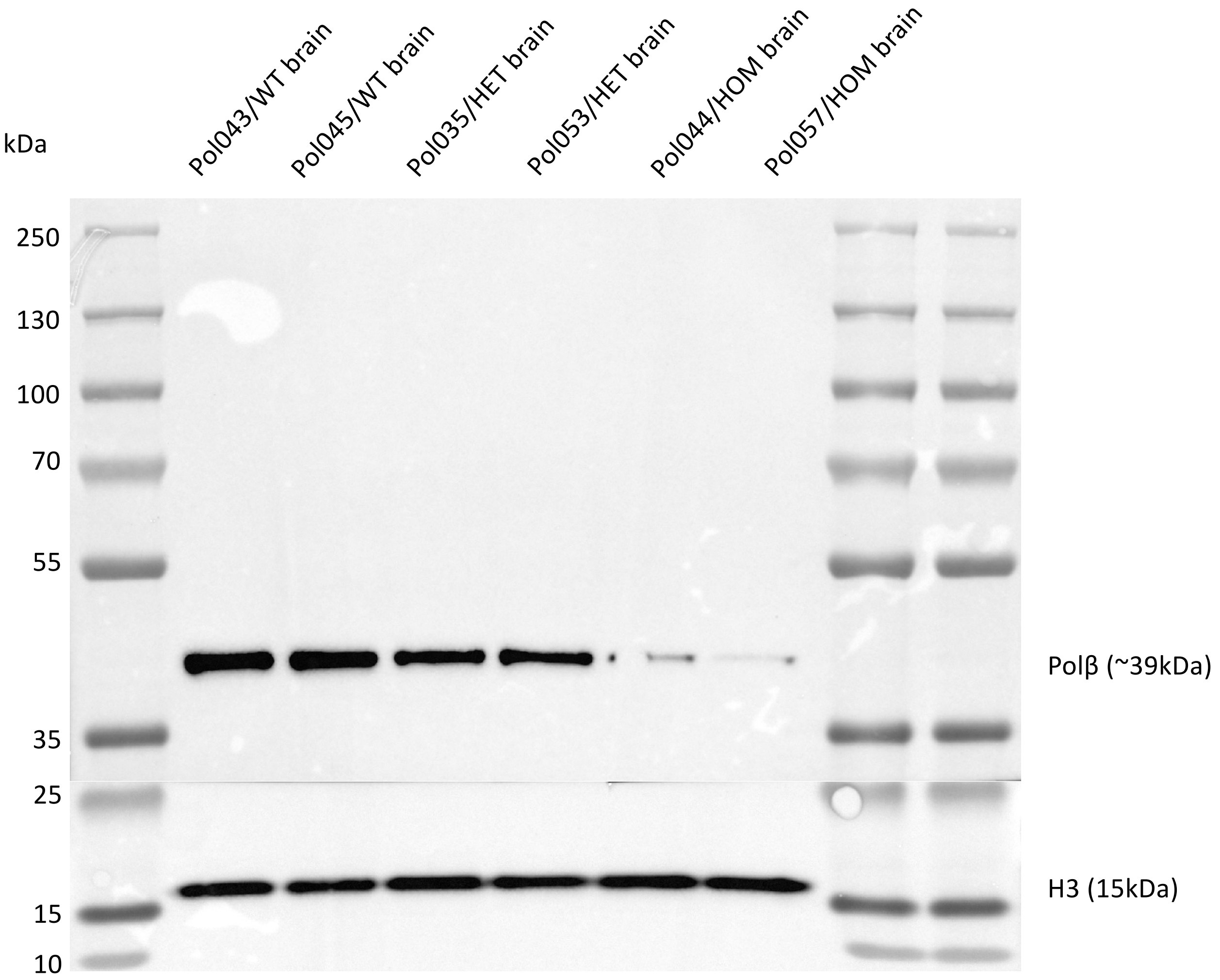

Loaded 30ug
Antibodies used:

1:1000 Anti-Polβ (Abcam, Cat# ab175197)
1:2000 Anti-H3 (Cell Signaling, Cat# 4499S)

WT: Pol043 x Pol045
HET: Pol035 x Pol053
HOM: Pol044 x Pol057
Run #2: 9/15/24

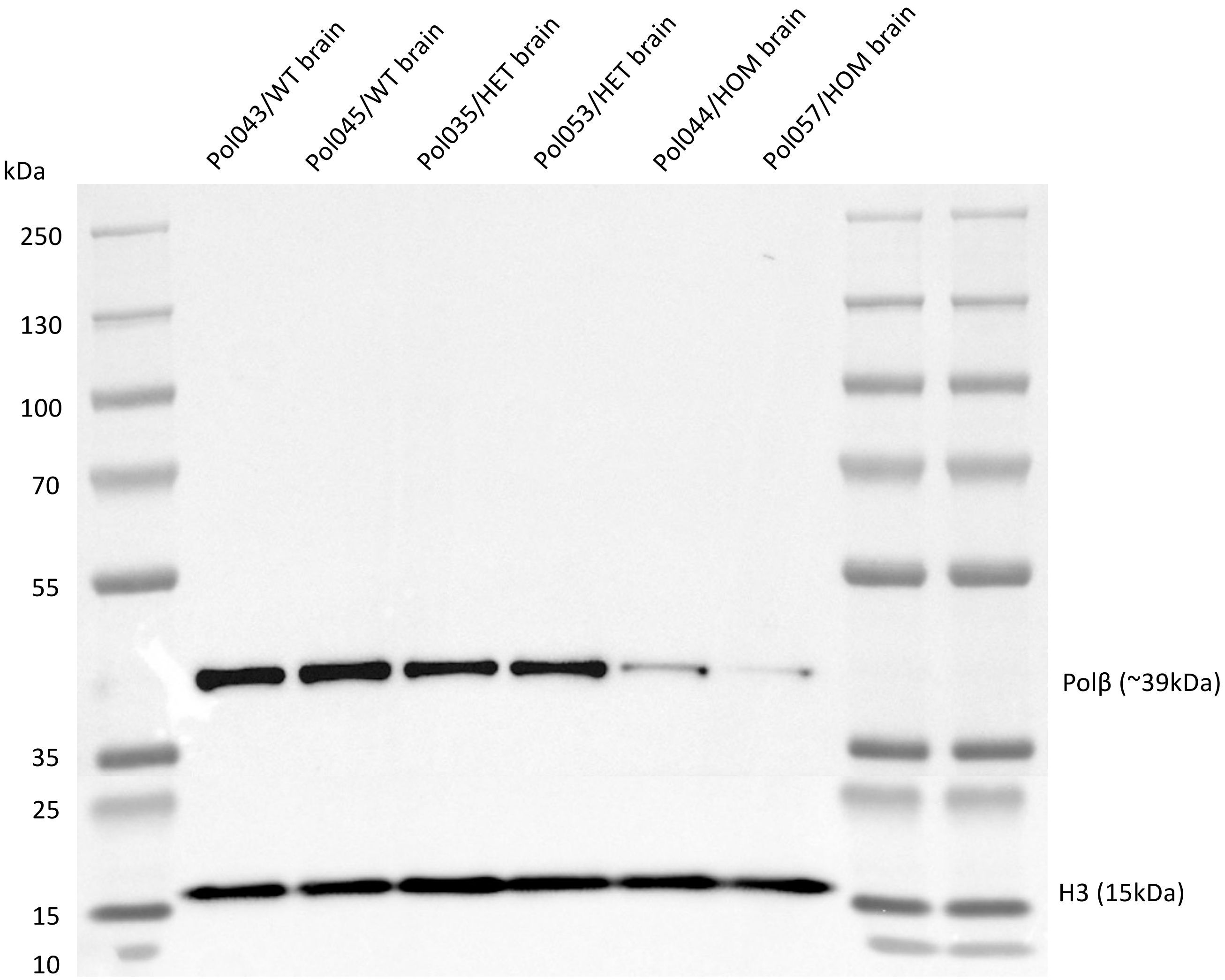

Loaded 30ug

Antibodies used:

1:1000 Anti-Polβ (Abcam, Cat# ab175197)

1:2000 Anti-H3 (Cell Signaling, Cat# 4499S)

WT: Pol043 x Pol045

HET: Pol035 x Pol053

HOM: Pol044 x Pol057

Run #3: 9/21/24

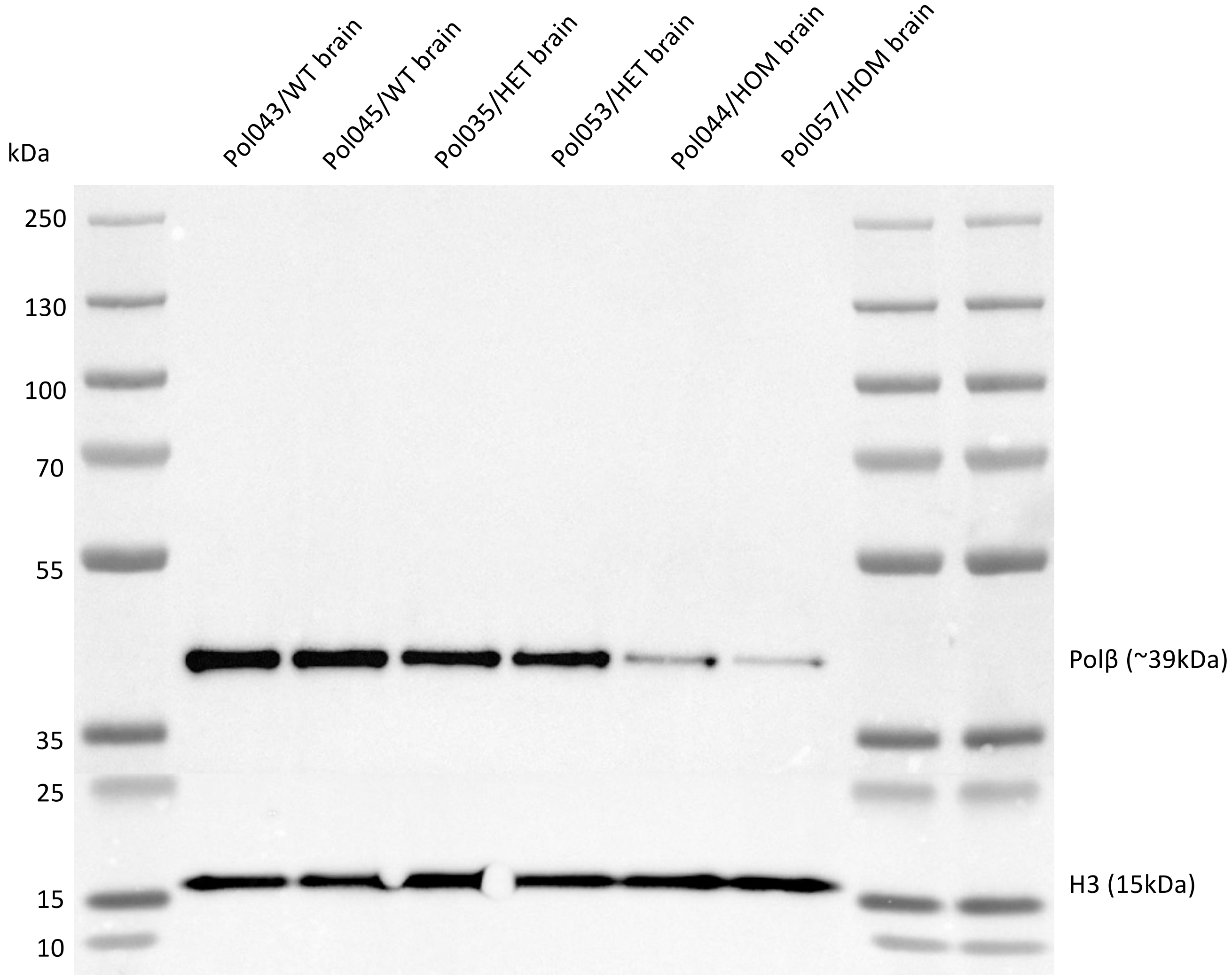

Loaded 30ug
Antibodies used:
1:1000 Anti-Polβ (Abcam, Cat# ab175197)
1:2000 Anti-H3 (Cell Signaling, Cat# 4499S)

WT: Pol105 x Pol116
HET: Pol113 x Pol115
HOM: Pol125 x Pol127
Run #1: 11/2/24

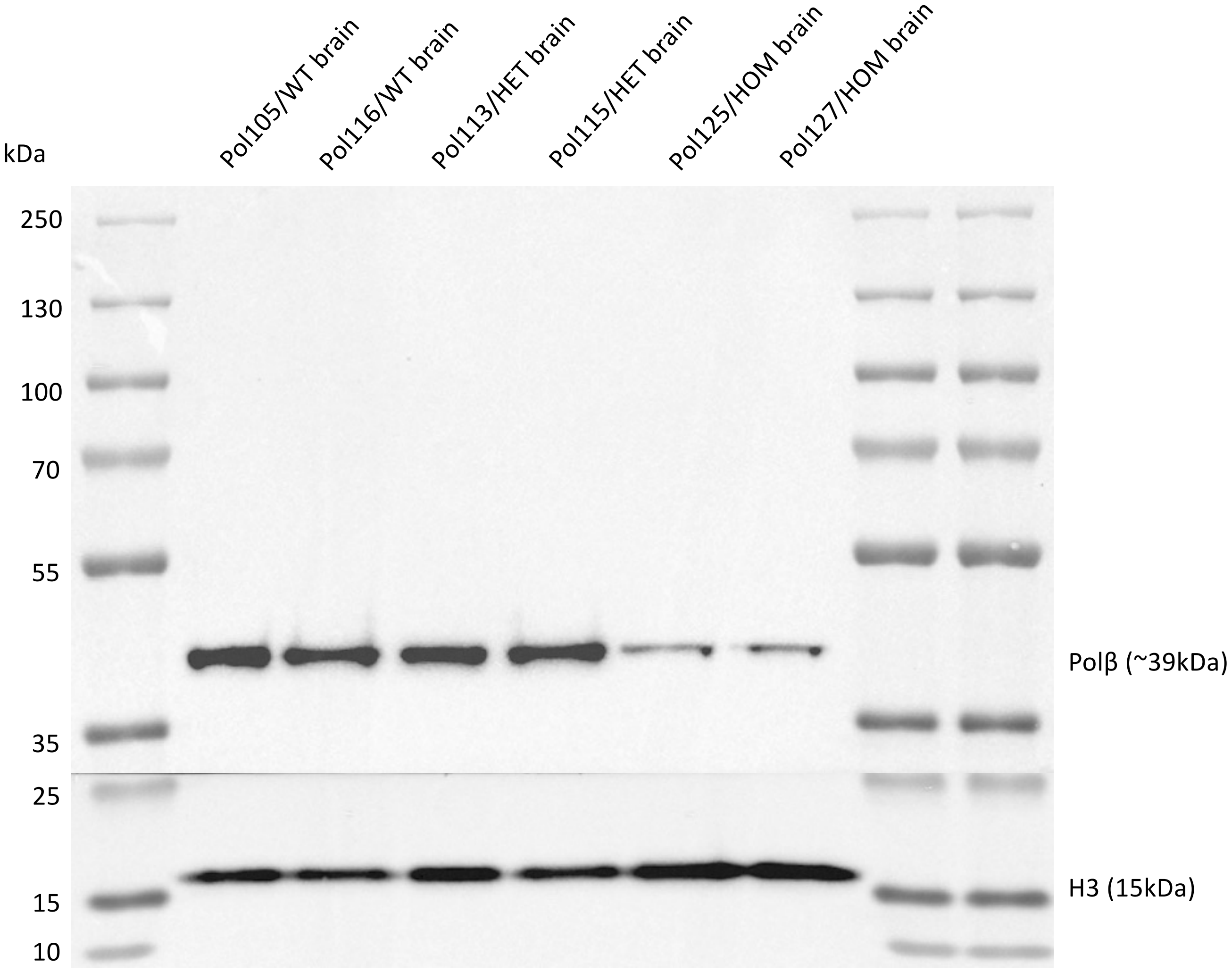

Loaded 30ug
Antibodies used:

1:1000 Anti-Polβ (Abcam, Cat# ab175197)
1:2000 Anti-H3 (Cell Signaling, Cat# 4499S)

WT: Pol105 x Pol116
HET: Pol113 x Pol115
HOM: Pol125 x Pol127
Run #2: 11/12/24

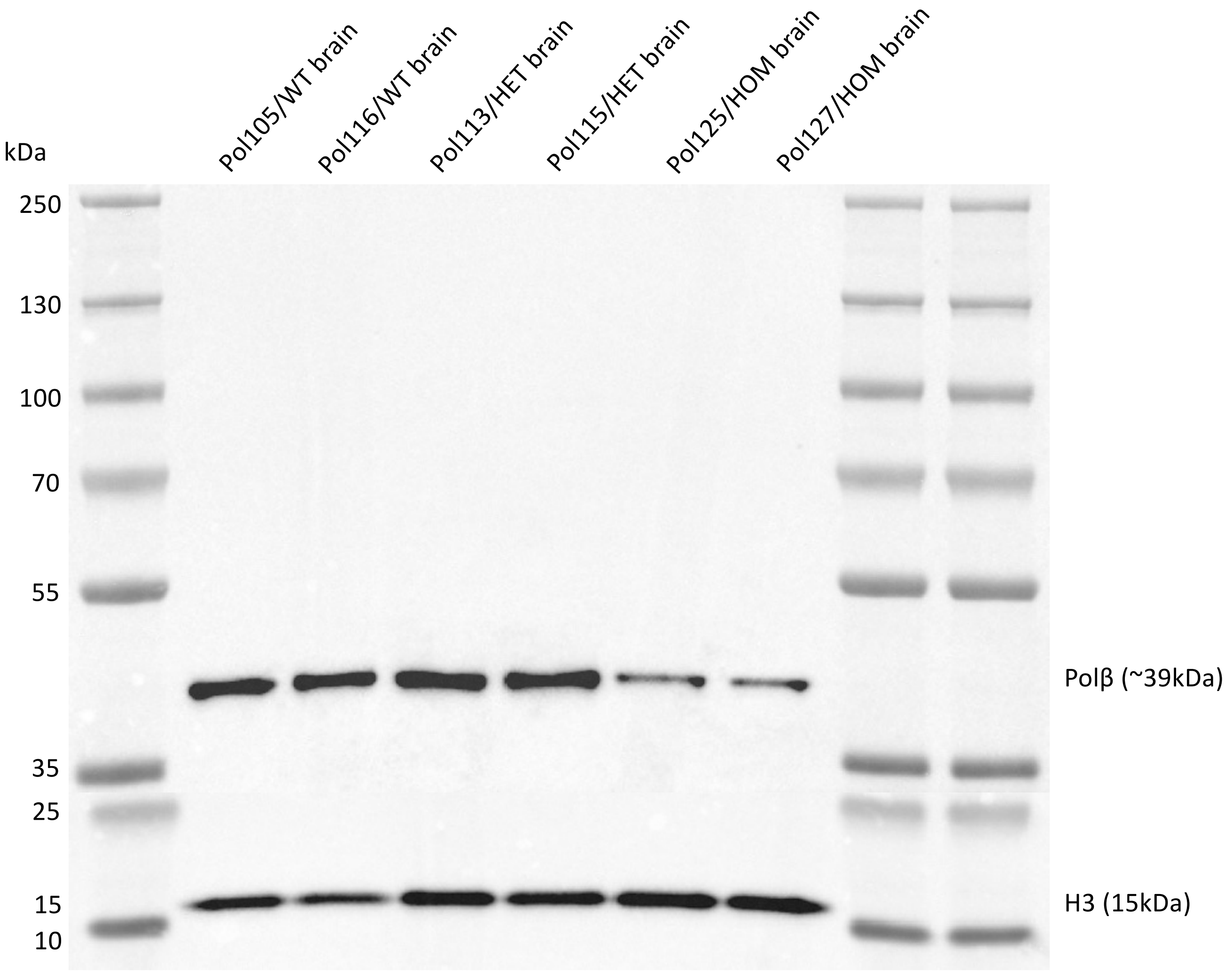

Loaded 30ug
Antibodies used:
1:1000 Anti-Polβ (Abcam, Cat# ab175197)
1:2000 Anti-H3 (Cell Signaling, Cat# 4499S)

WT: Pol105 x Pol116
HET: Pol113 x Pol115
HOM: Pol125 x Pol127
Run #3: 11/16/24

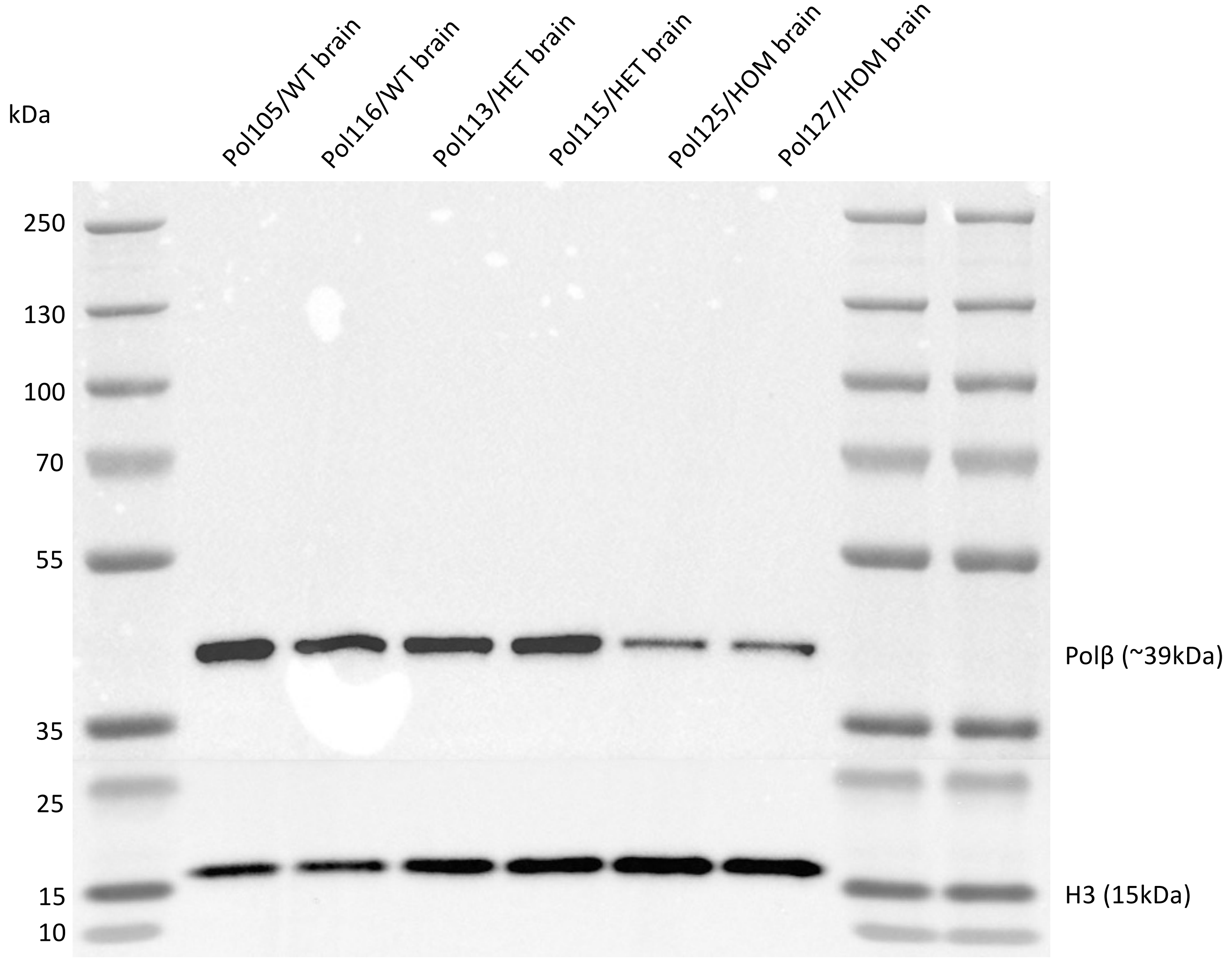

Loaded 30ug

Antibodies used:

1:1000 Anti-Polβ (Abcam, Cat# ab175197)

1:2000 Anti-H3 (Cell Signaling, Cat# 4499S)

WT: Pol118 x Pol122

HET: Pol117 x Pol119

HOM: Pol130 x Pol143

Run #1: 11/12/24

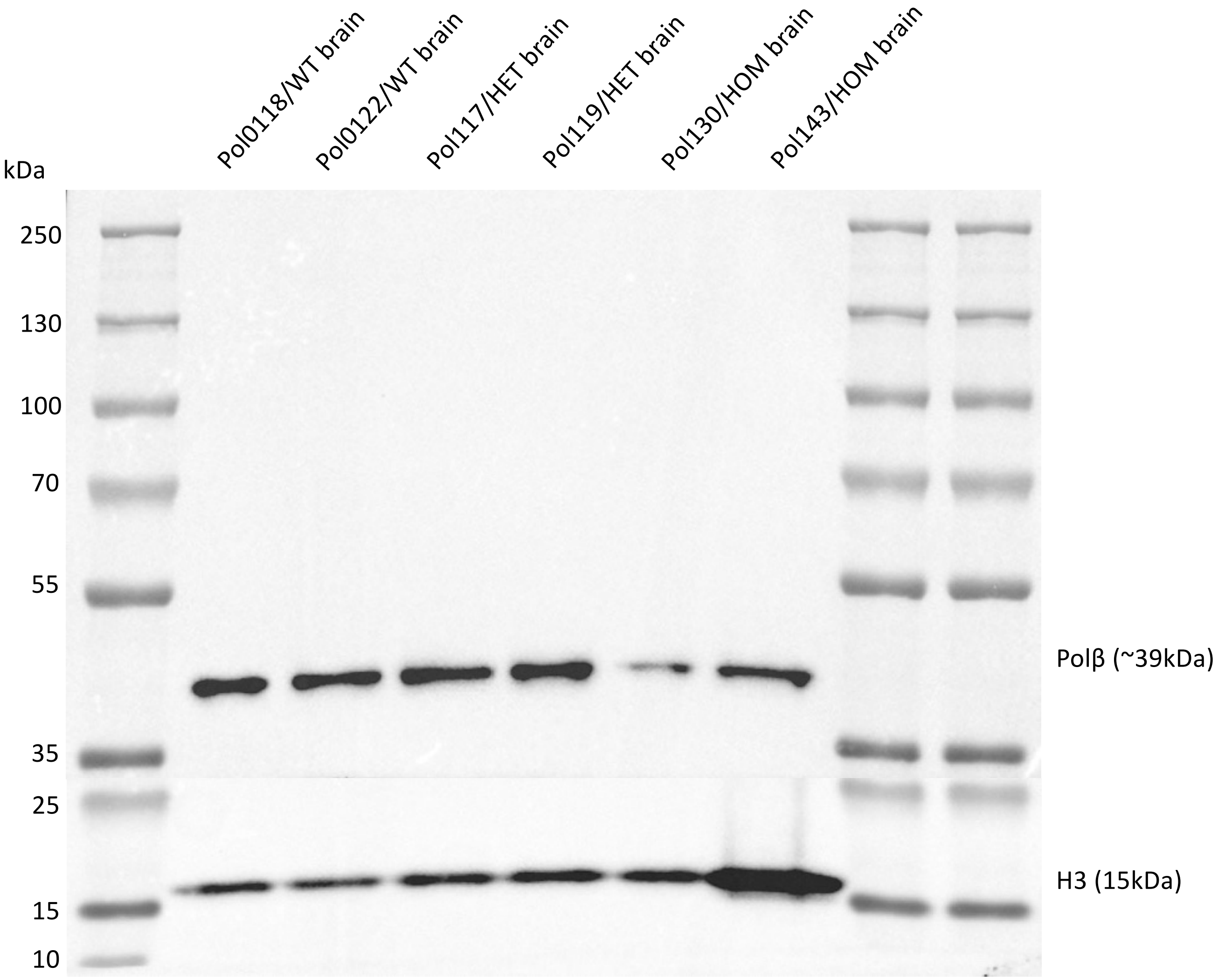

Loaded 30ug

Antibodies used:

1:1000 Anti-Polβ (Abcam, Cat# ab175197)

1:2000 Anti-H3 (Cell Signaling, Cat# 4499S)

WT: Pol118 x Pol122

HET: Pol117 x Pol119

HOM: Pol130 x Pol143

Run #2: 11/16/24

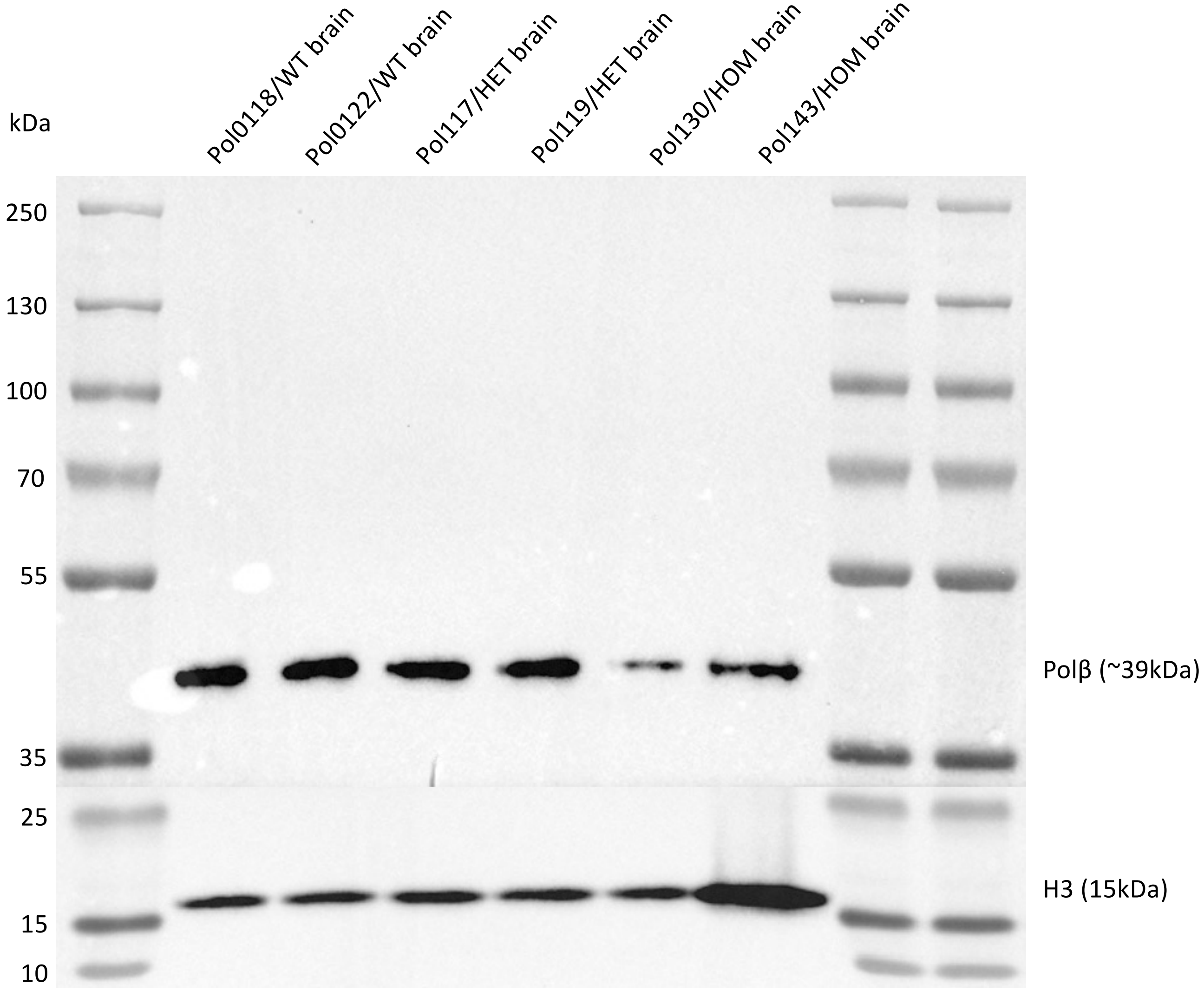

Loaded 30ug
Antibodies used:
1:1000 Anti-Polβ (Abcam, Cat# ab175197)
1:2000 Anti-H3 (Cell Signaling, Cat# 4499S)

WT: Pol118 x Pol122
HET: Pol117 x Pol119
HOM: Pol130 x Pol143
Run #3: 12/9/24

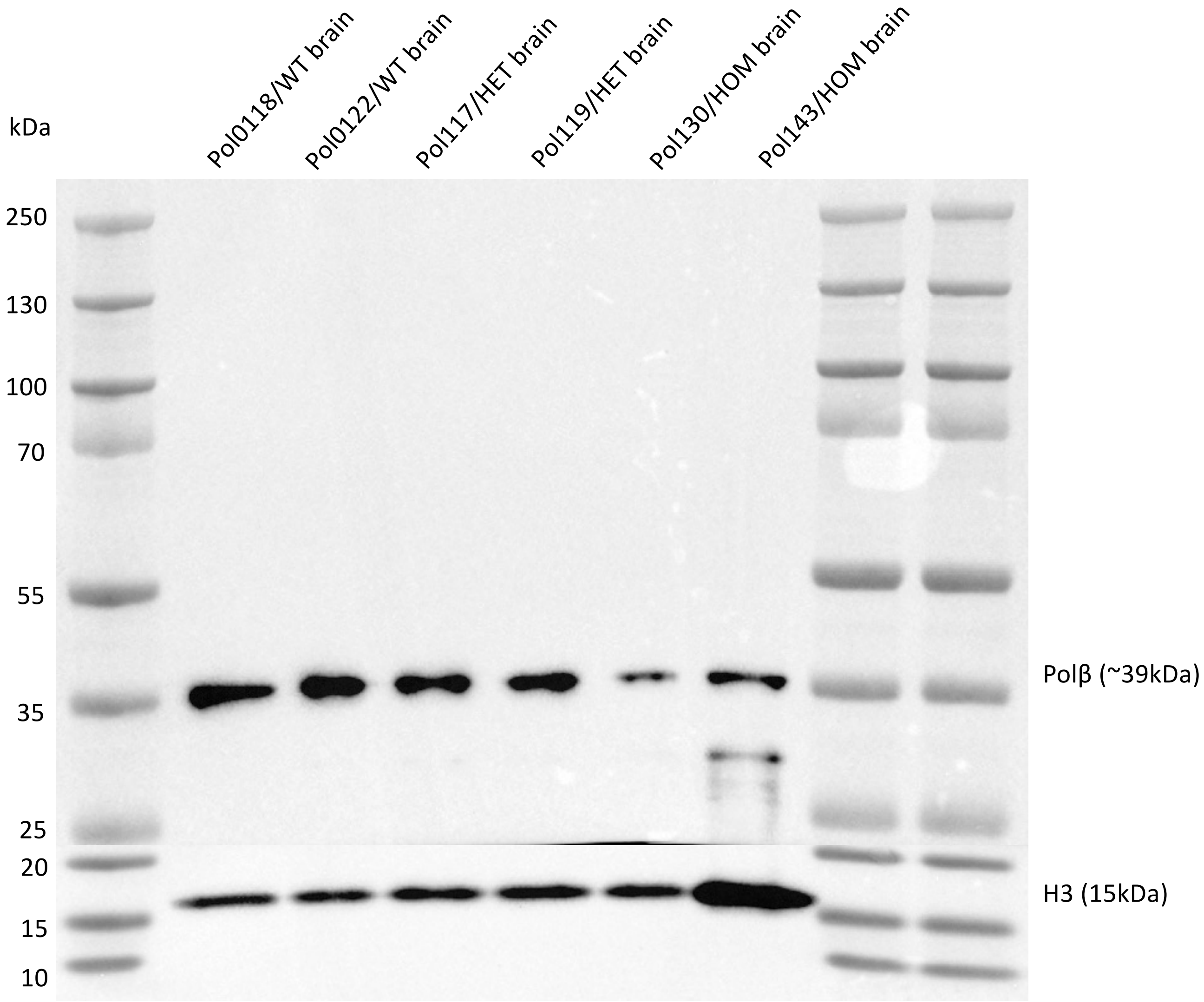

Figure S3: Body weight analysis from Cohort #2.

(A) Table of details for each mouse from Cohort #2, **(B)** Plot of body weight for each mouse in Cohort #2 (gms), and **(C)** Average body weight, comparing the WT and HOM mice in cohort #2, p<*0.05; t-Test, unpaired, Normal (Gaussian), Welch's t Test.

Supplementary Figure S3

Mouse Cohort #2
Body Weight Analysis

A.

Mouse #	Genotype	DOB	NecDate	Age weeks	Sex	BWT	BWT Comment
Pol083	WT	3/7/24	6/18/24	14.71	M	27.26	Below range for age and sex
Pol081	WT	3/4/24	6/18/24	15.14	F	21.31	Within range for age and sex
Pol075	WT	3/4/24	6/18/24	15.14	F	22.6	Within range for age and sex
Pol069	WT	3/2/24	6/18/24	15.43	M	30.51	Within range for age and sex
Pol110	HOM	4/9/24	6/18/24	10.00	M	18.81	Low for age and sex
Pol101	HOM	3/26/24	6/18/24	12.00	M	24.88	Below range for age and sex
Pol097	HOM	3/25/24	6/18/24	12.14	M	21.19	Within range for age and sex
Pol082	HOM	3/7/24	6/18/24	14.71	F	15.8	Low for age and sex
Pol077	HOM	3/4/24	6/18/24	15.14	F	16.33	Low for age and sex
Pol076	HOM	3/4/24	6/18/24	15.14	F	16.88	Low for age and sex
Pol074	HOM	3/4/24	6/18/24	15.14	M	20.97	Low for age and sex
Pol072	HOM	3/2/24	6/18/24	15.43	F	17.32	Low for age and sex

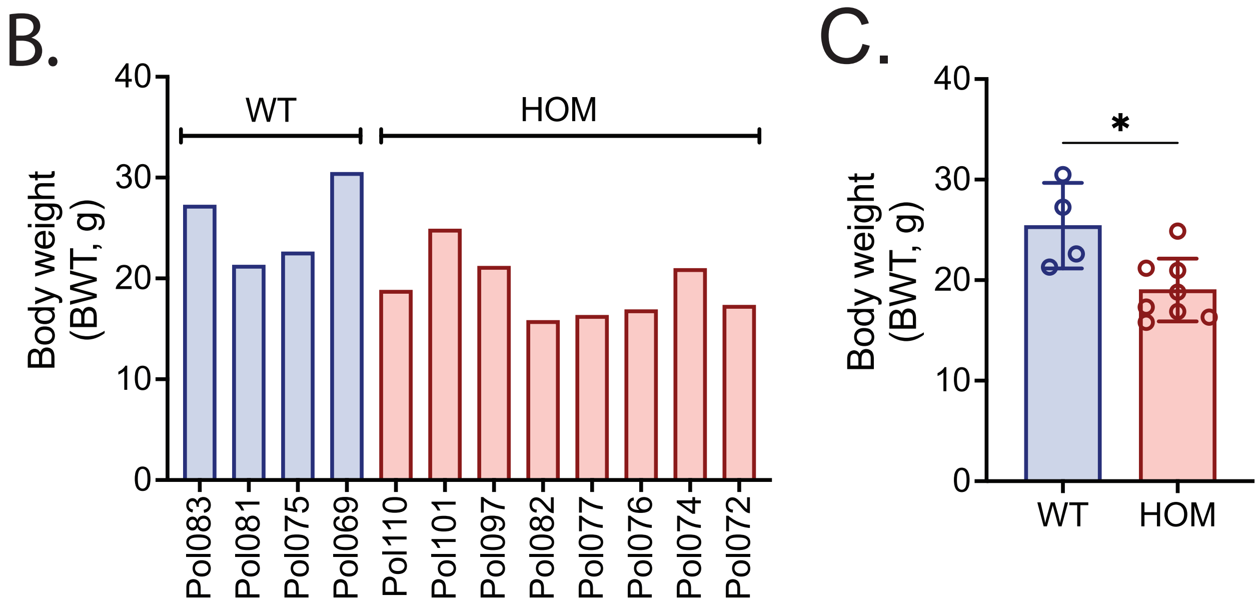

Figure S4: Immunoblots from micro-dissected brain regions isolated from Cohort #3. Immunoblots of cerebellum, midibrain, striatum, and cortex isolates from WT, and HOM mice (Cohort #3), probing for Polβ. H3 was used as a loading control. α-actinin was used as a secondary loading control and validation for brain region specificity. Sample names above each lane indicate the mouse identification number, with the genotype listed after each number. These full blots represent all samples used to develop the quantitative analysis shown in **Figure 4C**.

Supplementary Figure S4
24mo Brain Regions samples
Mouse Cohort #3
Immunoblots

Hypomorphic protein expression of DNA polymerase beta in Polβ$^{L301R-V303R/L301R-V303R}$ knock-in transgenic mice does not impact global DNA methylation levels in the midbrain

Bryce Jacobs[1], Dan Ivanov[1], Ivana Brazza[2,3], Chris Faulk[4], Carmen J. Booth[5], Raquel Mattos-Canedo[1], Lucas Tian[1], Kaitlyn DePietro[1], Alper Uzun[1], Wynand P. Roos[1], Laurie H. Sanders[2,3], and Robert W. Sobol[1]*

Loaded 30ug

Antibodies used:

1:1000 Anti-Polβ (Abcam, Cat# ab175197)

1:2000 Anti-H3 (Cell Signaling, Cat# 4499S)

1:2000 Anti-α-actinin (Cell Signaling, Cat# 6487S)

Pol030 x Pol020
Run #1: 6/27/25

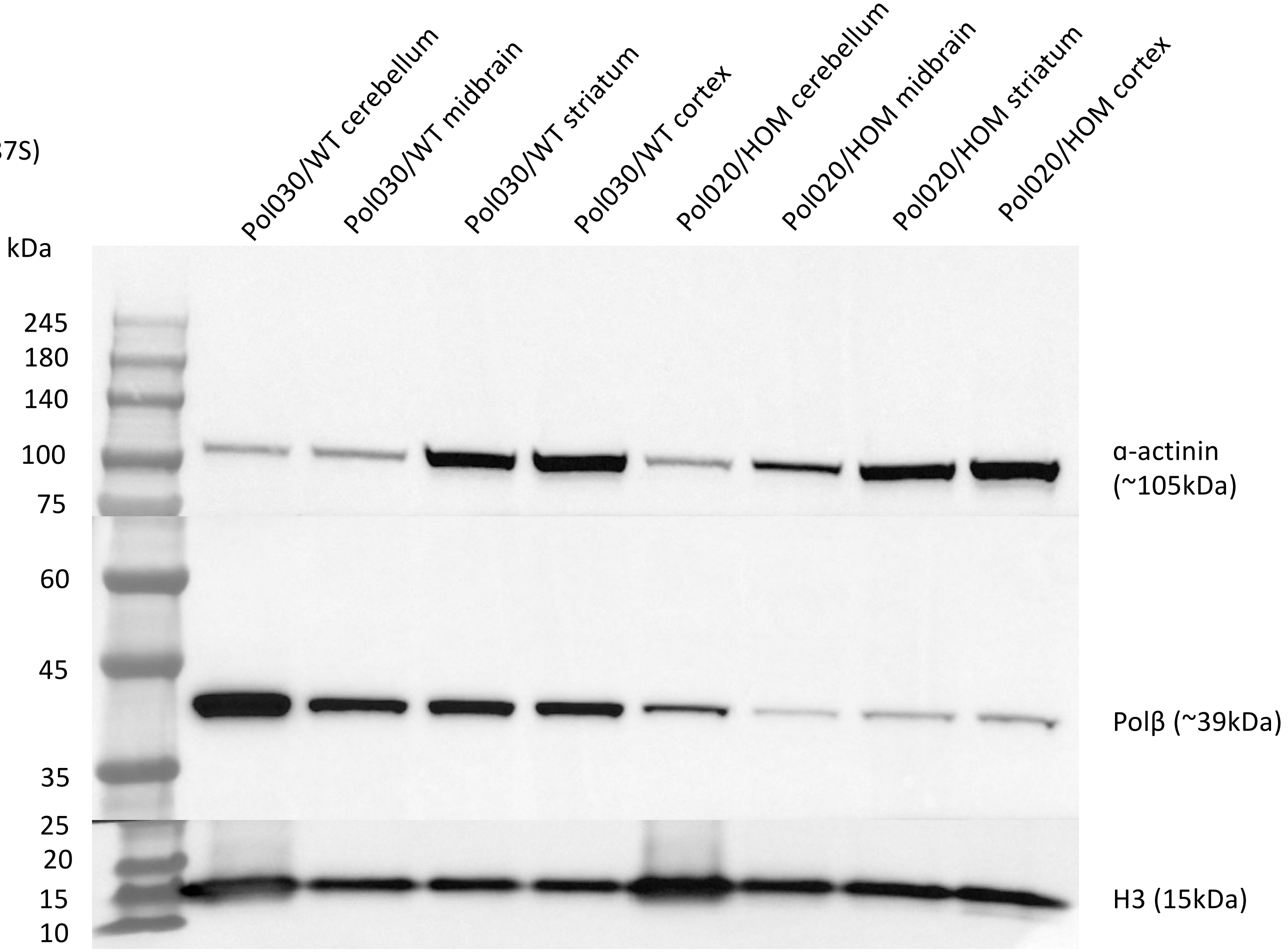

Loaded 30ug

Antibodies used:

- 1:1000 Anti-Polβ (Abcam, Cat# ab175197)
- 1:2000 Anti-H3 (Cell Signaling, Cat# 4499S)
- 1:2000 Anti-α-actinin (Cell Signaling, Cat# 6487S)

Pol030 x Pol020
Run #2: 7/8/25

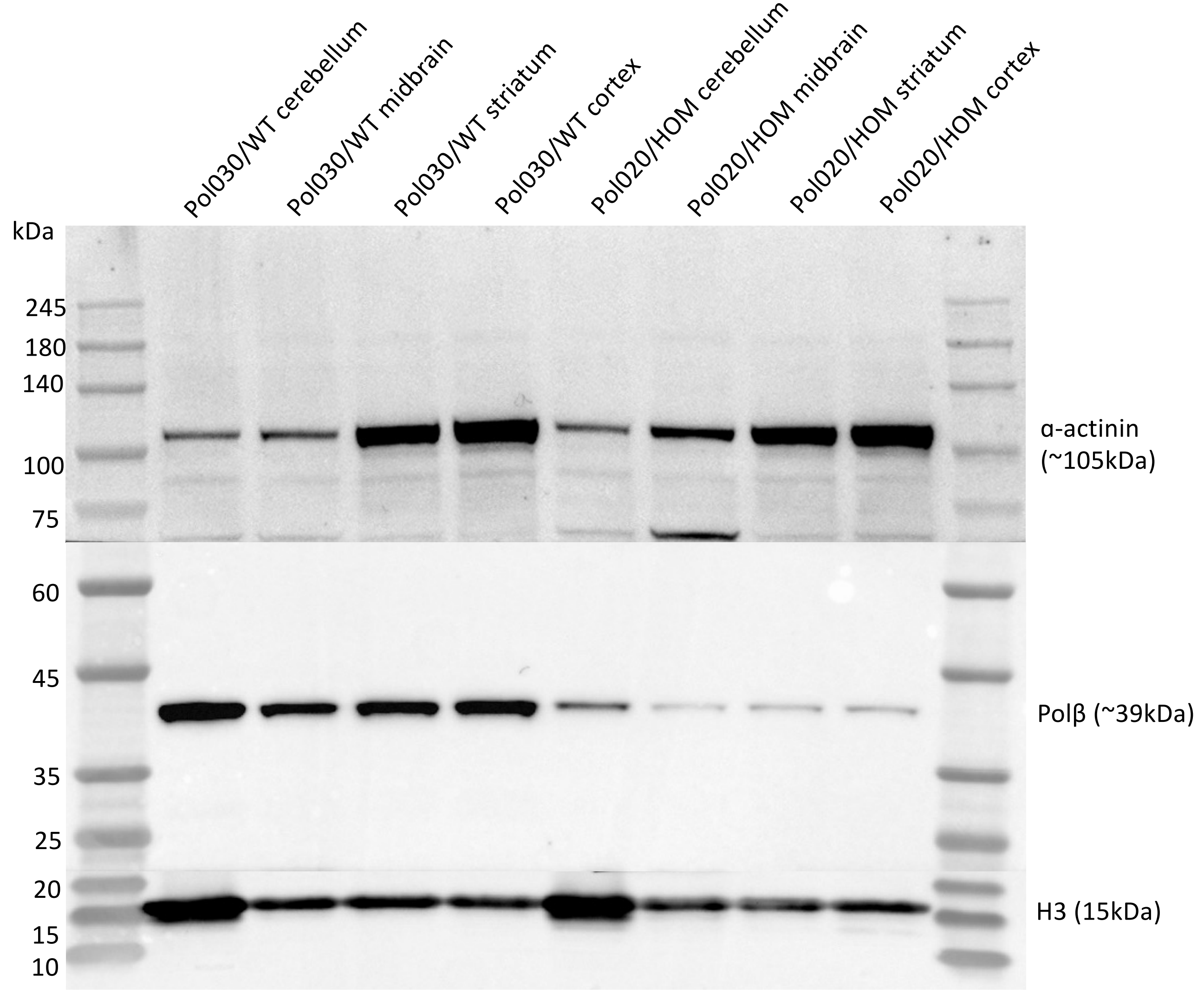

Loaded 30ug

Antibodies used:

1:1000 Anti-Polβ (Abcam, Cat# ab175197)

1:2000 Anti-H3 (Cell Signaling, Cat# 4499S)

1:2000 Anti-α-actinin (Cell Signaling, Cat# 6487S)

Pol030 x Pol020

Run #3: 7/22/25

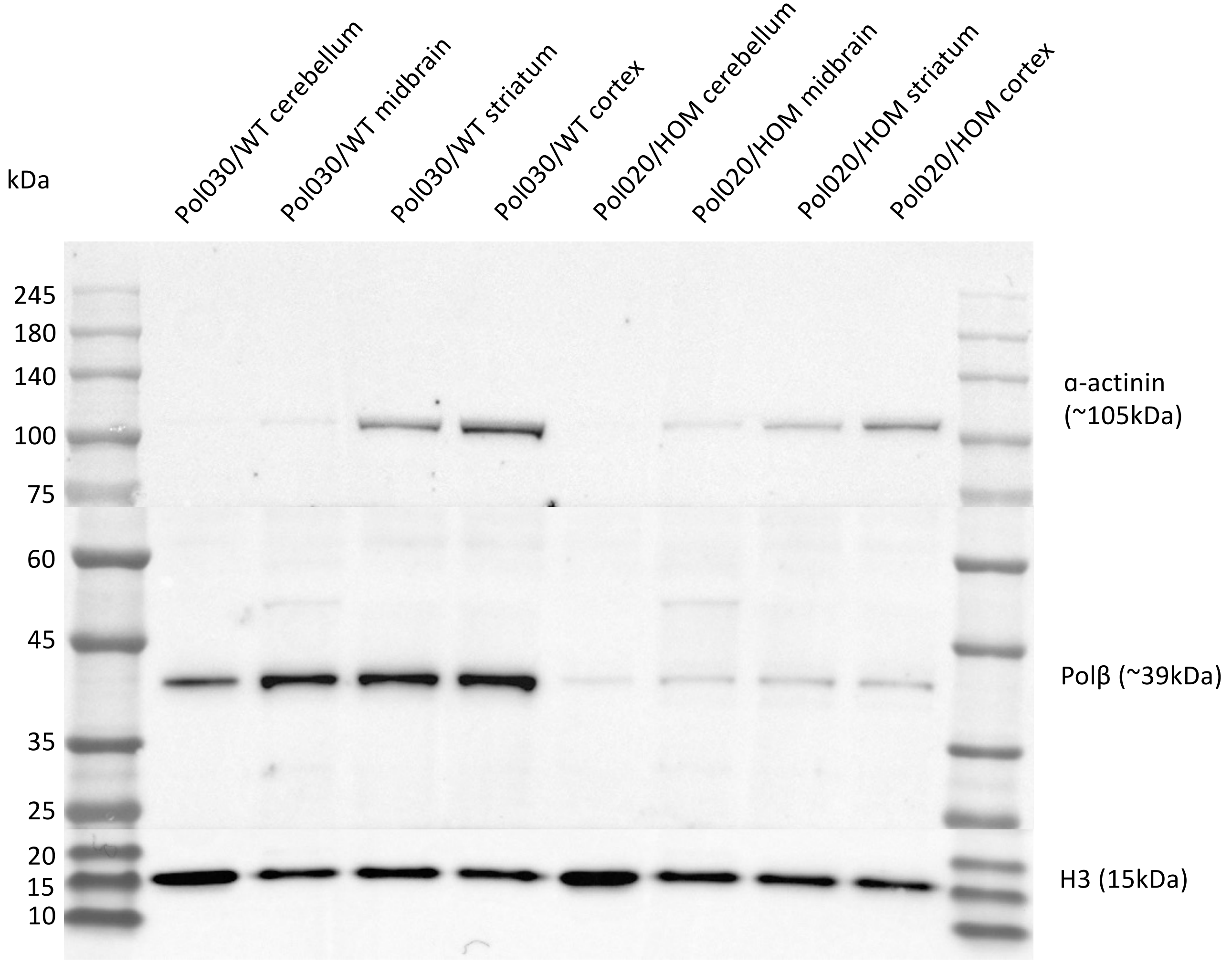

Loaded 30ug

Antibodies used:

1:1000 Anti-Polβ (Abcam, Cat# ab175197)

1:2000 Anti-H3 (Cell Signaling, Cat# 4499S)

1:2000 Anti-α-actinin (Cell Signaling, Cat# 6487S)

Pol066 x Pol000

Run #1: 7/3/25

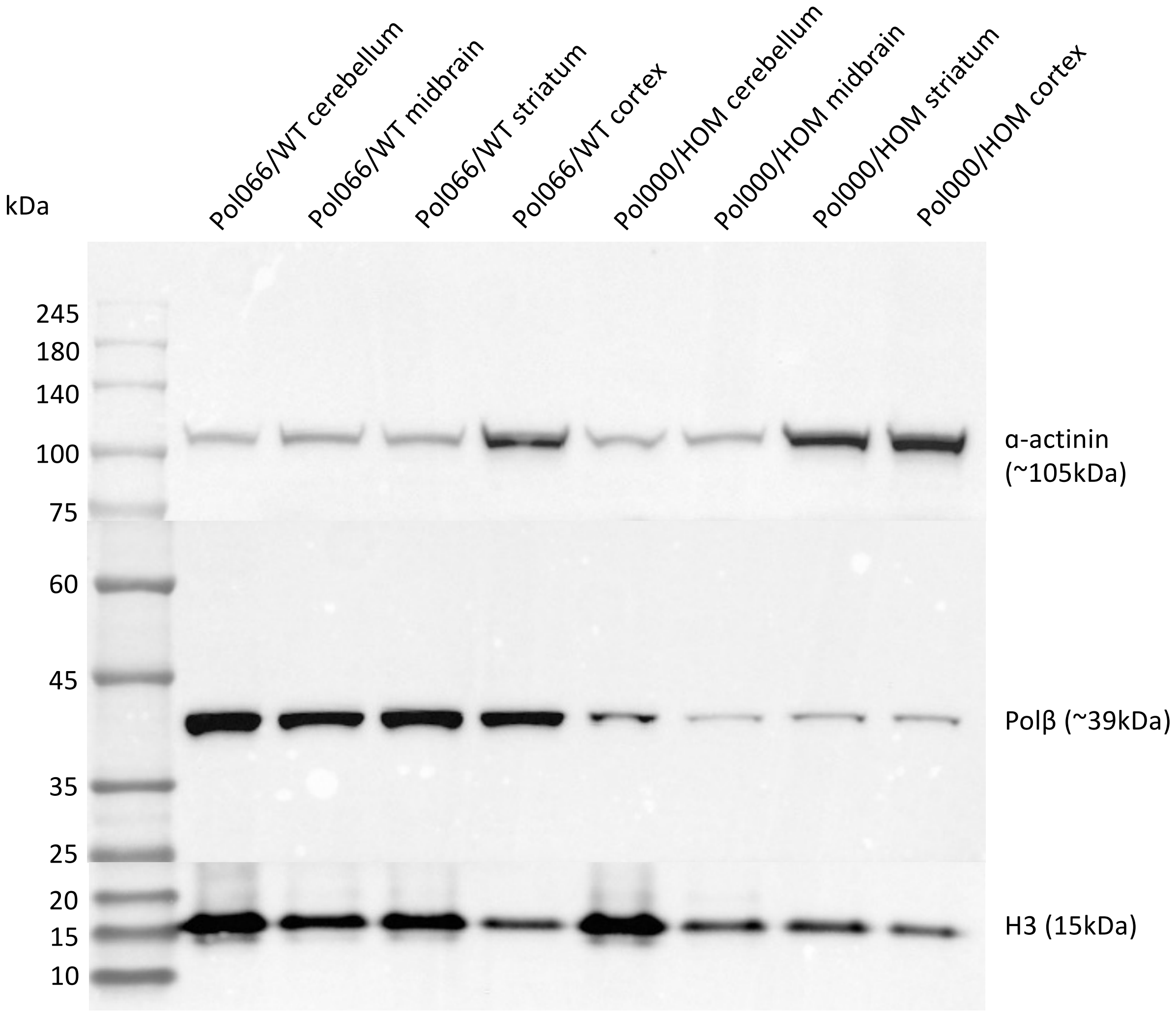

Loaded 30ug

Antibodies used:

1:1000 Anti-Polβ (Abcam, Cat# ab175197)

1:2000 Anti-H3 (Cell Signaling, Cat# 4499S)

1:2000 Anti-α-actinin (Cell Signaling, Cat# 6487S)

Pol066 x Pol000
Run #2: 7/8/25

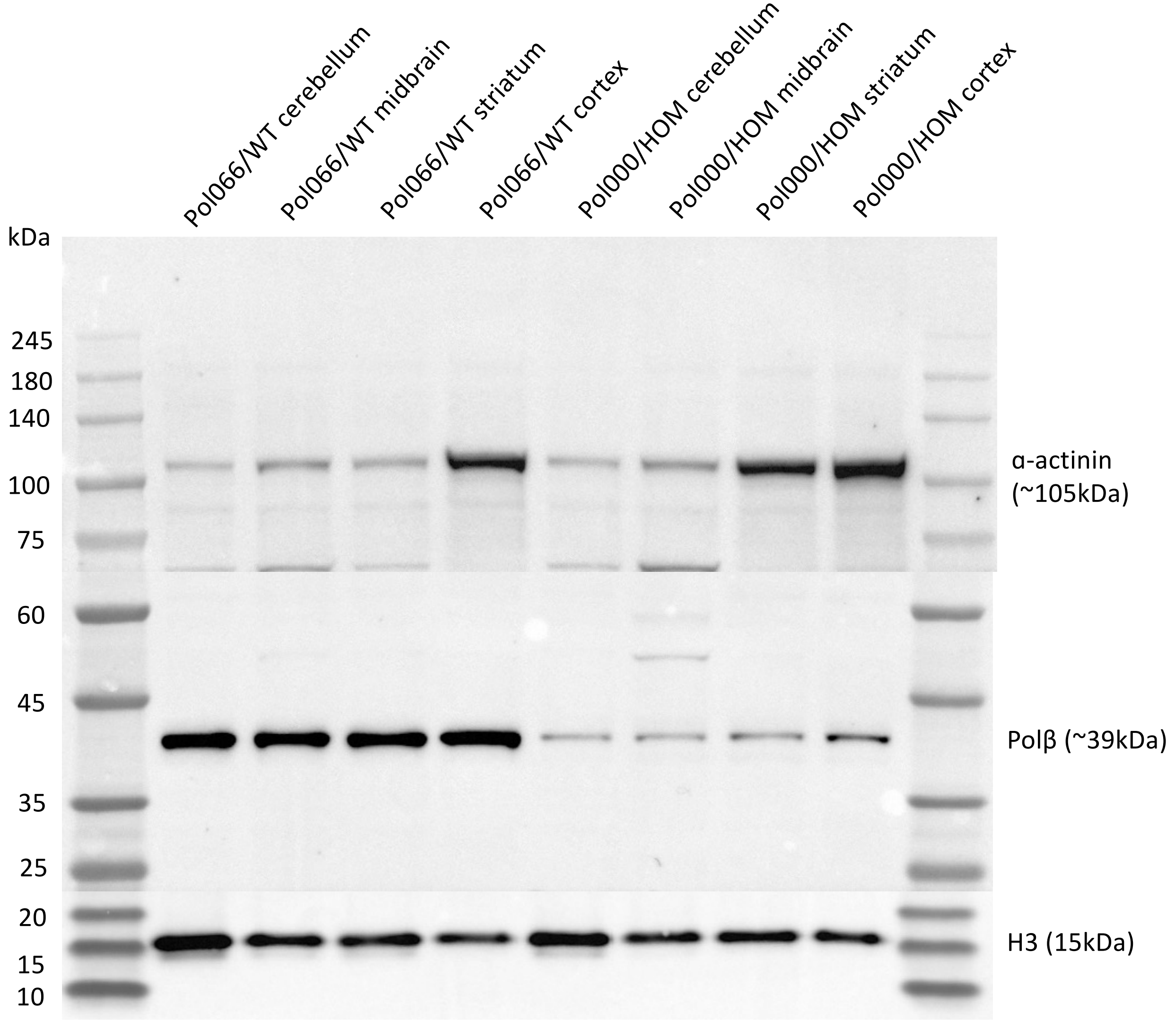

Loaded 30ug

Antibodies used:

1:1000 Anti-Polβ (Abcam, Cat# ab175197)
1:2000 Anti-H3 (Cell Signaling, Cat# 4499S)
1:2000 Anti-α-actinin (Cell Signaling, Cat# 6487S)

Pol066 x Pol000
Run #3: 7/18/25

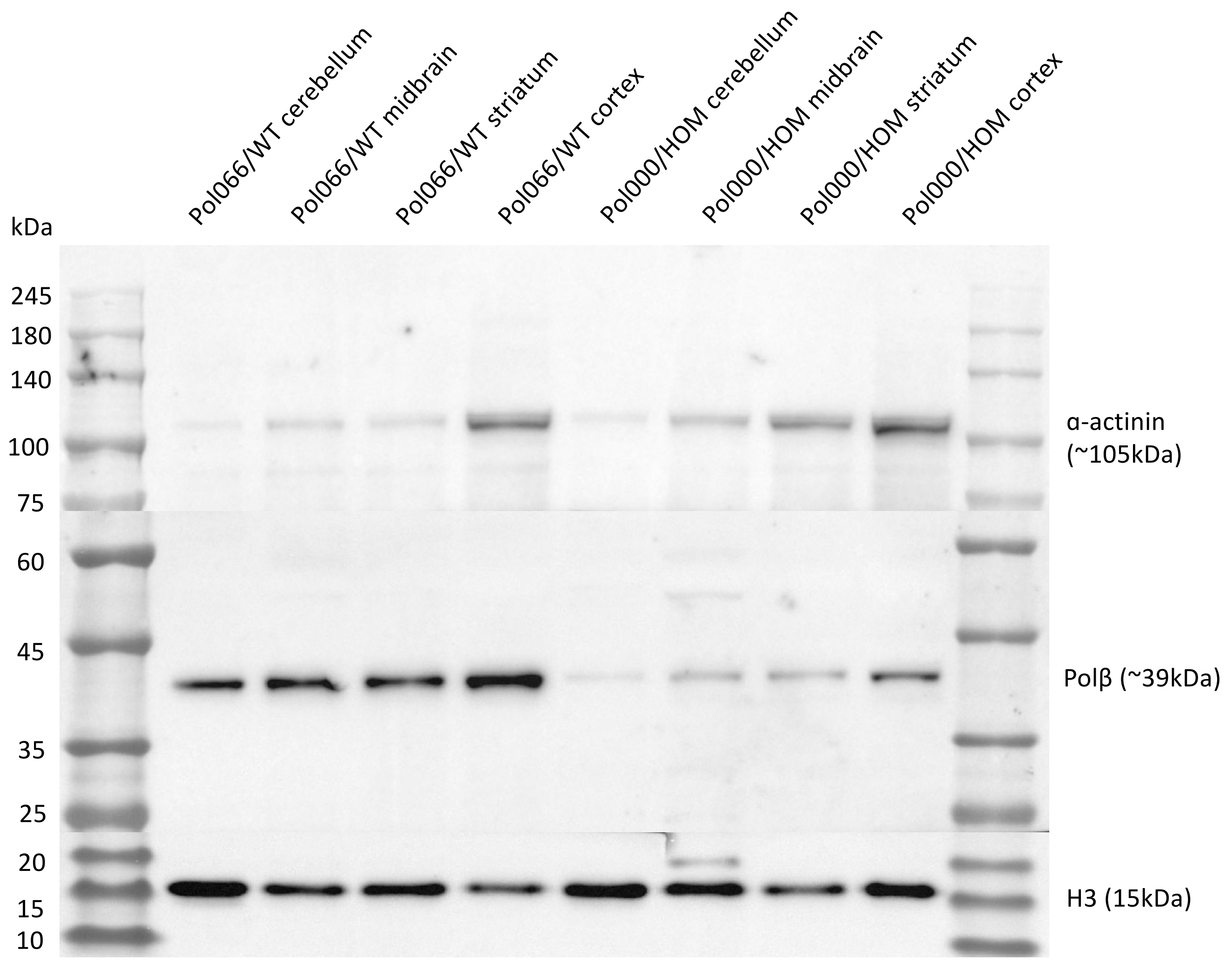

Loaded 30ug

Antibodies used:

1:1000 Anti-Polβ (Abcam, Cat# ab175197)

1:2000 Anti-H3 (Cell Signaling, Cat# 4499S)

1:2000 Anti-α-actinin (Cell Signaling, Cat# 6487S)

Pol042 x Pol013

Run #1: 7/15/25

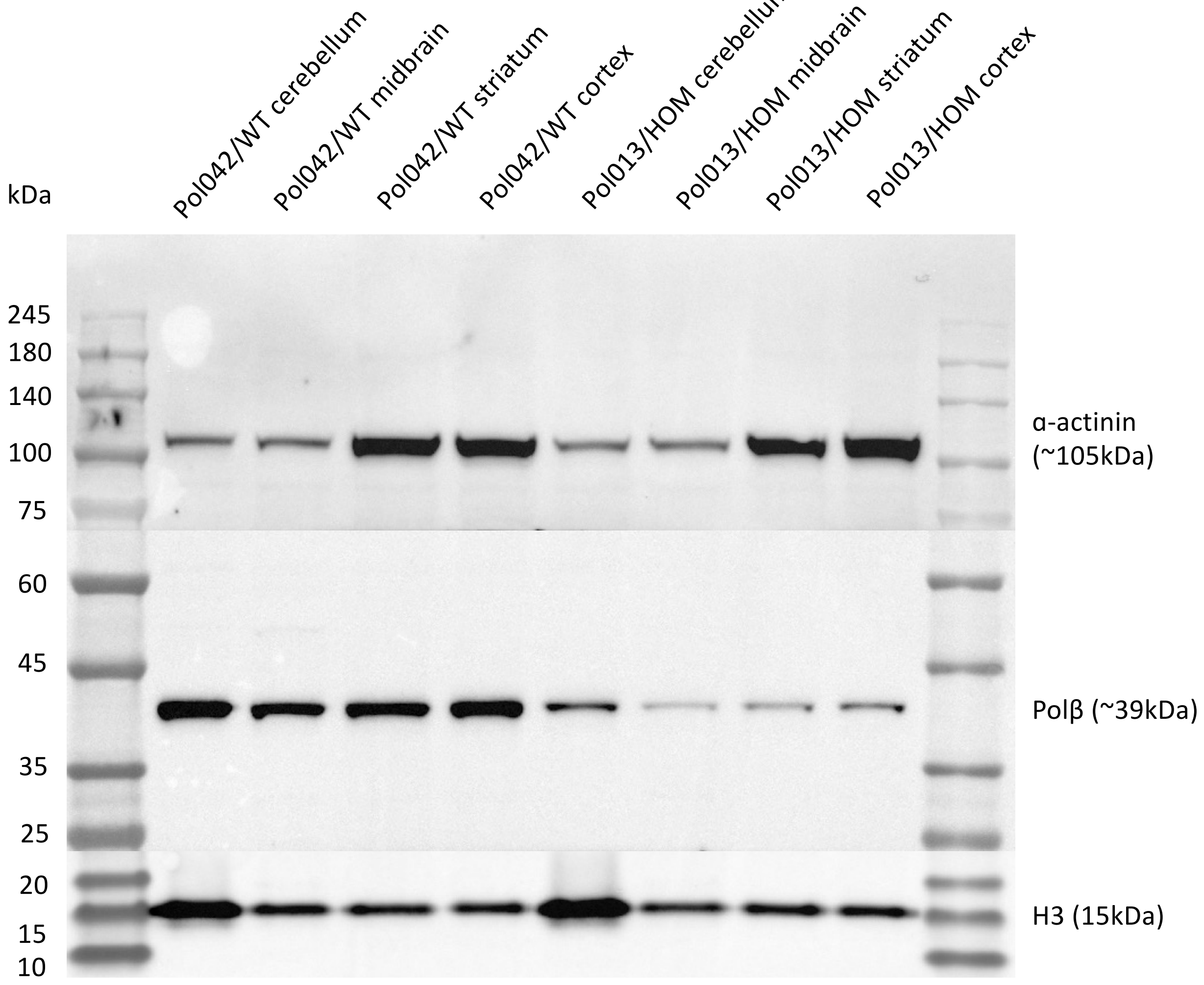

Loaded 30ug

Antibodies used:

1:1000 Anti-Polβ (Abcam, Cat# ab175197)

1:2000 Anti-H3 (Cell Signaling, Cat# 4499S)

1:2000 Anti-α-actinin (Cell Signaling, Cat# 6487S)

Pol042 x Pol013

Run #2: 7/17/25

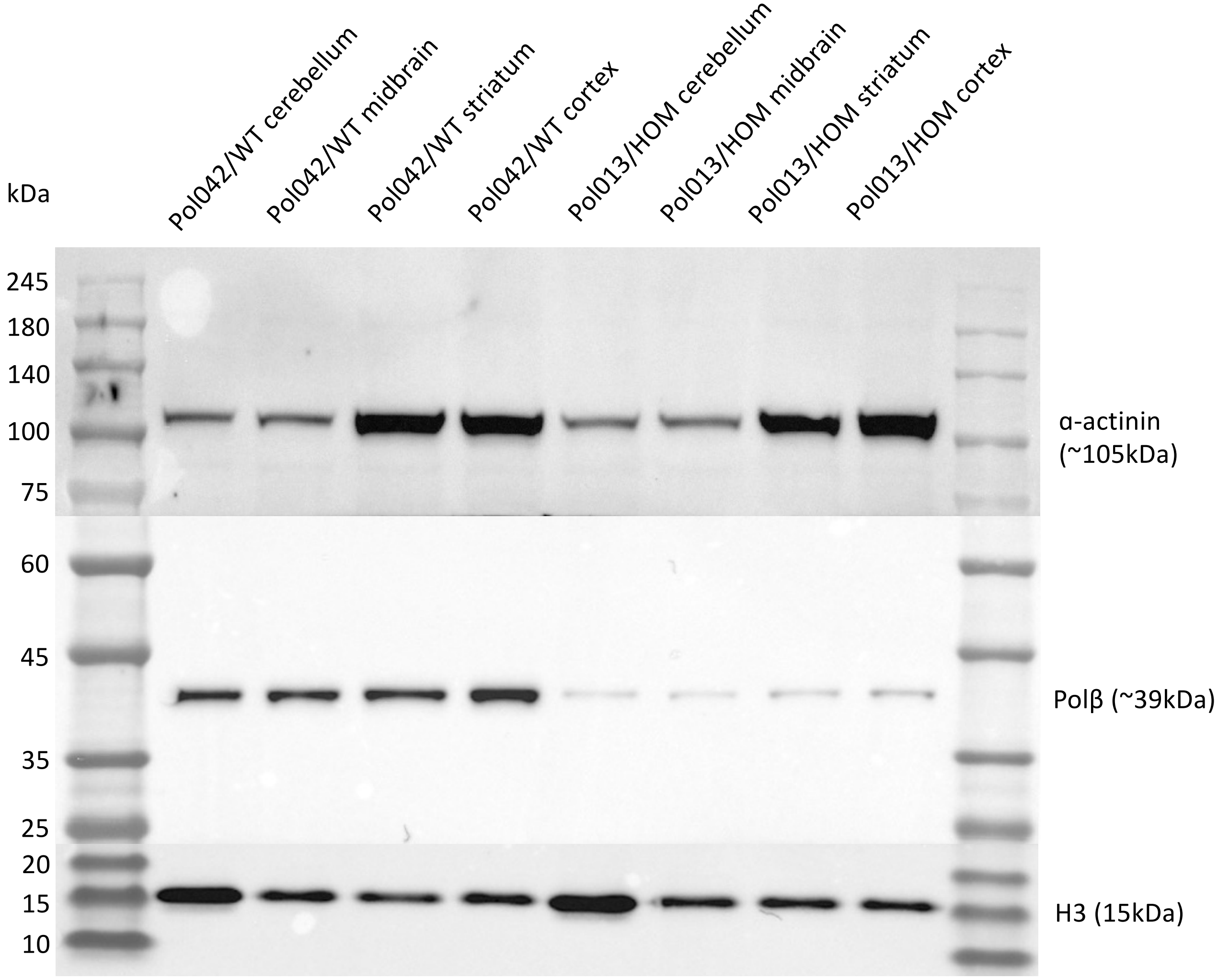

Loaded 30ug

Antibodies used:

1:1000 Anti-Polβ (Abcam, Cat# ab175197)

1:2000 Anti-H3 (Cell Signaling, Cat# 4499S)

1:2000 Anti-α-actinin (Cell Signaling, Cat# 6487S)

Pol042 x Pol013
Run #2: 7/25/25

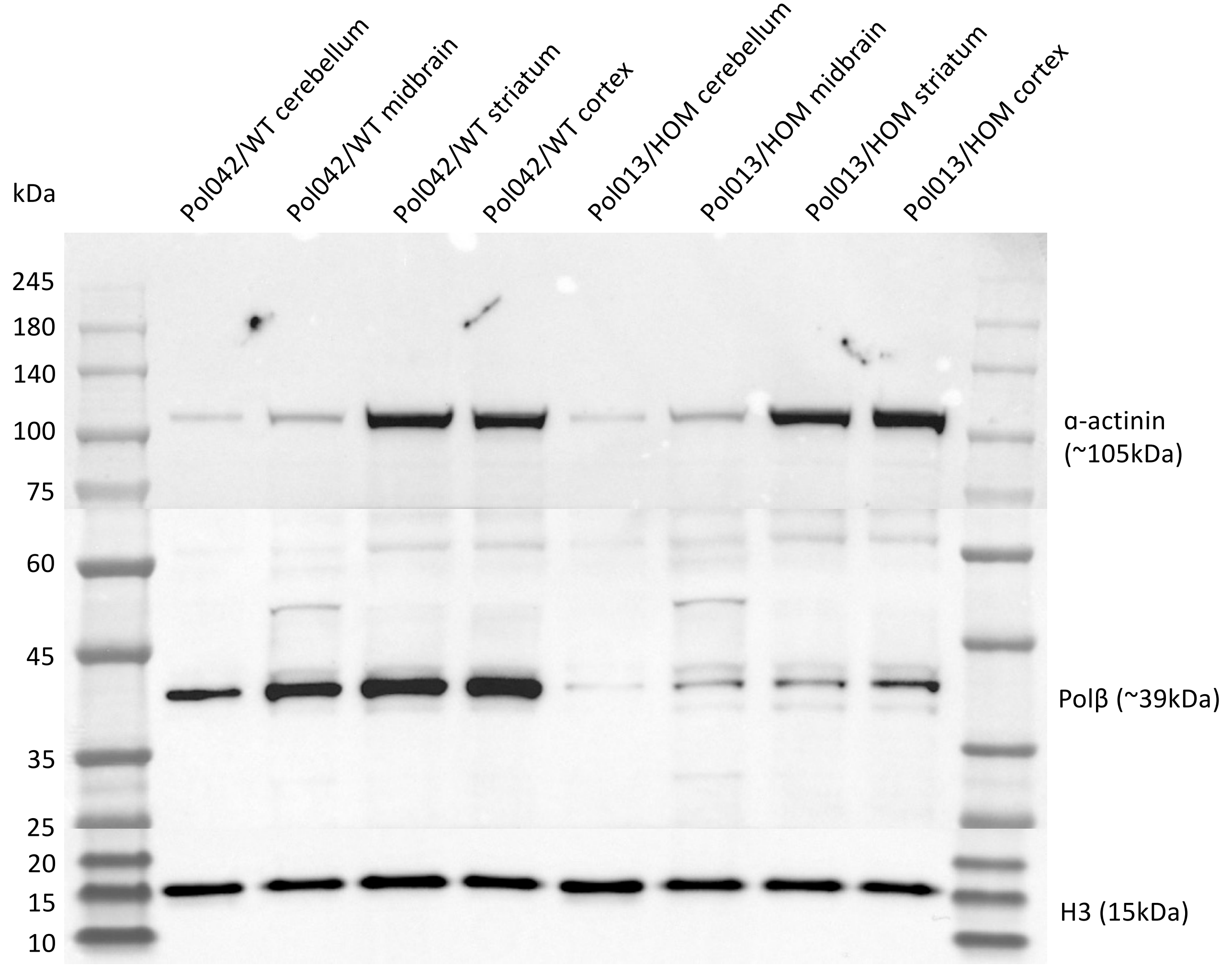

Loaded 30ug

Antibodies used:

- 1:1000 Anti-Polβ (Abcam, Cat# ab175197)
- 1:2000 Anti-H3 (Cell Signaling, Cat# 4499S)
- 1:2000 Anti-α-actinin (Cell Signaling, Cat# 6487S)

Pol067 x Pol003
Run #1: 7/15/25

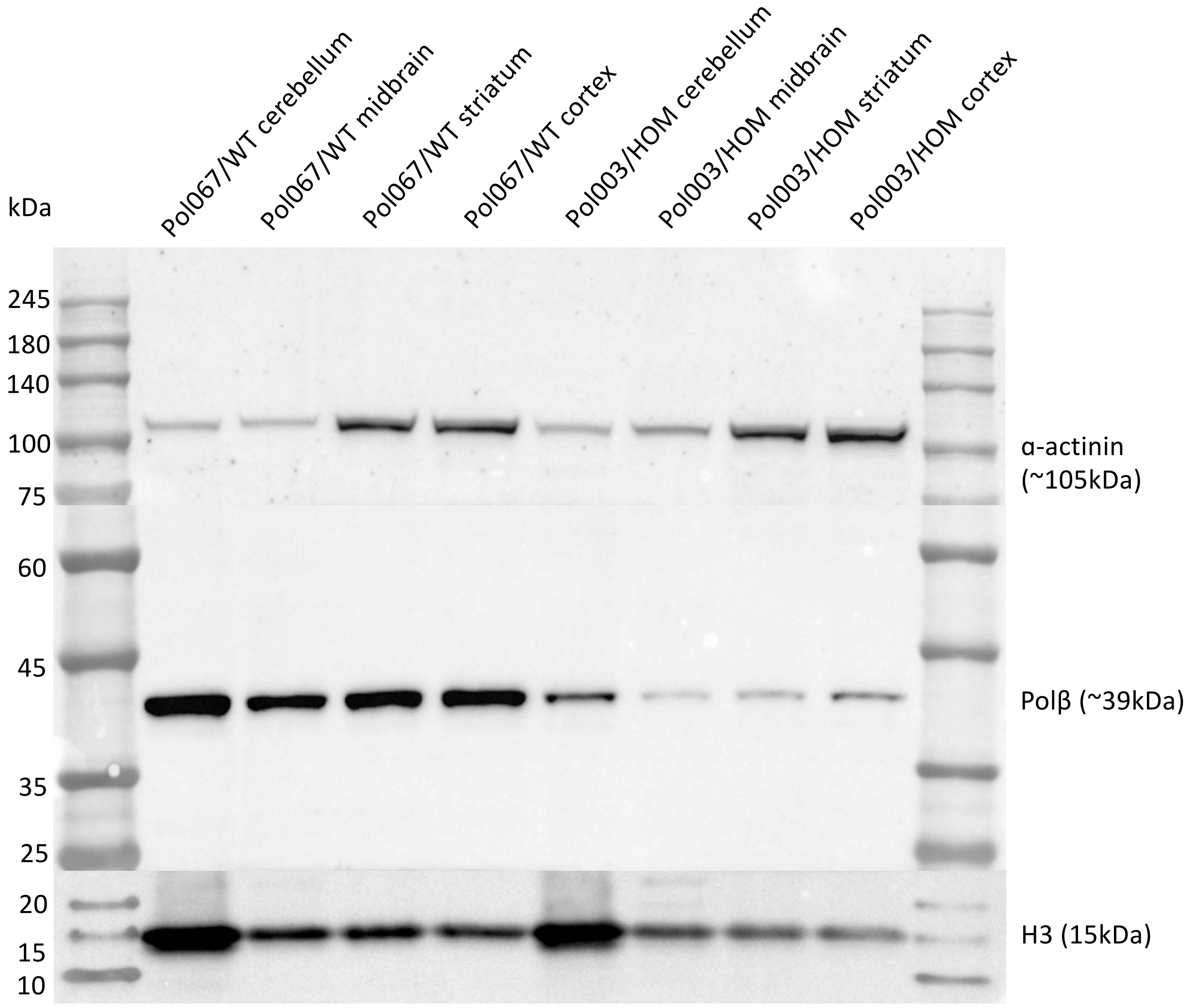

Loaded 30ug

Antibodies used:

- 1:1000 Anti-Polβ (Abcam, Cat# ab175197)
- 1:2000 Anti-H3 (Cell Signaling, Cat# 4499S)
- 1:2000 Anti-α-actinin (Cell Signaling, Cat# 6487S)

Pol067 x Pol003

Run #2: 7/18/25

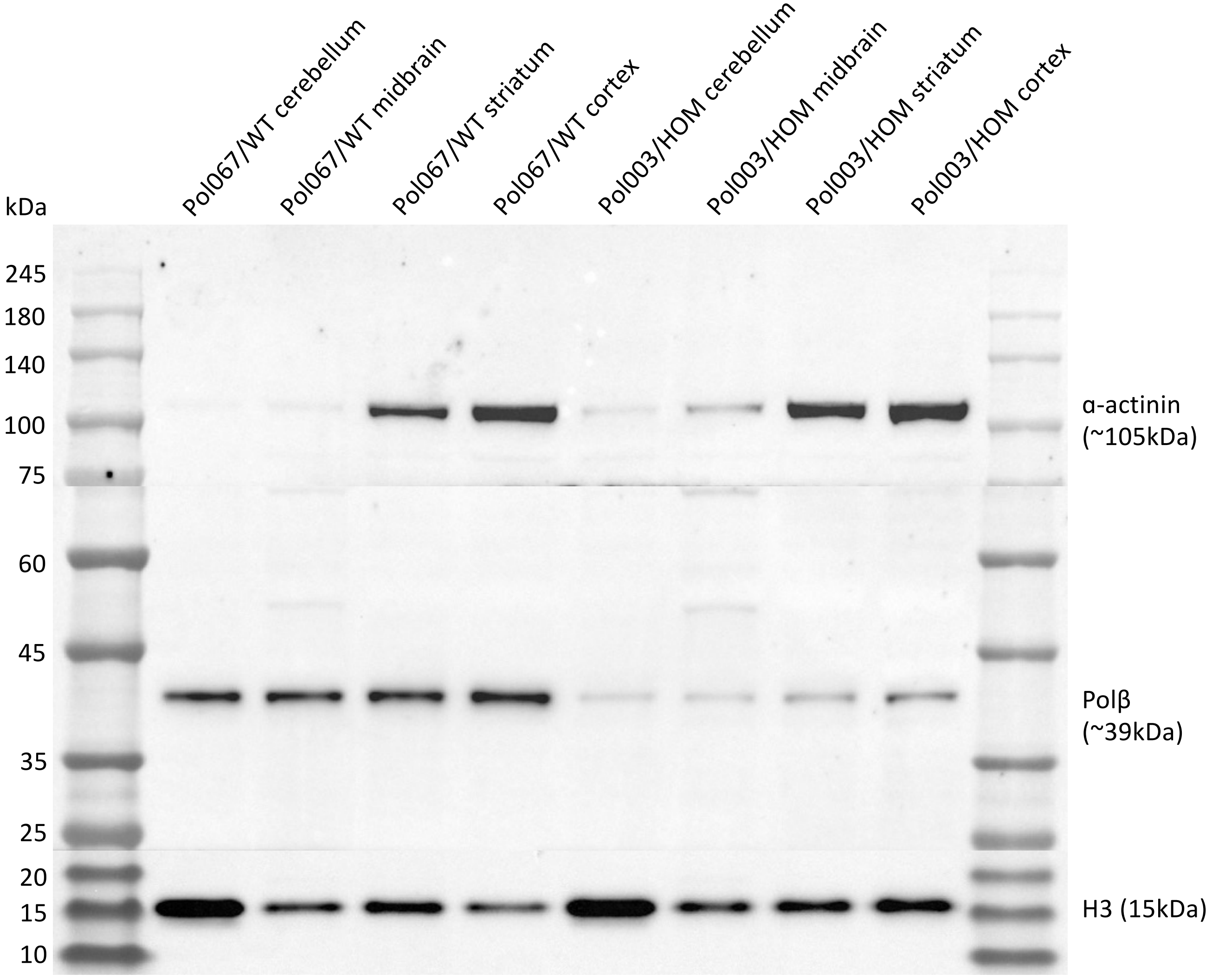

Loaded 30ug

Antibodies used:

- 1:1000 Anti-Polβ (Abcam, Cat# ab175197)
- 1:2000 Anti-H3 (Cell Signaling, Cat# 4499S)
- 1:2000 Anti-α-actinin (Cell Signaling, Cat# 6487S)

Pol067 x Pol003
Run #3: 7/22/25

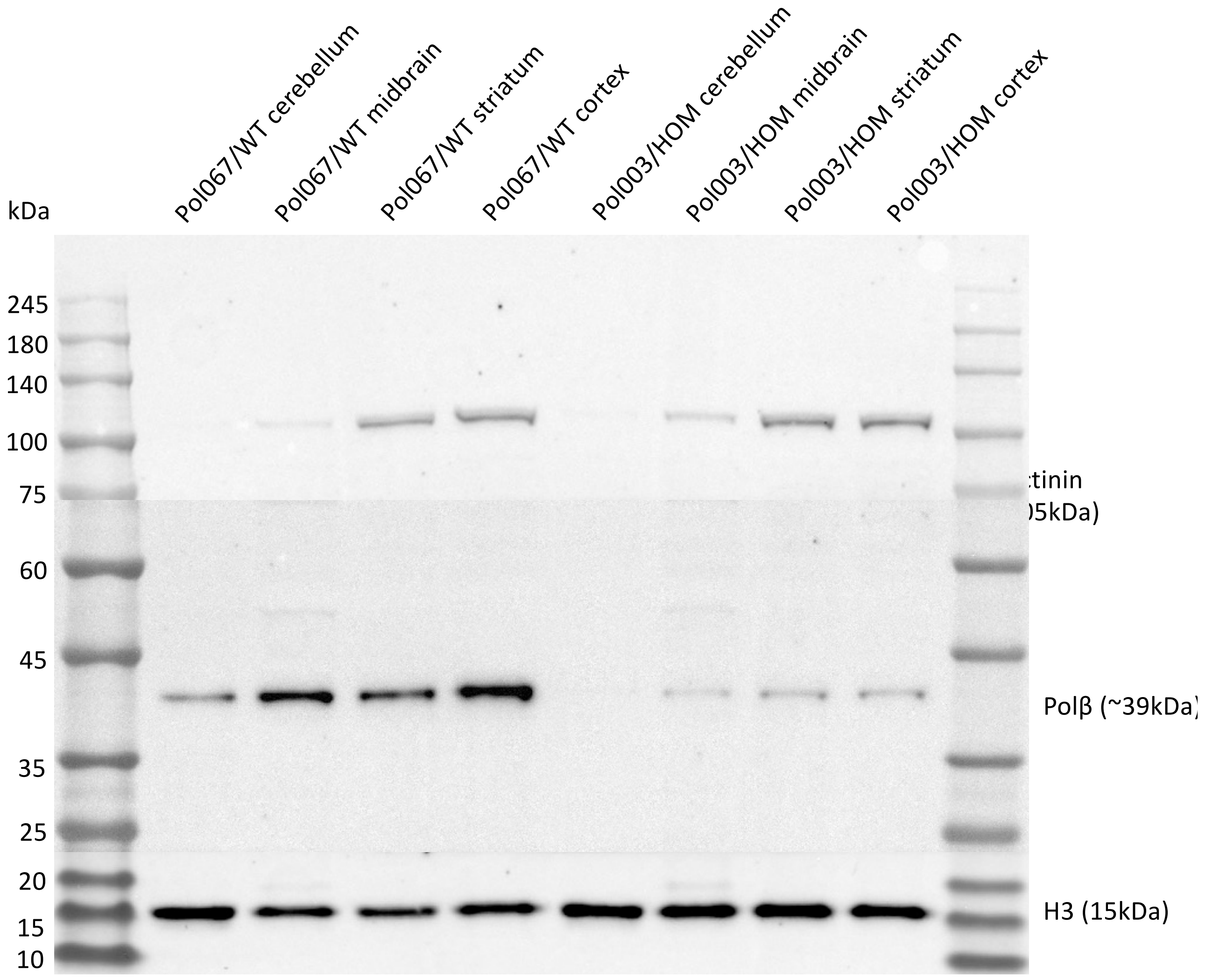

Supplement: Supplementary file 1 [file biomolecules-16-00412-s001.zip › biomolecules-4134159-supplementary.pdf]
